# Evaluation of antibody-based preventive alternatives for respiratory syncytial virus: a novel multi-criteria decision analysis framework and assessment of nirsevimab in Spain

**DOI:** 10.1186/s12879-024-08988-9

**Published:** 2024-01-18

**Authors:** Jorge Mestre-Ferrándiz, Agustín Rivero, Alejandro Orrico-Sánchez, Álvaro Hidalgo, Fernando Abdalla, Isabel Martín, Javier Álvarez, Manuel García-Cenoz, Maria del Carmen Pacheco, María Garcés-Sánchez, Néboa Zozaya, Raúl Ortiz-de-Lejarazu

**Affiliations:** 1 Department of Economics, University Carlos III, Madrid, Spain; 2 Department of Management, Bioregión de Salud y Bienestar (BioMad), Madrid, Spain; 3 Department of Vaccines Research, Fundación Para el Fomento de la Investigación Sanitaria y Biomédica de la Comunitat Valenciana (Fisabio), Valencia, Spain; 4 Catholic University of Valencia, Valencia, Spain; 5 Centro de Investigación Biomédica en Red de Epidemiología y Salud Pública (CIBERESP), Madrid, Spain; 6 Weber Foundation, Madrid, Spain; 7 Department of Economic Analysis and Finances, University of Castilla-La Mancha, Toledo, Spain; 8 Department of Health Affairs and Policy Research, Vivactis Weber, Madrid, Spain; 9 Department of Primary Care, Rochapea Healthcare Center, Navarra, Spain; 10 Department of Pediatrics, Hospital Costa del Sol, Málaga, Spain; 11 Public Health Institute of Navarra, Navarra, Spain; 12 Department of Epidemiology, General Directorate of Public Health, Castilla y León, Spain; 13 Department of Pediatrics, Nazaret Healthcare Center, Valencia, Spain; 14 Department of Quantitative Methods in Economics and Management, University Las Palmas de Gran Canaria, Las Palmas, Spain; 15 National Influenza Centre, School of Medicine, University of Valladolid, Castilla y León, Spain

**Keywords:** Respiratory Syncytial Virus, Prevention, Monoclonal antibodies, Multi-criteria decision analysis, MCDA

## Abstract

**Background:**

Respiratory syncytial virus (RSV) is a highly infectious disease that poses a significant clinical and medical burden, as well as social disruption and economic costs, recognized by the World Health Organization as a public health issue. After several failed attempts to find preventive candidates (compounds, products, including vaccines), new alternatives might be available, one being nirsevimab, the first and only option approved for RSV prevention in neonates and infants during their first RSV season. The objective of this study was to develop a novel multi-criteria decision analysis (MCDA) framework for RSV antibody-based preventive alternatives and to use it to assess the value of nirsevimab vs. placebo as a systematic immunization approach to prevent RSV in neonates and infants during their first RSV season in Spain.

**Methods:**

Based on a pre-established model called Vaccinex, an *ad-hoc* MCDA framework was created to reflect relevant attributes for the assessment of current and future antibody-based preventive measures for RSV. The estimated value of nirsevimab was obtained by means of an additive linear model combining weights and scores assigned by a multidisciplinary committee of 9 experts. A retest and three sensitivity analyses were conducted.

**Results:**

Nirsevimab was evaluated through a novel framework with 26 criteria by the committee as a measure that adds value (positive final estimated value: 0.56 ± 0.11) to the current RSV scenario in Spain, by providing a high efficacy for prevention of neonates and infants. In addition, its implementation might generate cost savings in hospitalizations and to the healthcare system and increase the level of public health awareness among the general population, while reducing health inequities.

**Conclusions:**

Under a methodology with increasing use in the health field, nirsevimab has been evaluated as a measure which adds value for RSV prevention in neonates and infants during their first RSV season in Spain.

**Supplementary Information:**

The online version contains supplementary material available at 10.1186/s12879-024-08988-9.

## Introduction

RSV is a highly infectious disease, with an unpredictable course and a significant clinical and medical burden, as well as social disruption and economic costs. Approximately 70% of children ≤ 12 months and 90% of children ≤ 2 years [1] became infected by this virus, asserting that a significant portion of the infant population faces a high risk of contracting this infectious agent. Clinic evolution of infant cases is difficult to predict, and can include lower respiratory tract infection (LRTI), such as pneumonia and bronchiolitis, entailing hospitalization, use of pediatric care units, oxygen support, palliative care or, in some cases, leading to death [2]. Furthermore, a significant proportion of cases occur in previously healthy infants, without underlying pathologies or at risk [3]. It is estimated that 43.2 out of every 1,000 children aged ≤ 1 are hospitalized with RSV (data from September 2017 until June 2018, in Spain) [4].

Over the past 60 years there have been several unsuccessful attempts to find candidates for RSV prevention or effective antiviral treatment for infants [5–8]. Thereby, RSV infections are a public health problem recognized by the World Health Organization (WHO), which advocates for a comprehensive RSV prevention strategy, preferably during the initial 15 months of life [9].

Decisions on the allocation of public health resources are complex, as they must incorporate patient access to innovations whilst safeguarding the financial sustainability of the system, in an environment of prominent demographic, technological, social, and budgetary challenges. Healthcare decision-makers use manifold tools to guide the decision-making process, such as economic evaluation and budget impact analysis. However, other factors are often also considered, such as severity of disease, availability of preventive and treatment alternatives, size of population affected, equity, social impact, quality of available evidence or degree of technological innovation.

In light of the aforementioned factors, the multi-criteria decision analysis (MCDA) is an instrument that yields ways of informing the preferences inherent to the decisions, in a consistent, explicit and transparent manner [10]. Despite the recent use of MCDA in health (it has been applied in other areas such as environment/waste, logistics/transportation, management, agriculture), this methodology has been implemented in practice in decision-making in many countries, including Spain [11–13].

In Spain, a new preventive measure for RSV (nirsevimab) has been recently approved [14]. Nirsevimab (Beyfortus®, Sanofi, Paris, France, and AstraZeneca AB, Södertälje, Sweden) is a recombinant human IgG1 kappa engineered monoclonal antibody that binds the F1 and F2 subunits of the RSV fusion (F) protein (antigenic site 0 of the protein in its pre-F form) to block viral entry into the host cell. It has been authorized by the European Medicines Agency (EMA) for the prevention of RSV LRTI in neonates and infants during their first RSV season, in October 2022 [14]. Moreover, other preventive measures (monoclonal antibodies, vaccines for infants, etc.) are expected to be introduced in the future [15]. All these interventions will require a specific evaluation framework.

MCDA assessments often necessitate the inclusion of a comparator for analysis. Presently, the prevention of RSV has only one available alternative, palivizumab, which is utilized in a mere 1.4% of the population in Spain [16, 17]. Palivizumab is authorized for preventing severe lower respiratory tract disease caused by RSV in children at high risk [18]. Consequently, opting for “placebo” as the comparator in this MCDA is the most appropriate choice, as it accurately represents the current standard of care in Spain. Thus, in the context of the ongoing MCDA in RSV, the term “placebo” should be understood as indicating no intervention.

The central objective of this research was to develop a novel MCDA framework for RSV antibody-based preventive alternatives and to assess the value of nirsevimab vs. placebo as a systematic immunization approach to prevent RSV in neonates and infants during their first RSV season in Spain.

To accomplish these objectives, we opted for Vaccinex [19] as the reference model, given its comprehensive nature in comparison to other identified frameworks used for evaluating preventive measures, such as vaccines [19–22]. Building upon this framework, we developed a tailored set of criteria adhering to the ISPOR guidelines on good practices [23]. These guidelines highlight the significance of selecting criteria that meet specific requirements, including completeness, non-redundancy, non-overlap, and preferential independence [23].

## Methods

### Study design

**Fig. 1:**
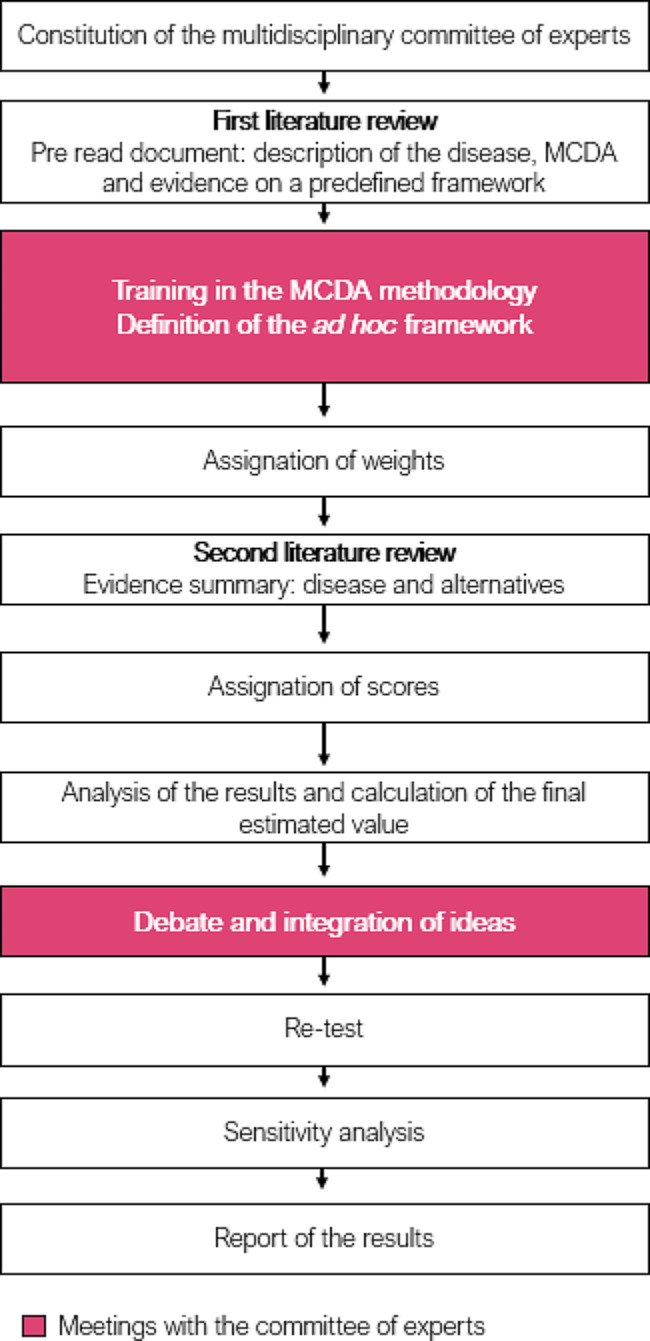
Study design

The following steps were undertaken in order to carry out this MCDA (Fig. 1): (1) a multidisciplinary committee of experts was constituted; (2) a first narrative review of the literature was performed, and as a result, a pre read document was elaborated (supplementary file 1); (3) experts were trained in the MCDA methodology; (4) based on the aforementioned pre-established framework (Vaccinex [19]) experts defined the framework for RSV, i.e. the set of criteria which should be used when evaluating any current or future antibody-based preventive measures in RSV; (5) experts weighted the selected criteria using the 1–5 scale method, thus making explicit their preferences on the relative importance of each one of them (where 1 means that the criterion is not very relevant in the evaluation of any preventive measure in this pathology, and 5, very relevant), regardless of the preventive measure to be evaluated; (6) a second narrative review of the literature was undertaken, resulting in an evidence summary document (supplementary file 2); (7) experts scored, online, individually, and blinded, aspects related to the pathology and the value contribution of nirsevimab vs. placebo in the prevention of RSV. For the absolute criteria (which do not compare the alternatives), the score ranged on a scale from 0 to 5, with 0 being the lowest value and 5 being the highest. For the relative criteria (which compare nirsevimab vs. placebo), the scale ranged from − 5 to 5 to reflect the full range of comparative effects; (8) an analysis of the results and the calculation of the final estimated value was realized; (9) the results of the scores and the final estimated value of nirsevimab vs. placebo were presented and discussed; (10) a retest of the weights and scores was performed; 11) three sensitivity analysis were conducted.

Main results derive from the test (Sect. 3.1–3.5), whilst the retest and sensitivity analyses are portrayed in a specific Sect. (3.6).

### Expert panel

This study was developed through a multidisciplinary committee of experts (MCE) formed by 9 members with different academic profiles. The choice of the MCE considered a balance between geographical representation (5 Autonomous Communities of Spain), the presence of different academic background (professionals in the field of pediatric, virology, preventive medicine, public health, epidemiology, government affairs and health economics), and the experience of the committee members in the management and prevention of respiratory virus and healthcare decision-making. Finally, an additional criterion for inclusion was the absence of conflict of interest from the MCE experts.

### Literature review

The information obtained by the two comprehensive non-systematic literature reviews were assembled into a pre read document (supplementary file 1) and an evidence summary (supplementary file 2), which were reviewed and validated by the clinicians from the MCE. The search was carried out in the main biomedical databases, such as PubMed, clinical trial registries, clinical practice guidelines, web pages of the official European and Spanish health assessment bodies, such as EMA, the Spanish Agency for Medicines and Health Products (AEMPS) or the health technology assessment agencies, as well as in grey literature sources. Publications in English and Spanish were included.

### Framework

The final framework consists of a set of selected criteria (see results section). Details such as how criteria were selected, the names of each criterion, the domains to which they belong, their definitions and their scoring scales, can be found in the supplementary files 3 and 4.

### Data analysis

An overall estimated value (ranging from − 1 to 1) was obtained for the comparison between nirsevimab and placebo, through an additive linear model of all individual criteria value contributions, which were calculated as the product of normalized weights and scores:

1 $$ V = \sum\limits_{x = 1}^n {{V_x}}= \sum\limits_{x = 1}^n {\left( {\frac{{{w_x}}}{{\sum {{W_n}} }}{S_x}} \right)}$$

where V is the total estimated value, Vx the value contribution of the criterion x, Wx the weighting of the criterion x, ∑Wn the sum of all weights, and Sx the normalized score of the criterion x (Sx = score / 5).

### Retest and sensitivity analysis

A retest and three sensitivity analysis were performed. Firstly, to evaluate the consistency, replicability, and internal validity of the results, a final estimated value was calculated with the results obtained by the retest and was contrasted with the base case value. The degree of agreement between the responses given at the two time points (test and retest) was evaluated by means of intra-rater correlation coefficients (ICC 3.1) using STATA® version 14 (STATA Corp., LP, College Station, TX, USA). Secondly, to assess the extent to which a change in the expert weights would affect the final estimated value of this MCDA, the expert weights were replaced with the original weights from the Vaccinex framework [19], and the impact on the final estimated result was analyzed. Thirdly, an outlier (measured by more than 1.5 times the central quartiles of the final estimated results) was excluded from the analysis, and this result was compared with the one from the base case. Finally, an evaluation of nirsevimab vs. palivizumab (a monoclonal antibody authorized for the prevention of serious lower respiratory tract disease requiring hospitalization caused by RSV in children at high risk for RSV disease [18, 24]) was also performed.

## Results

### Framework

Sixteen criteria were excluded from the Vaccinex framework (eradication potential, mode of transmission, image and goodwill, resistance offered by anti-vaccination groups, impact on the population of pregnant women, impact on school activities, time to development of symptoms, costs related to the production platform, productivity costs [presenteeism], generation of jobs in the country, impact on migrant population, impact on fertility, impact on the Lesbian, Gay, Bisexual, Transgender, and Intersex (LGTBI) population, impact on the female population, perception and fear, legal liability), and two criteria which were not present in Vaccinex were included (burden of disease – incidence on the outpatient setting; burden of disease – incidence on the inpatient setting). In addition, the disease prevalence criterion was renamed as “population in which the prevention strategy would be indicated”. The final framework for RSV assessment consists of 26 criteria (Tables 1 and supplementary files 3 and 4).

**Table 1:** Multi-criteria decision analysis framework for the evaluation of antibody-based preventive measures for respiratory syncytial virus infections

#	Criteria and domains	Type
**Domain 1: Severity of disease**		
1	Severity of symptoms	Absolute
2	Lethality risk	Absolute
3	Comorbidity risk	Absolute
**Domain 2: Burden of disease**		
4	Incidence of RSV cases	Absolute
5	Incidence on the outpatient setting	Absolute
6	Incidence on the inpatient setting	Absolute
7	Time of duration of acute symptoms	Absolute
**Domain 3: Prevention or Treatment Alternatives**		
8	Prevention alternatives	Absolute
9	Availability of treatment	Absolute
**Domain 4: Size of population**		
10	Population in which the prevention strategy would be indicated	Relative
**Domain 5: Efficacy**		
11	Efficacy of the preventive measure	Relative
**Domain 6: Population protection**		
12	Group immunity (collective protection)	Absolute
13	Transmissibility	Absolute
**Domain 7: Safety**		
14	Serious adverse events	Relative
15	Mild adverse events	Relative
**Domain 8: Quality of evidence**		
16	Certainty about the efficacy of the preventive measure	Absolute
**Domain 9: Impact on quality of life**		
17	Impact on the population of children	Absolute
18	Impact on the population over 65 years of age	Absolute
19	Impact on caregivers	Absolute
**Domain 10: Acquisition cost**		
20	Monetary cost of the preventive measure	Relative
**Domain 11: Impact on other costs**		
21	Cost of the disease on the health system (excludes acquisition cost)	Relative
22	Productivity cost: absenteeism	Relative
23	Cost of the disease on the patient (out-of-pocket expenses)	Relative
**Domain 12: Social benefits**		
24	Impact on health inequity	Relative
25	Public health awareness (including antibiotic resistance)	Relative
26	Innovation stimulus	Absolute

RSV: respiratory syncytial virus

### Weights

**Fig. 2:**
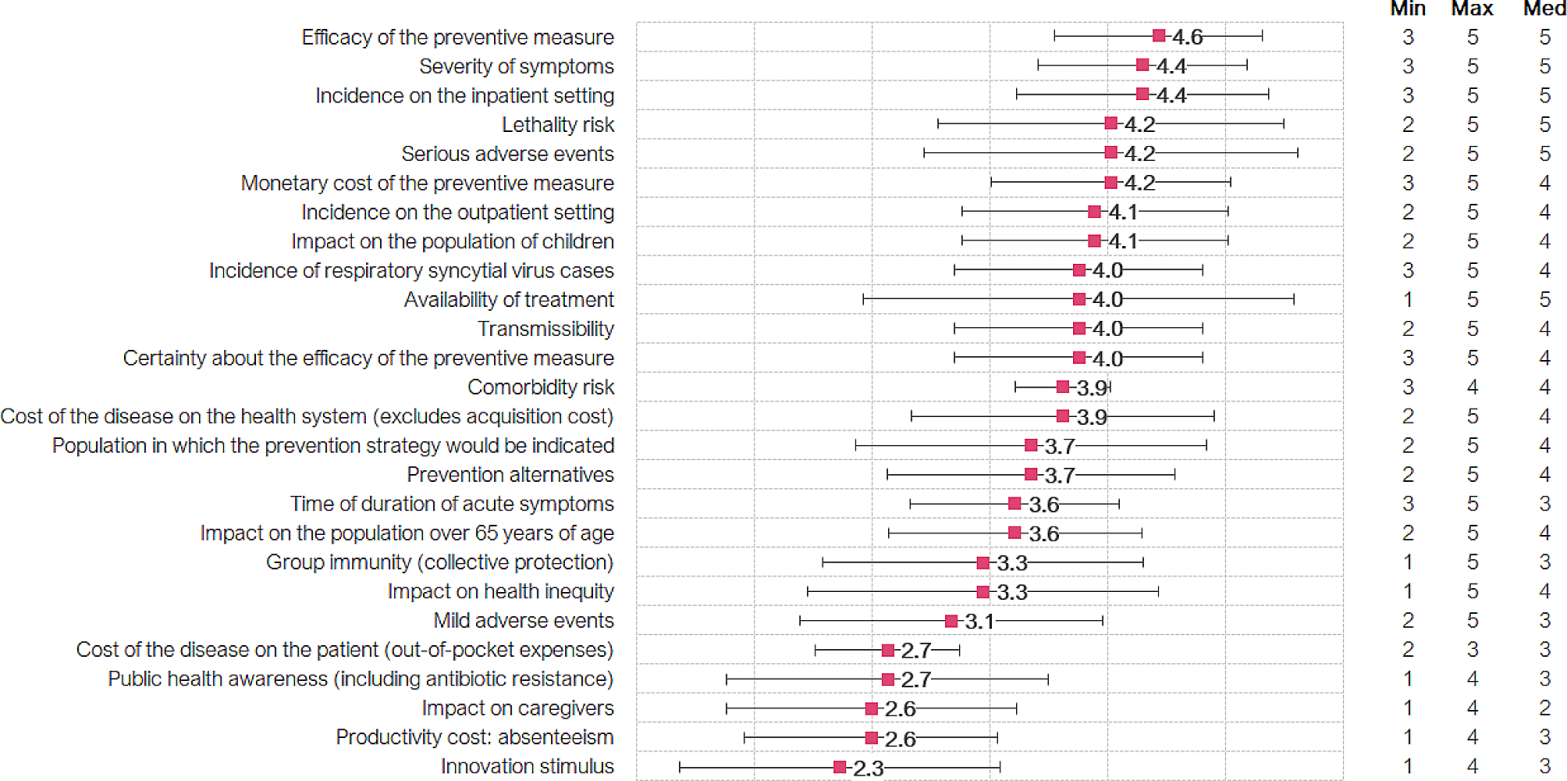
Relevance of each individual criterion in the assessment of any antibody-based preventive measures for respiratory syncytial virus (mean, min, max and median weights, and standard deviations). The 1–5 scale method was applied, where 1 means that the criterion is not very relevant in the evaluation of any preventive measure in this pathology, and 5 means that it is a very relevant criterion

Based on the mean weights given by the experts, the five criteria considered as most relevant to any evaluation of antibody-based preventive measures in RSV were efficacy of the preventive measure (4.6 ± 0.7 out of 5.0), severity of symptoms (4.4 ± 0.7), incidence on the inpatient setting (4.4 ± 0.9), lethality risk (4.2 ± 1.2) and serious adverse events (4.2 ± 1.3), while those of lesser relevance were innovation stimulus (2.3 ± 1.1), impact on productivity cost (2.6 ± 0.9), impact on caregivers (2.6 ± 1.0), public health awareness (2.7 ± 1.1) and cost of the disease on the patient (out of pocket expenses) [2.7 ± 0.5] (Fig. 2). See supplementary file 5 for detailed weights, overall and by subgroups.

### Absolute scores

**Fig. 3:**
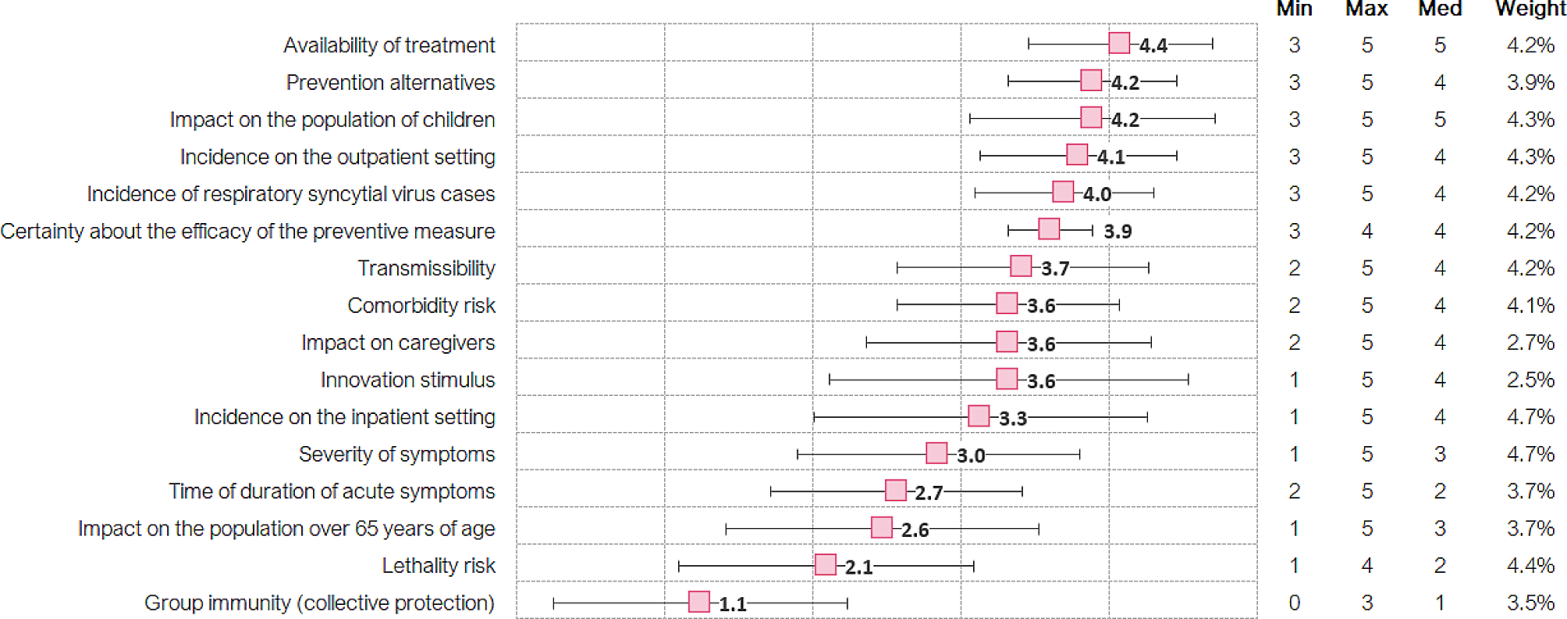
Scores assigned per absolute criterion (mean, standard deviations, min, max, median scores, and weights). Scale from 0 to 5

The examination of the absolute scores (Fig. 3 and Table 2) suggests that RSV is a disease with no effective alternative treatment and with a prevention alternative which is very limited (mean scores, availability of treatment: 4.4 ± 0.7 out of 5.0; prevention alternatives: 4.2 ± 0.7). The only antiviral treatment available (ribavirin®) can only be administered in severe cases, requires aerosolization in specific devices and its routine use is not recommended [25, 26]. The only prevention alternative, palivizumab®, is only indicated for high-risk premature infants and in a hospital setting [18, 27].

**Table 2:** Mean scores and key comments from the multidisciplinary committee of experts or based on evidence, for each absolute criterion

MCDA framework criteria	Mean score ± SD	Main comments from the multidisciplinary committee of experts or evidence available
**Domain 1: Severity of disease**		
1. Severity of symptoms	3.0 ± 1.1	• Moderate symptoms, that usually last for three to seven days (not very long lasting)^**i**^ • In cases with symptoms, RSV is one of the most serious pathologies in pediatric care • RSV is a less serious disease than others, such as ovarian or pancreatic cancer
2. Lethality risk	2.1 ± 1.2	• Not very lethal • Death rate for children with bronchiolitis (main diseases caused by RSV) ≤ 2 years of 82/100,000^**ii**^
3. Comorbidity risk	3.6 ± 0.9	• RSV-infected children are at high risk of developing comorbidities (short, medium, and long-term) • Some experts have scored based on long-term symptoms (asthma, etc.) • Others indicated that the susceptibility of the subject who requires hospitalization at a very early age is what predisposes the existence of comorbidities
**Domain 2: Burden of disease**		
4. Incidence of RSV cases	4.0 ± 0.7	• High/very high incidence of RSV cases • Approximately 65% of children ≤ 12 months and 90% of children < 2 years become infected with RSV^**iii**^
5. Incidence on the outpatient setting	4.1 ± 0.8	• High/very high outpatient incidence • Primary care: 39,690/100,000 children ≤ 12 months (averaging 9.0 visits per infected patient)^**iv**^ • Specialty care: 882/100,000 children ≤ 12 months (averaging 2.0 visits per infected patient)^**v**^
6. Incidence on the inpatient setting	3.3 ± 1.3	• High inpatient incidence^**vi**^ • Children arriving on the inpatient setting are anticipating a serious admission • Complicated cases to treat • Concentrated in certain months of the year (season), which makes the management of these admissions relatively more complicated than other types of illnesses
7. Time of duration of acute symptoms	2.7 ± 1.0	• Short duration of acute RSV symptoms • The extreme case (who scored 5) pointed out that a child with RSV bronchiolitis will continue to present clinical consequences of bronchial hyperresponsiveness for the rest of the season, and therefore the persistence of symptoms will be much greater than it seems • Average hospital stay is less than 6 days (some experts consider 8 days as a threshold for a long stay)^**vii**^
**Domain 3: Prevention or Treatment Alternatives**		
8. Prevention alternatives	4.2 ± 0.7	• There are no effective prevention alternatives against RSV • The only prevention alternative, palivizumab^®^, is only indicated for a small group of children (infants born prematurely and/or with heart or lung disease) and on an inpatient setting^**viii**^
9. Availability of treatment	4.4 ± 0.7	• There is no effective treatment alternative for RSV infection • The only antiviral treatment, ribavirin^®^, can only be given in severe cases, and its routine use is not recommended^**ix**^
**Domain 6: Population protection**		
12. Group immunity (collective protection)	1.1 ± 1.2	• Collective protection would only be achieved if nirsevimab was applied to a very large share of the infant population • Collective protective effect of nirsevimab will only really be known once it is implemented in routine clinical practice
13. Transmissibility	3.7 ± 1.0	• It is a highly transmissible pathology (R0 = 4.5)^**x**^ • Children are much more likely to be infected than adults^**xi**^
**Domain 8: Quality of Evidence**		
16. Certainty about the efficacy of the preventive measure	3.9 ± 0.3	• Evidence is very relevant and there is a lot of certainty about the efficacy of nirsevimab
**Domain 9: Impact on quality of life**		
17. Impact on the population of children	4.2 ± 1.0	• RSV has a very high impact on the stress levels and quality of life of infected children • Evidence indicates quality of life losses of 39% in children after diagnosis, and stress levels of 79% after hospital discharge^**xii**^
18. Impact on the population over 65 years of age	2.6 ± 1.2	• RSV has a low impact on stress levels and quality of life on the population over 65 years of age • Evidence indicates quality of life losses of 11% on the population over 65 years age infected by RSV within a week of symptom onset^**xiii**^
19. Impact on caregivers	3.6 ± 1.1	• RSV has a high impact on the stress levels and quality of life of caregivers, who need to spend considerable hours caring for infected children • Caregivers of preterm infants spend, on average, 282 h for hospital visits vs. 140 h for caregivers of children born at term^**xiv**^ • The stress level of parents of children ≤ 12 months hospitalized for RSV is 83% at hospital discharge (5.8 on a scale of 7) and 34% one month later (2.4 on a scale of 7)^**xv**^
**Domain 12: Social benefits**		
26. Innovation stimulus	3.6 ± 1.4	• The score reflects that the funding of nirsevimab will lead more laboratories to continue producing innovations that improve population health

RSV: respiratory syncytial virus. Note: there is a mix between comments from the multidisciplinary committee of experts, and evidence found in the literature. All bibliographic references can be found in the main body of the article or in the supplementary file 2 (evidence summary). **i**: references [23] and [27] of the supplementary file 2. **ii**: reference [33]. **iii**: references [19] and [20]. **iv**: reference [4]. **v**: reference [22] of the supplementary file 2. **vi**: reference [22]. **vii**: reference [23] of the supplementary file 2. **viii**: references [18] and [27]. **ix**: references [25] and [26]. **x**: reference [30]. **xi**: reference [40] of the supplementary file 2. **xii**: references [31] and [32]. **xiii**: reference [50] of the supplementary file 2. **xiv**: reference [51] of the supplementary file 2. **xv**: reference [45] of the supplementary file 2

Furthermore, children with a disease caused by an infection by RSV are at high risk of developing comorbidities in the short, medium, and long term (mean score comorbidity risk: 3.6 ± 0.9). Additionally, the incidence of RSV is very high (mean score incidence of RSV cases: 4.0 ± 0.7), with infections occurring in 90% of children within their first 2 years of life [1, 28]. This translates into a very high burden of disease, inappropriate consumption of antibiotics and healthcare resources, as reflected by its inpatient and outpatient incidence (39,690 RSV cases/100,000 children ≤ 1 years, 9 medical visits per infected patient [4]) vs. (2,520 hospitalizations for RSV/100,000 children ≤ 2 years [29]), respectively (mean scores: incidence on the inpatient setting: 3.3 ± 1.3; incidence on the outpatient setting: 4.1 ± 0.8).

Moreover, it is a highly transmissible pathology (R0 = 4.5 [30], mean score transmissibility: 3.7 ± 1.0), with a high impact on the population of children (mean score: 4.2 ± 1.0), who present losses in quality of life after diagnosis and stress levels after hospital discharge of 39% and 79%, respectively [31, 32].

Finally, bronchiolitis (one of the main diseases caused by RSV) has a rate of 82 deaths per every 100,000 children ≤ 2 years hospitalized [33], which is not considered as very high by the experts (mean score lethality risk: 2.1 ± 1.2). In addition, it presents moderate symptoms (mean score severity of symptoms: 3.0 ± 1.1) that usually last for three to seven days, which is regarded as not very long lasting by the MCE (mean score time of duration of acute symptoms: 2.7 ± 1.0). See supplementary files 5 and 6 for detailed absolute scores.

### Relative scores

**Fig. 4:**
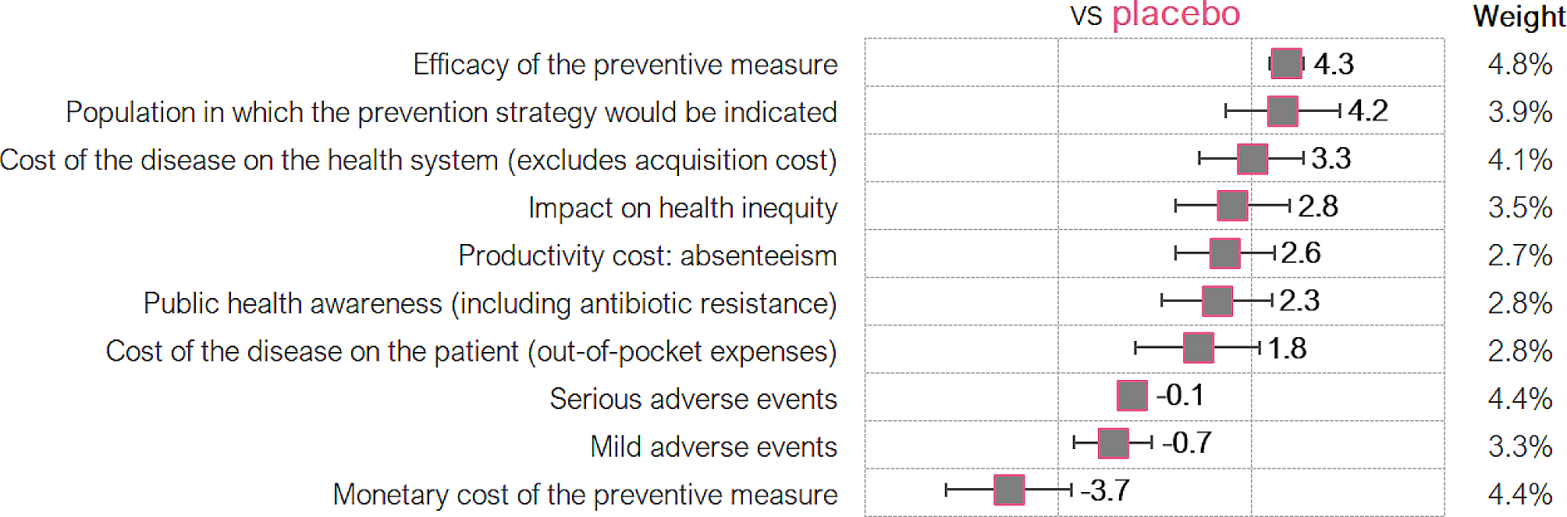
Scores assigned per relative criterion (mean, standard deviations, and weights). Scale from − 5 to 5

Overall, relative scores (Fig. 4 and Table 3) indicate that nirsevimab was considered by the MCE as a preventive measure with *clinical benefits*, as it is much more effective than placebo (80% efficacy in preventing medically attended RSV-associated LRTI [34]; mean score efficacy of the preventive measure: 4.3 ± 0.5 out of 5.0), with a robust safety profile (safety outcomes associated with nirsevimab are comparable to those observed with placebo) (mean score serious adverse events: -0.1 ± 0.3).

**Table 3:** Mean scores and key comments from the multidisciplinary committee of experts or based on evidence, for each relative criterion, nirsevimab vs. placebo

MCDA framework criteria	Mean score ± SD	Main comments from the multidisciplinary committee of experts or evidence available
**Domain 4: Size of population**		
10. Population in which the prevention strategy would be indicated	4.2 ± 1.6	• Nirsevimab would be indicated for a much broader infant population than placebo • Nirsevimab was authorized by EMA for the prevention of RSV lower respiratory tract disease in neonates and infants during their first RSV season^**i**^
**Domain 5: Efficacy**		
11. Efficacy of the preventive measure	4.3 ± 0.5	• Nirsevimab is much more effective than placebo, with an 80% efficacy in preventing medically attended RSV-associated lower respiratory tract infection^**ii**^
**Domain 7: Safety**		
14. Serious adverse events	-0.1 ± 0.3	• Very similar safety profile vs. placebo^**iii**^ • Reported serious adverse events: 6.8% nirsevimab vs. 7.3% placebo (MELODY)^**iii**^
15. Mild adverse events	-0.7 ± 1.1	• Very similar safety profile vs. placebo^**iii**^ • Some experts commented that biologics are often more reactogenic than placebo • Others also considered the risk of having to administer a monoclonal antibody (or any biologic) to a newborn • Additionally, some experts have commented that, given that the preventive measure is administered to such young children, the traditional “nocebo effect” would not exist in this case
**Domain 10: Acquisition cost**		
20. Monetary cost of the preventive measure	-3.7 ± 1.8	• Although the price of nirsevimab is still unknown, for the purpose of this exercise, it was benchmarked with the price of innovative vaccines in Spain • Experts granted it a negative scored on monetary cost of the preventive measure, in relation to placebo (no intervention)
**Domain 11: Impact on other costs**		
21. Cost of the disease on the health system (excludes acquisition cost)	3.3 ± 1.5	• There was consensus (> 85% positive scores) that the implementation of nirsevimab vs. placebo would generate savings in other health system costs, such as hospitalizations, outpatient consultations, or emergency room care • The scores were awarded, in general, considering that the application of nirsevimab would generate a delay in infections, which would lead to a lesser severity of the disease and a lower use of resources • This would be due to the mechanism of action of nirsevimab, which allows children to come into contact with the virus but does not allow the attachment of the virus to the epithelium
22. Productivity cost: absenteeism	2.6 ± 1.4	• There was consensus (> 85% positive scores) that the implementation of nirsevimab vs. placebo would improve the labor productivity of caregivers, generating savings in costs related to absenteeism • No specific evidence was available for this criterion. According to a US study, the overall work productivity loss, absenteeism and presenteeism of mothers of infants ≤ 12 months preterm (29–35 weeks gestational age) hospitalized for RSV infection was 91% (total productivity), 73% (absenteeism) and 64% (presenteeism) at hospital discharge and 31% (total productivity), 16% (absenteeism) and 23% (presenteeism) at one month of hospital discharge^**iv**^
23. Cost of the disease on the patient (out-of-pocket expenses)	1.8 ± 1.8	• There was consensus (> 85% positive scores) that the implementation of nirsevimab vs. placebo would generate savings in patients’ out-of-pocket expenses • No specific evidence was available for this criterion. A North American study estimated average out-of-pocket expenses of $643 for hospitalized preterm infants and $214.42 for hospitalized term infants. These expenses consist of transportation, parking, food, day care, and other expenses^**v**^
**Domain 12: Social benefits**		
24. Impact on health inequity	2.8 ± 1.6	• The universal recommendation of nirsevimab would result in the elimination of health inequity, contributing to avoiding RSV infections in the most disadvantaged populations, for whom RSV represents a greater burden in terms of comorbidity and mortality • By avoiding disease, poverty is avoided, and by avoiding poverty, disease is avoided (Horwitz’s circles)
25. Public health awareness (including antibiotic resistance)	2.3 ± 1.6	• The use of nirsevimab in comparison to placebo would translate into an increase in public health awareness through greater social debate and greater parental attention in the case of introducing this preventive measure in the children vaccination schedule • Many experts also agree that a preventive measure on a prevalent childhood infection will reduce the inappropriate use of antibiotics

RSV: respiratory syncytial virus. EMA: European Medicines Agency. Note: there is a mix between comments from the multidisciplinary committee of experts, and evidence found in the literature. All bibliographic references can be found in the main body of the article or in the supplementary file 2 (evidence summary). **i**: reference [14]. **ii**: reference [37] of the supplementary file 2. **iii**: reference [1] of the supplementary file 2. **iv**: reference [45] of the supplementary file 2. **v**: reference [62] of the supplementary file 2

This clinical benefit, added to the fact that nirsevimab is indicated for neonates and infants during their first RSV season (mean score population in which the prevention strategy would be indicated: 4.2 ± 1.6), is also associated with several economic and social benefits.

To assess the *economic benefits*, since the price of nirsevimab was unknown at the time of the study, and for the purpose of this exercise, it was benchmarked with the price of innovative vaccines in Spain. Thereby, experts granted a negative score on the monetary cost of the preventive measure, in relation to placebo (-3.7 ± 1.8).

Furthermore, there was consensus (> 85% positive scores) that the implementation of nirsevimab vs. placebo would generate savings in other health system costs, such as hospitalizations, outpatient consultations, or emergency room care (mean score cost of the disease on the health system: 3.3 ± 1.5); that it would improve the labor productivity of caregivers, generating savings in costs related to absenteeism (mean score productivity cost – absenteeism: 2.6 ± 1.4); and in patients’ out-of-pocket expenses (mean score cost of the disease on the patient: 1.8 ± 1.8).

Moreover, the *social benefits* of using nirsevimab in comparison to placebo would translate, on the one hand, into an increase in public health awareness through greater social debate and greater parental attention in the case of introducing this preventive measure in the children childhood immunization schedule (mean score public health awareness: 2.3 ± 1.6). On the other hand, its universal recommendation would result in the elimination of health inequity (mean score impact on health inequity: 2.8 ± 1.6), contributing to avoiding RSV infections in the most disadvantaged populations, for whom RSV represents a greater burden in terms of comorbidity and mortality (see supplementary files 5 and 6 for detailed relative scores).

### Final estimated value

**Fig. 5:**
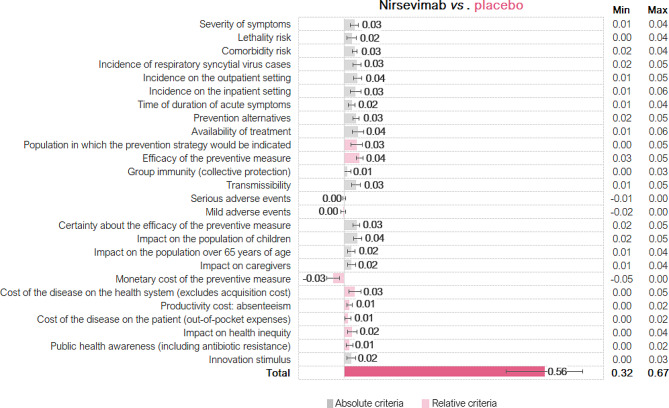
Value contribution of nirsevimab compared to placebo according to the multi-criteria decision analysis framework for the assessment of antibody-based preventive measures for respiratory syncytial virus (mean, standard deviation, min, max)

The final estimated value (overall means, *n* = 9) obtained in this MCDA in RSV was 0.56 ± 0.11 (0.32–0.67) for the comparison between nirsevimab and placebo (Fig. 5), meaning that the introduction of nirsevimab would provide positive value in the view of the MCE.

Out of this total, 0.43 was related to the absolute criteria, whilst 0.13 was associated with the relative criteria. Individually, the criteria with the highest contribution to the final estimated value were efficacy of the preventive measure (7.5% of total value), availability of treatment (6.7%), impact on the population of children (6.5%), incidence on the outpatient setting (6.5%), and incidence of RSV cases (6.1%). See supplementary file 5 for detailed results.

### Retest and sensitivity analysis

The consistency of the weights between test and retest was high, with an average intra-rater correlation coefficient (ICC) of 0.8366. Likewise, the retest scores and value estimates were very similar to the test, with average ICCs of 0.9189 and 0.9196, respectively. The retest value estimate was 4.2% higher than the test´s.

When replacing the original weights of this MCDA in RSV with the weights assigned in the Vaccinex study, the impact on outcomes was close to none, with final estimated values ranging from 0.55 to 0.56 (vs. 0.56 in the MCDA in RSV).

By excluding the outlier, the final estimated value increased 5.4%, resulting in 0.59. See supplementary file 7 for more details.

An alternative evaluation comparing nirsevimab vs. palivizumab was also undertaken in the context of RSV prevention in neonates and infants during their first RSV season, obtaining very similar results (final estimated value of 0.58). The criteria which mark the distinction between these two comparisons were the efficacy of the preventive measure (nirsevimab has an estimated value of 0.04 vs. placebo and 0.02 vs. palivizumab) and its monetary cost (nirsevimab obtained an estimated value of -0.03 vs. placebo, and 0.02 vs. palivizumab) (see supplementary file 8).

## Discussion

Complex decision contexts, such as evaluations of new preventive measures for diseases with many important unmet needs, require evaluators and decision makers to balance multiple needs and ways to address them. The main methodological approaches currently used to support evaluations are budget impact and efficiency analysis models [35].

However, the MCDA methodology can be particularly useful as a complement to this approach, as it consists of a structured (stepwise validated methodology), multidimensional (participation of experts from different professional fields), transparent (criteria, weights and scores are explicit) and systematic (replicable) approach, which incorporates several criteria and their individual value contribution to the decision or resource allocation problem [36]. Its popularity has been evident in the healthcare field in recent years, both nationally and internationally [36–39], in various areas, such as oncology [40–42], rare diseases [43–45], dermatology [46–48], and in the field of decisions related to the implementation of vaccines [19, 20, 22, 49–52].

This MCDA has adopted a holistic and transparent methodological approach in the evaluation of the value contribution of nirsevimab compared to placebo for the prevention of RSV in neonates and infants during their first RSV season in Spain, through a multidisciplinary panel of experts involved in the clinical, management and decision-making aspects of the pathology.

Preventive measures have some characteristics that are different from most drugs and health technologies, such as the ability to eradicate disease and more holistic social effects; therefore, it is essential to evaluate them according to other broader criteria [19–22, 49–52]. To this end, the Vaccinex framework [19] was adapted into a set of 26 criteria relevant to the context of evaluating new (present or future) antibody-based preventive measures for RSV. Based on the mean weights assigned to each criterion in the current exercise, the importance of applying a broader list of attributes became clear, as criteria related to the disease, unmet needs and social impacts represented a total weight of 58% in the current MCDA framework.

Other MCDA have been carried out in the field of vaccine evaluation and prioritization [19, 20, 22, 49–52]. However, these studies are carried out in different contexts (other countries, such as Bangladesh and Indonesia; evaluation of vaccines and not preventive measures, etc.) and using methodologies different from that applied in this MCDA in RSV (fewer criteria, and some of them interrelated, such as quality-adjusted life years [QALYs] and cost per QALY; different weighting methods [100-point distribution, rank-order centroid method], scoring [100% objective]), so they are not comparable.

The exercise performed in this MCDA in RSV found that nirsevimab adds value to the prevention of RSV in neonates and infants during their first RSV season in Spain, compared to placebo. The final estimated value in this MCDA was 0.56. This value is higher when compared to several other MCDA focused on the value of innovative drugs [43, 53–58]. Conjointly with the positive final estimated value obtained, the importance of this MCDA lies in understanding the value drivers of the preventive measure evaluated. In this sense, the multidisciplinary debate generated was key to understanding the strengths and weaknesses of nirsevimab compared to placebo in each of the elements considered.

Nirsevimab was perceived as an effective measure in the prevention of medically attended RSV infections (> 80%, outpatient and inpatient setting [34]), with a remarkably robust safety profile (safety outcomes associated with nirsevimab are comparable to those observed with placebo [59]). In addition, nirsevimab would be indicated for a much larger number of infants (nirsevimab in neonates and infants during their first RSV season vs. placebo). Moreover, its implementation could generate savings in hospitalization costs in Spain (50% or 30 million euros [17]), and greater public health awareness (generating a debate in society and increased parental awareness in relation to RSV infections). This, in turn, would contribute to diminishing health disparities among newborns and infants in their initial RSV season, consequently lowering the prevalence of RSV infections in poor regions.

These effects would be given for a disease with currently no available treatments or prevention alternatives, of high incidence (90% <2 years are infected [1]), very contagious [30] and with an important risk of hospitalization [60]. Moreover, despite the short duration of the acute symptoms of RSV infections, according to the MCE, there is a considerable percentage of mid-long-term consequences of bronchial hyperreactivity (bronchial wheezing, asthma) caused by this infection. Finally, RSV has a great impact in the quality of life and stress levels of the affected infants and their carers [31, 32].

This study has some limitations, inherent to all MCDA, which should be pointed out. The first comes from the composition of the expert committee itself, as other important stakeholders are not represented (e.g., mother, pregnant women). Similarly, it should be borne in mind that the final assessment of the MCDA comes largely from the experience, training, and value judgments of the committee members, so this type of tool is associated with a certain subjectivity. However, this is intrinsic to most decision-making processes in healthcare, and the development and use of MCDA is not intended to provide an objective ratio or a single answer to a problem requiring a decision but should be used as a complementary tool to the existing set of tools and frameworks.

In this context, it is crucial to underscore that certain criteria, such as “Costs related to the production platform,” were excluded based on expert evaluations. This specific criterion fell below the predefined threshold for inclusion in the framework, as only a minority of experts advocated for its integration. The decision to exclude it is underpinned by its connection to implementation feasibility, particularly in the context of integrating with existing vaccinations. The framework prioritizes criteria that garner broader consensus, ensuring a robust and widely accepted assessment.

Similarly, the criterion “Perception and fear of RSV” elicited diverse opinions within the expert panel and failed to meet the inclusion threshold. Subsequent discussions provided clarity on the interpretation of this criterion, ultimately leading to the decision not to incorporate it into the MCDA framework. The committee justified this exclusion based on the absence of consensus and the varied interpretations among experts.

Furthermore, the analysis entails some cognitive complexity, especially considering that this MCDA was performed with 26 criteria. Moreover, systematic literature reviews were not carried out for the creation of the pre read and evidence summary documents. Nonetheless, a comprehensive narrative review was undertaken, and all material was validated by the clinicians of the MCE, hence reducing the probabilities of any gap or inconsistency in the information set used as a base for the assessment. Finally, the evidence summary document gathered publicly available information at the time of the study: some evidence were not available (i.e., price of nirsevimab) and some were scarce (i.e., impact on other direct and indirect costs). Hence, results could show differences in light of additional information and evidence.

The strengths of this study lie in several aspects. First, it should be noted that, to our knowledge, this is the first MCDA to develop an *ad hoc* framework that can be applied in the assessment of any antibody-based preventive measures (present and future) for RSV infections, and to evaluate nirsevimab against placebo for those infections. Additionally, the scoring was preceded by a thorough explanation of the MCDA methodology, the assumptions made and the interpretation of the values, and the exercise was followed by the implementation of a retest to assess the consistency of the results. Finally, the evidence summary was based on a thorough review of the available information, which is fundamental for this type of exercise.

## Conclusions

Unmet needs in the prevention of RSV infections and severe disease in neonates and infants during their first RSV season in Spain are still substantial. Moreover, the burden of disease (ambulatory, emergency room and hospital incidence) is very high, and there are no effective treatments or preventive alternatives available. Under a methodology of increasing use in the healthcare setting such as MCDA, nirsevimab has been evaluated by a MCE as a measure that brings added value to the current scenario of RSV prevention in Spain, by providing efficacy, with a robust safety profile (safety outcomes associated with nirsevimab are comparable to those observed with placebo) and being indicated for a much wider infant population. This might generate several economic and social benefits, such as cost savings in hospitalizations and to the healthcare system in general, an increase in the level of public health awareness towards RSV infections, and a reduction in health inequities which are inherent to respiratory infections. This type of exercise allows us to understand where the value of preventive measures lies for the different agents, encourages communication between them and can serve as a reference in decision-making on evaluation, financing, and reimbursement. In the future, it would be desirable to continue advancing in the development of the MCDA methodology and to extend its use, so that health care decision-making is carried out in a framework of greater transparency, consistency, and efficiency.

### Electronic supplementary material

Below is the link to the electronic supplementary material.

**Supplementary Material 1:** Pre read

**Supplementary Material 2:** Evidence summary

**Supplementary Material 3:** Criteria selection

**Supplementary Material 4:** Framework

**Supplementary Material 5:** Weights, scores and value estimates

**Supplementary Material 6:** Detailed scores per criterion

**Supplementary Material 7:** Retest and sensitivity analysis

**Supplementary Material 8:** Nirsevimab vs. Palivizumab

## Data Availability

The datasets used and/or analyzed during the current study are available from the corresponding author on reasonable request.

## Abbreviations

AEMPS: Spanish Agency for Medicines and Health Products
EMA: European Medicines Agency
ICC: Intra-rater correlation coefficients
LGBTI: Lesbian, Gay, Bisexual, Transgender, and Intersex
LRTI: Lower respiratory tract infections
MCDA: Multi-criteria decision analysis
MCE: Multidisciplinary committee of experts
QALY: Quality-adjusted life year
RSV: Respiratory syncytial virus
WHO: World Health Organization
