# Supplementary material for: Evaluation of antibody-based preventive alternatives for respiratory syncytial virus: a novel multi-criteria decision analysis framework and assessment of nirsevimab in Spain
Source: BMC Infect Dis. 2024 Jan 18;24:99. doi: 10.1186/s12879-024-08988-9 (PMC10797756; doi:10.1186/s12879-024-08988-9)
Supplement: Supplementary file 1 — Supplementary Material 1: Pre read [file 12879_2024_8988_MOESM1_ESM.docx]

**Evaluation of Antibody-based Preventive Alternatives for Respiratory Syncytial Virus: A Novel Multi-Criteria Decision Analysis Framework and Assessment of Nirsevimab in Spain**

**Authors**: Jorge Mestre-Ferrándiz^1^, Agustín Rivero^2^, Alejandro Orrico-Sánchez^3,4,5^, Álvaro Hidalgo^6,7^, Fernando Abdalla^8^, Isabel Martín^9^, Javier Álvarez^10^, Manuel García-Cenoz^11^, Maria del Carmen Pacheco^12^, María Garcés-Sánchez^13^, Néboa Zozaya^8,14^, Raúl Ortiz-de-Lejarazu^15^

**Affiliations**: ^1^Department of Economics, University Carlos III, Madrid, Spain; ^2^Department of Management, Bioregión de Salud y Bienestar (BioMad), Madrid, Spain; ^3^Department of Vaccines Research, Fundación Para el Fomento de la Investigación Sanitaria y Biomédica de la Comunitat Valenciana (Fisabio), Valencia, Spain; ^4^Catholic University of Valencia, Spain; ^5^Centro de Investigación Biomédica en Red de Epidemiología y Salud Pública (CIBERESP); ^6^Weber Foundation, Madrid, Spain; ^7^Department of Economic Analysis and Finances, University of Castilla-La Mancha. Toledo, Spain; ^8^Department of Health Affairs and Policy Research, Vivactis Weber, Madrid, Spain; ^9^Department of Primary Care, Rochapea Healthcare Center, Navarra, Spain; ^10^Department of Pediatrics, Hospital Costa del Sol, Málaga, Spain; ^11^Public Health Institute of Navarra, Navarra, Spain; ^12^Department of Epidemiology, General Directorate of Public Health, Castilla y León, Spain; ^13^Department of Pediatrics, Nazaret Healthcare Center, Valencia, Spain; ^14^Department of Quantitative Methods in Economics and Management, University Las Palmas de Gran Canaria. Las Palmas, Spain; ^15^National Influenza Centre, Scientific Advisor and Emeritus Director, School of Medicine, University of Valladolid, Castilla y León, Spain.

**SUPPLEMENTARY FILE 1: PRE READ**

**Index**

[1. Description of the disease 3](#_Toc135393158)

[2. The MCDA 4](#_Toc135393159)

[2.1 Methodology 4](#_Toc135393160)

[2.2 An example 5](#_Toc135393161)

[2.3 MCDA in RSV 7](#_Toc135393162)

[3. Vaccinex framework 9](#_Toc135393163)

[References 12](#_Toc135393164)

# 1. Description of the disease

RSV is an ubiquitous *ortoneumovirus* of the family *pneumoviridae* [1,2] that produces a wide spectrum of respiratory symptoms. It has a seasonality that varies between regions, causing seasonal epidemics each year. [3]. In the Iberian Peninsula, historically, RSV has its highest incidence between November and March^[[1]](#footnote-1)^ [4].

RSV can be transmitted through airborne droplets, contaminated surfaces, or by direct contact with oral or nasal secretions [5]. The main routes of entry of the virus are the ocular conjunctiva and both nasal and oral mucosa. [6]. After initial infection, RSV replicates in the nasopharynx during an incubation period of 1 to 4 days and can be contagious from the third day of infection. [7] until the eighth day of infection. [7]. In severe cases, the host may be infectious for up to 4 weeks after infection. [7,8].

RSV infections occur in people of any age, causing mild and self-limited infections^[[2]](#footnote-2)^ , causing upper respiratory tract infection. However, there are age groups where the infection can incur in more severe cases, such as infants in the first year of life and people over 65 years of age. 90% of healthy infants will have been infected with RSV by the age of 2 years. [9,10].

Severe cases occur when the infection evolves and affects the lower respiratory tract, leading to pneumonia^[[3]](#footnote-3)^ , bronchopneumonia and bronchiolitis^[[4]](#footnote-4)^ . Among the elderly population with respiratory disease, RSV can cause an exacerbation of COPD or asthma, complicating these underlying conditions. [11].

Several risk factors associated with increased severity of infections with this virus have also been identified. These include physiological factors, such as prematurity^[[5]](#footnote-5)^ , low birth weight and height, children <6 years, congenital lung diseases (bronchopulmonary dysplasia, cystic fibrosis), cardiac or immune diseases, neuromuscular disorders, Down syndrome, as well as a family history of atopy and/or asthma; *environmental* factors, such as maternal smoking, indoor smoke pollution, etc.; and *social factors*, which include living with siblings or crowded living conditions, among others [12].

Although most cases of RSV in healthy infants are mild, the course of the disease is unpredictable. In other words, it is difficult to predict which infants will develop severe disease, and which will require hospitalization, oxygen support or other palliative care. This results in substantial clinical and economic burden (outpatient consultations, emergency care, hospitalizations, or even pediatric intensive care) and, in some cases, death. Although RSV deaths are infrequent in high-income industrialized countries (including Spain), they may occur in healthy infants without underlying pathologies or risk. [13-16].

# 2. The MCDA

## 2.1 Methodology

Methodologically, all MCDA contemplates the following common stages or principles:

1. **Problem definition**: identify the objectives, the type of decision, the alternatives, the members of the committee and the required results.
2. **Criteria selection**: identifying and structuring the value elements or criteria that are important for evaluating the alternatives in question.
3. **Measurement of alternatives**: gather the available evidence for each criterion.
4. **Weighting of the criteria**: to know the relative importance that each committee member gives to each criterion according to their individual preferences.
5. **Scoring of the alternatives**: to know the score that each member of the committee gives to the intervention evaluated in each criterion.
6. **Calculation of the total value of the alternatives**: aggregate the scores of the alternatives, weighted by the weights of the criteria, to obtain the total estimated value.
7. **Interpretation of results**: qualify and interpret the results of the MCDA, including a possible sensitivity analysis, to support decision making.

When determining the criteria that are relevant in decision making, there are two options: to create an ad hoc framework specific to that decision or to use a standard framework and adapt it to that situation. In either case, the criteria must meet certain **methodological requirements.** [17]:

1. **Completeness**: include all relevant components in the decision.
2. **No redundancy**: components must not be duplicated.
3. **Mutual independence**: the score of each component must be independent of the score of the other components.
4. **Operationalization**: each component must be unambiguously defined and have data on which to base the assessment.

## 2.2 An example

To facilitate the understanding of the MCDA methodology, we will give an example of its application in the daily life of a family that wants to buy a toaster for their home. Let's imagine that there are 2 types of toasters available on the market (toasters A and B). First, the family meets to decide what criteria they will consider when choosing the toaster.

| **First, the family decides that the criteria to consider when buying a toaster are:**   - Adjustable browning level - Uniformity of roasting - Number of slots - Price |
| --- |

After agreeing on these criteria, each family member decides how important each criterion is to him/her, weighting the criteria on a scale between 1 and 5 points, where 1 is not very important and 5 is very important. For example, for John, the father of the family, the most important criterion is the price, followed by the adjustable browning level, the evenness of browning and finally the number of slots in the toaster (**Table [S1].1**). This would apply to any toaster on the market, although it is the particular opinion of this family member.

**Table (S1).1. Weighting of the criteria for assessing the roasters in the market for Juan**

| Criteria | Weight |
| --- | --- |
| Adjustable browning level | 1  2  3  4  5 |
| Uniformity of roasting | 1  2  3  4  5 |
| Number of slots | 1  2  3  4  5 |
| Price | 1  2  3  4  5 |

The next step is to gather information (based on these criteria) about the toasters available on the market, either by searching for information online or in physical stores or by asking family and friends for their opinion.

Based on the information found by the family, each member evaluates each of the toasters in the market. Table (S2).2 summarizes the information found and how Juan scores it. For example, for Juan, toaster A is better in all aspects compared to toaster B (scores from 1 to 3), except for the price, a criterion that he scores with a -2 because it is more expensive than toaster B.

**Table (S1).2. Juan's score for roaster A versus B**

| Criteria | Evidence Synthesis (Toaster A vs. Toaster B) | Score |
| --- | --- | --- |
| Adjustable browning level | Toaster A: Available, but no data on the quality of its fit.  Toaster B: Not available | 3: Toaster A is much better than B  2  1  0: no difference  -1  -2  -3: Toaster A is much worse than B |
| Uniformity of roasting | Toaster A: Excellent (data sheet). Worsens after 1 year (friend)  Toaster B: Good (technical data sheet). | 3: Toaster A is much better than B  2  1  0: no difference  -1  -2  -3: Toaster A is much worse than B |
| Number of slots | Toaster A: 4  Toaster B: 1 | 3: Toaster A is much better than B  2  1  0: no difference  -1  -2  -3: Toaster A is much worse than B |
| Price | Toaster A: €55  Toaster B: €15 | 3: toaster A is much cheaper than toaster B  2  1  0: no difference  -1  -2  -3: Toaster A is much more expensive than B |

To estimate a final value, each member of the family makes a calculation taking into account the weighting they gave to each criterion and the relative score for Toaster A. For example, for John, the value for the level of toast is 0.19, since his score was 2, and his normalized weighting is 0.29 (4/14). The sum of each of these values results in 0.50, which divided by 3 (the maximum value for each scale), results in a final value of 0.17 (out of 1) for roaster A versus B. In other words, toaster A gives John a positive value relative to toaster B.

**Table (S1).3. Calculation of the final value of roaster A for Juan**

| Criteria | Weighting | Normalized weighting | Score | Final estimated value |
| --- | --- | --- | --- | --- |
| Adjustable browning level | 4 | 0,29 | 2 | 0,57 / 3* = 0,19 |
| Uniformity of roasting | 3 | 0,21 | 1 | 0,21 / 3 = 0,07 |
| Number of slots | 2 | 0,14 | 3 | 0,42 / 3 = 0,14 |
| Price | 5 | 0,36 | -2 | -0,71 / 3 = -0,24 |
| Total | 14 | 1,00 | - | 0,50 / 3 = 0,17 |

When each member of the family has done this exercise, they meet to discuss their arguments and decide which toaster to buy. Also, beyond the overall quantitative calculation, they can consider some contextual criteria, in case it affects their decision, such as the current availability in the store, free space on the countertop or possible shipping costs.

## 2.3 MCDA in RSV

The following describes the steps we will follow to conduct the MCDA for this project, focused on assessing the value of Nirsevimab, a new RSV preventive measure.

**Step 1: Choosing the relevant criteria**

Based on the available evidence on the pathology, the most appropriate ad hoc framework (or set of criteria) to assess the value of the intervention against the comparator will be decided, starting from a pre-established framework, as we will see in section 3.

**Step 2: Assignment of weights to each criterion**

Each expert, from his or her individual perspective, will assign a weight or weighting to each of the criteria selected in the previous step, according to the relative importance he or she gives to each of them. The weighting will be done on a scale between 1 and 5 points. It is important to note that the weight assigned to each criterion is independent of the intervention to be evaluated.

**Step 3. Search for evidence**

Vivactis Weber will synthesize the published information on the disease and on Nirsevimab into an evidence summary, which will be reviewed and validated by the clinicians on the project's expert committee.

**Step 4: Assigning a score to Nirsevimab**

Based on the available evidence and their own perception, each expert will assign a score to Nirsevimab vs. a comparator, for each of the criteria considered (decided at the 1st step). The score will be based on a certain scale that will depend on whether the criterion is absolute or relative to the comparator. In the first case (if it does not compare with respect to a comparator), the score will range from 0 to 5 points, with 0 being the lowest value and 5 the highest. For relative criteria (against the comparator), the scale will range from -5 to 5 points to reflect the full range of relative improvements or deteriorations. A value of 0 allows the exclusion of a criterion if it does not provide any value.

**Step 5: Calculation of the total estimated value of the intervention**

The total estimated value of each intervention will be obtained by linking the weighting of each criterion with the score given to each intervention in each criterion, through a linear additive model [18]:

Notes: V is the total estimated value, Vx the value contribution of the criterion x, Wx the weighting of the criterion x, ∑Wn the sum of all weights, and Sx the normalized score for each criterion (Sx = score / 5).

**Step 6: Qualitative discussion on the results**

In an online meeting, Vivactis Weber will present the results of the MCDA (weighting, scores, and final values) and a discussion will take place regarding the arguments and points of view applied by the experts for the evaluation of the intervention. This qualitative information is essential for the interpretation of the results.

**Step 7: Sensitivity analysis**

In order to assess the degree of replicability and internal validity of the analysis, one week after the previous meeting, an online re-test of the criteria weights and scores of the evaluated interventions will be conducted, asking the committee experts to re-weight and re-score. The degree of agreement or consistency between the responses made at the two points in time will be assessed.

**Step 8: Presentation of results**

The results of the MCDA analysis, as well as the methodological and deliberative considerations of the process, will be collected in a results analysis report, which will be drafted by the Vivactis Weber team and reviewed and validated by the members of the Expert Committee.

# 3. Vaccinex framework

Vaccines have some characteristics that are different from most drugs and health technologies, such as disease eradication capabilities and broader societal effects [19,20]. They should therefore be evaluated on the basis of broader criteria, such as their economic impact, contribution to disease eradication goals, caregiver issues, financial protection offered, equity or social acceptability. [21].

Therefore, this MCDA in VRS will be conducted through an ad hoc framework (set of criteria), starting from a pre-set value approach aimed at the evaluation of preventive measures. In this case, the starting framework will be that of Vaccinex, a model that is developed through a survey of a sample population (n=1000) in Flanders, Belgium, in which support for the use of these broader criteria to evaluate vaccines in funding decisions was investigated through a hypothetical vaccine called Vaccinex. The framework consists of a set of 40 possible criteria, divided into the two domains described below.[21]:

**Domain 1: Clinical aspects of vaccine and disease**

1. Mortality or lethality risk
2. Severity of symptoms
3. Duration of symptoms
4. Comorbidity risk
5. Transmissibility
6. Time to symptom development
7. Eradication potential
8. Availability of treatment
9. Prevention alternatives
10. Prevalence of the disease
11. Transmission mode
12. Impact on fertility
13. Vaccine efficacy or effectiveness
14. Certainty about the magnitude of the vaccine's effects
15. Mild adverse effects
16. Serious adverse effects
17. Herd immunity

**Domain 2: Socio-economic aspects of the vaccine and the disease**

1. Cost of the vaccine
2. Cost of illness (health system)
3. Cost of illness (patient)
4. Costs related to the production platform
5. Productivity costs: absenteeism
6. Productivity costs: attendance
7. Impact on caregivers
8. Impact on school activities
9. Generation of jobs for the country
10. Innovation stimulus
11. Image and goodwill (intangible effects)
12. Impact on health inequity
13. Public health awareness
14. Perception and fear
15. Resistance offered by anti-vaccination groups
16. Legal liability
17. Impact on socio-economically disadvantaged populations
18. Impact on the migrant population
19. Impact on the population of children
20. Impact on the elderly population
21. Impact on the LGBT population
22. Impact on the population of women
23. Impact on the population of pregnant women

# References

[1] Rima B, Collins P, Easton A, et al. ICTV Virus Taxonomy Profile: Pneumoviridae. J Gen Virol. 2017;98:2912-2913.

[2] Mejias A, Rodríguez-Fernández R, Oliva S, et al. The journey to a respiratory syncytial virus vaccine. Ann Allergy Asthma Immunol Off Publ Am Coll Allergy Asthma Immunol. 2020;125:36-46.

[3] Bloom-Feshbach K, Alonso WJ, Charu V, et al. Latitudinal variations in seasonal activity of influenza and respiratory syncytial virus (RSV): a global comparative review. PloS One. 2013;8:e54445.

[4] National Center for Epidemiology. Public Health Surveillance. Carlos III Health Institute. Ministry of Science and Innovation. Influenza, COVID-19 and other respiratory viruses [Internet]. 2021 [cited 2021 Apr 24]. Available from: https://www.isciii.es/QueHacemos/Servicios/VigilanciaSaludPublicaRENAVE/EnfermedadesTransmisibles/Paginas/Gripe.aspx.

[5] AEP. Respiratory syncytial virus (RSV) [Internet]. [cited 2021 Apr 19]. Available from: https://www.aeped.es/sites/default/files/documentos/vrs.pdf.

[6] Hall CB, Douglas RG. Modes of transmission of respiratory syncytial virus. J Pediatr. 1981;99:100-103.

[7] Carvajal JJ, Avellaneda AM, Salazar-Ardiles C, et al. Host Components Contributing to Respiratory Syncytial Virus Pathogenesis. Front Immunol. 2019;10:2152.

[8] Broberg EK, Waris M, Johansen K, et al. Seasonality and geographical spread of respiratory syncytial virus epidemics in 15 European countries, 2010 to 2016. Eurosurveillance. 2018;23:17-00284.

[9] Glezen WP, Taber LH, Frank AL, et al. Risk of primary infection and reinfection with respiratory syncytial virus. Am J Dis Child 1960. 1986;140:543-546.

[10] Ralston SL, Lieberthal AS, Meissner HC, et al. Clinical practice guideline: the diagnosis, management, and prevention of bronchiolitis. Pediatrics. 2014;134:e1474-1502.

[11] Openshaw PJM, Chiu C, Culley FJ, et al. Protective and Harmful Immunity to RSV Infection. Annu Rev Immunol. 2017;35:501-532.

[12] Piedimonte G, Perez MK. Respiratory Syncytial Virus Infection and Bronchiolitis. Pediatr Rev. 2014;35:519-530.

[13] Shi T, McAllister DA, O'Brien KL, et al. Global, regional, and national disease burden estimates of acute lower respiratory infections due to respiratory syncytial virus in young children in 2015: a systematic review and modelling study. The Lancet. 2017;390:946-958.

[14] Fauroux B, Simões EAF, Checchia PA, et al. The Burden and Long-term Respiratory Morbidity Associated with Respiratory Syncytial Virus Infection in Early Childhood. Infect Dis Ther. 2017;6:173-197.

[15] Ledbetter J, Brannman L, Wade SW, et al. Healthcare resource utilization and costs in the 12 months following hospitalization for respiratory syncytial virus or unspecified bronchiolitis among infants. J Med Econ. 2020;23:139-147.

[16] Palmer L, Hall CB, Katkin JP, et al. Healthcare costs within a year of respiratory syncytial virus among Medicaid infants. Pediatr Pulmonol. 2010;45:772-781.

[17] Thokala P, Devlin N, Marsh K, et al. Multiple Criteria Decision Analysis for Health Care Decision Making-An Introduction: Report 1 of the ISPOR MCDA Emerging Good Practices Task Force. Value Health. 2016;19:1-13.

[18] Goetghebeur MM, Wagner M, Khoury H, et al. Evidence and Value: Impact on DEcisionMaking - the EVIDEM framework and potential applications. BMC Health Serv Res [Internet]. 2008 [cited 2017 Feb 14];8. Available from: http://bmchealthservres.biomedcentral.com/articles/10.1186/1472-6963-8-270.

[19] Bell E, Neri M, Steuten L. Towards a Broader Assessment of Value in Vaccines: The BRAVE Way Forward. Appl Health Econ Health Policy. 2022;20:105-117.

[20] Knobler S, Bok K, Gellin B. Informing vaccine decision-making: A strategic multi-attribute ranking tool for vaccines-SMART Vaccines 2.0. Vaccine. 2017;35 Suppl 1:A43-A45.

[21] Luyten J, Kessels R, Vandermeulen C, et al. Value Frameworks for Vaccines: Which Dimensions Are Most Relevant? Vaccines. 2020;8:E628.

[22] McCormick BJJ, Waiswa P, Nalwadda C, et al. SMART Vaccines 2.0 decision-support platform: a tool to facilitate and promote priority setting for sustainable vaccination in resource-limited settings. BMJ Glob Health. 2020;5:e003587.

[23] Suwantika AA, Purwadi FV, Zakiyah N, et al. Multi-criteria decision analysis to prioritize the introduction of new vaccines in Indonesia by using the framework of the strategic multi-attribute ranking tool for vaccines (SMART vaccines). Expert Rev Vaccines. 2021;20:83-91.

[24] The National Academies of Engineering Medicine. Ranking Vaccines: A Prioritization Software Tool: Phase II: Prototype of a Decision-Support System [Internet]. 2013 [cited 2022 Mar 24]. Available from: https://www.nap.edu/read/13531/chapter/2.

1. In the last two years, marked by the COVID-19 pandemic, there have been more RSV cases outside the historically contemplated seasons (November-April), although it is not known whether this pattern will continue in the medium/long term. [↑](#footnote-ref-1)
2. in which the patient himself recovers after a few days. [↑](#footnote-ref-2)
3. inflammation of the alveoli [↑](#footnote-ref-3)
4. occlusion of the smallest airways [↑](#footnote-ref-4)
5. ≤35 weeks gestational age (WGA) [↑](#footnote-ref-5)
