# Supplementary material for: Evaluation of antibody-based preventive alternatives for respiratory syncytial virus: a novel multi-criteria decision analysis framework and assessment of nirsevimab in Spain
Source: BMC Infect Dis. 2024 Jan 18;24:99. doi: 10.1186/s12879-024-08988-9 (PMC10797756; doi:10.1186/s12879-024-08988-9)
Supplement: Supplementary file 2 — Supplementary Material 2: Evidence summary [file 12879_2024_8988_MOESM2_ESM.docx]

**Evaluation of Antibody-based Preventive Alternatives for Respiratory Syncytial Virus: A Novel Multi-Criteria Decision Analysis Framework and Assessment of Nirsevimab in Spain**

**Authors**: Jorge Mestre-Ferrándiz^1^, Agustín Rivero^2^, Alejandro Orrico-Sánchez^3,4,5^, Álvaro Hidalgo^6,7^, Fernando Abdalla^8^, Isabel Martín^9^, Javier Álvarez^10^, Manuel García-Cenoz^11^, Maria del Carmen Pacheco^12^, María Garcés-Sánchez^13^, Néboa Zozaya^8,14^, Raúl Ortiz-de-Lejarazu^15^

**Affiliations**: ^1^Department of Economics, University Carlos III, Madrid, Spain; ^2^Department of Management, Bioregión de Salud y Bienestar (BioMad), Madrid, Spain; ^3^Department of Vaccines Research, Fundación Para el Fomento de la Investigación Sanitaria y Biomédica de la Comunitat Valenciana (Fisabio), Valencia, Spain; ^4^Catholic University of Valencia, Spain; ^5^Centro de Investigación Biomédica en Red de Epidemiología y Salud Pública (CIBERESP); ^6^Weber Foundation, Madrid, Spain; ^7^Department of Economic Analysis and Finances, University of Castilla-La Mancha. Toledo, Spain; ^8^Department of Health Affairs and Policy Research, Vivactis Weber, Madrid, Spain; ^9^Department of Primary Care, Rochapea Healthcare Center, Navarra, Spain; ^10^Department of Pediatrics, Hospital Costa del Sol, Málaga, Spain; ^11^Public Health Institute of Navarra, Navarra, Spain; ^12^Department of Epidemiology, General Directorate of Public Health, Castilla y León, Spain; ^13^Department of Pediatrics, Nazaret Healthcare Center, Valencia, Spain; ^14^Department of Quantitative Methods in Economics and Management, University Las Palmas de Gran Canaria. Las Palmas, Spain; ^15^National Influenza Centre, Scientific Advisor and Emeritus Director, School of Medicine, University of Valladolid, Castilla y León, Spain.

**SUPPLEMENTARY FILE 2: EVIDENCE SUMMARY**

**Index**

[Introduction 3](#_Toc135393128)

[Objectives 3](#_Toc135393129)

[Scoring of the criteria 3](#_Toc135393130)

[Comparator 3](#_Toc135393131)

[Description of the drug 4](#_Toc135393132)

[Severity of disease 4](#_Toc135393133)

[1. Severity of symptoms 4](#_Toc135393134)

[2. Lethality risk 5](#_Toc135393135)

[3. Comorbidity risk 5](#_Toc135393136)

[Burden of disease 6](#_Toc135393137)

[4. Incidence of RSV cases 6](#_Toc135393138)

[5. Incidence on the outpatient setting 6](#_Toc135393139)

[6. Incidence on the inpatient setting 7](#_Toc135393140)

[7. Time of duration of acute symptoms 7](#_Toc135393141)

[Prevention or treatment alternatives 8](#_Toc135393142)

[8. Prevention Alternatives 8](#_Toc135393143)

[9. Availability of treatment 8](#_Toc135393144)

[Size of population 9](#_Toc135393145)

[10. Population in which the prevention strategy would be indicated 9](#_Toc135393146)

[Efficacy 9](#_Toc135393147)

[11. Efficacy of the preventive measure 9](#_Toc135393148)

[Population protection 11](#_Toc135393149)

[12. Group immunity (collective protection) 11](#_Toc135393150)

[13. Transmissibility 12](#_Toc135393151)

[Safety 12](#_Toc135393152)

[14. Serious adverse events 12](#_Toc135393153)

[15. Mild adverse events 13](#_Toc135393154)

[Quality of evidence 13](#_Toc135393155)

[16. Certainty about the efficacy of the preventive measure 13](#_Toc135393156)

[Impact on quality of life 14](#_Toc135393157)

[17. Impact on the population of children 14](#_Toc135393158)

[18. Impact on the population over 65 years of age 15](#_Toc135393159)

[19. Impact on caregivers 16](#_Toc135393160)

[Acquisition cost 16](#_Toc135393161)

[20. Monetary cost of the preventive measure 16](#_Toc135393162)

[Impact on other costs (direct and indirect) 17](#_Toc135393163)

[21. Cost of the disease on the health system (excludes acquisition cost) 17](#_Toc135393164)

[22. Productivity cost: absenteeism 18](#_Toc135393165)

[23. Cost of the disease on the patient (out-of-pocket expenses) 18](#_Toc135393166)

[Social benefits 19](#_Toc135393167)

[24. Impact on health inequity 19](#_Toc135393168)

[25. Public health awareness (including antibiotic resistance) 19](#_Toc135393169)

[26. Innovation stimulus 20](#_Toc135393170)

[List of abbreviations 22](#_Toc135393171)

[References 23](#_Toc135393172)

# Introduction

## Objectives

*Objectives of this MCDA*

The central objective of this research was to develop a novel MCDA framework for RSV preventive alternatives and to assess the value of nirsevimab *vs.* placebo as a systematic immunization approach to prevent RSV in neonates and infants during their first RSV season in Spain.

*Objectives of this document*

In this document, we present the available evidence related to the 26 criteria chosen for the framework of this MCDA. This evidence should serve as a reference for the scoring of the criteria. For each criterion, we will present 1) its definition 2) the type of criterion (whether it is absolute or relative) and 3) the available evidence.

## Scoring of the criteria

Each expert will assign each of the 26 chosen criteria an individual and confidential score. For the **absolute criteria** (those that do not include comparisons between preventive measures), the score will range from 0 to 5, with 0 being the lowest and 5 being the highest. For the **relative criteria** (those comparing with the alternative), the score will range from -5 to 5 to reflect the full range of comparative effects (improvements or deteriorations with respect to the alternative). To do the scoring, experts should use the **Excel document** sent by Vivactis Weber, along with this summary of evidence.

## Comparator

This MCDA will compare nirsevimab with placebo (no intervention). In the relative criteria, we will present the evidence for this comparison.

## Description of the drug

*Nirsevimab*

Nirsevimab is a recombinant human IgG1 kappa monoclonal antibody with structural modifications to give it a longer half-life that binds to the F1 and F2 subunits of the RSV fusion (F) protein (antigenic site 0 of the protein in its pre-F form), to block viral entry into the host cell[1]. It is designed for use in a broad pediatric population, in neonates and infants during their first RSV season. It is not expected to be for hospital use[2].

It is administered intramuscularly in a single dose of 50mg for neonates and infants weighing less than 5kg, and 100mg for neonates and infants weighing 5kgs or more. In addition, it offers protection for at least 5 months[1].

On September 15, 2022, the Advisory Committee for the Evaluation of Medicinal Products for Human Use (CHMP) through an accelerated assessment process[2] gave a positive opinion for marketing authorization[3]. Authorization by the EMA and the European Commission is expected in October 2022.

# Severity of disease

## Severity of symptoms

*[Criterion definition***]**: Severity of symptoms in RSV-infected people.

*[Type of criterion***]**: Absolute

**[***Evidence available***].**

In most cases, RSV causes mild, self-limited infections^[[1]](#footnote-2)^ of the upper respiratory tract, the main symptoms of which are sneezing, nasal congestion, runny nose, dry cough, otitis media, fever and/or chills, difficulty sleeping, decreased appetite, irritability, and lethargy[4–6].

However, in some cases, this infection evolves into a more severe infection affecting the lower respiratory tract, leading to pneumonia^[[2]](#footnote-3)^, bronchopneumonia and bronchiolitis^[[3]](#footnote-4)^, causing wheezing, shortness of breath, cough, tachypnea^[[4]](#footnote-5)^ , cyanosis^[[5]](#footnote-6)^ , hypoxemia^[[6]](#footnote-7)^ and occasionally apnea.[4–6].

## Lethality risk

*[Criterion definition***]**: Rate of deaths per number of cases of RSV infection.

*[Type of criterion***]**: Absolute

**[**Evidence available**].**

According to a Spanish study calculating the rate of deaths per number of hospitalization cases in children <2 years, 82 deaths were recorded in 100,115 children <2 years hospitalized for RSV over a 6-year period (14 deaths each year), representing a death rate of 82 children per 100,000 children <2 years hospitalized [8].

## Comorbidity risk

*[Criterion definition***]**: Likelihood that RSV-infected people will develop other associated diseases in the short, medium, or long term.

*[Type of criterion***]**: Absolute

**[***Evidence available***].**

*Short-term complications:* short-term complications of RSV infection include acute respiratory distress, wheezing, and acute otitis media.[9–11]. In addition, there is an association between RSV infection early in life and the development of recurrent **wheezing.** Children ≤12 months with RSV infection have significantly higher rates of wheezing than children without RSV infection (62% *vs.* 32% at 11 months).[12]. **Acute otitis media** is a very common complication of RSV infection, having been observed in 58% of children <3 years with RSV infection during the 2000-2002 seasons. [13]. In addition, RSV is the cause of acute otitis media in more than 50% of children <5 years. However, RSV causes otitis more frequently in children <2 years (70%) than in older children (30%).[9].

*Medium and long-term complications:* the percentage of children who have presented clinical signs compatible with RSV infection up to the age of 6 years (RSV<6) and require **asthma** medications is significantly higher than uninfected children: bronchodilators (62% *vs.* 42%, p<0.001), leukotriene receptor antagonists (16% *vs.* 6%, p=0.002), inhaled steroids (26% *vs.* 16%, p=0.009); oral steroids (18% vs*.* 11%, p=0.023)[14]. In addition, the incidence of **allergic rhinitis** is significantly higher in RSV-infected vs. non-infected children: 3 years: 6.5% *vs.* 1.7%, p=0.011; 4 years: 7.3% *vs.* 3.1%, p<0.05); these rates are similar from 5 years of age onward.[14]. Children hospitalized for RSV also have a higher incidence of recurrent **wheezing** of some severity up to 6 years of age (46.7% vs. 27.4%; p = 0.001).[14–16]. Acute lower respiratory tract infections (ALRTI) due to RSV is associated with reduced **lung function** during school age, which may extend into adulthood, resulting in reduced quality of life.[17]. Thus, in a 20-year prospective follow-up study in Finland, at least one abnormal lung function outcome was observed in 44% of subjects who developed RSV ALRTI in the first 2 years of life, compared to 31% of controls (P=0.05). In addition, RSV ALRTI in infancy was an independent risk factor for lung function abnormalities [spirometry airway function; OR, 5.27; 95% CI, 1.60-17.36)].[18]

# Burden of disease

## Incidence of RSV cases

*[Criterion definition***]**: Proportion of the population infected with RSV.

*[Type of criterion***]**: Absolute

**[***Evidence available***].**

Approximately 65% of children ≤12 months[19] and 90% of children <2 years [19,20] become infected with RSV, showing that most children are at risk for the disease. In addition, because RSV infection is centered in the first months of life, when the child's immune system is not fully mature, there is a risk for multiple RSV infections in the first few years of life[21].

## Incidence on the outpatient setting

*[Criterion definition***]**: Incidence rates for RSV infection on the outpatient setting (primary and specialty care visits).

*[Type of criterion***]**: Absolute

**[***Evidence available***].**

According to the results of the longitudinal study [22] BARI, in Spain, the annual incidence rate of events requiring healthcare in **primary care** for RSV in children ≤12 months in the 2017-2018 season was 39,690 per 100,000 children^[[7]](#footnote-8)^ . For **specialized care**, this rate was 882 per 100,000 children^[[8]](#footnote-9)^ .

## Incidence on the inpatient setting

*[Criterion definition***]**: Incidence rates for RSV infection on the outpatient setting (ED visits and hospitalizations).

*[Type of criterion***]**: Absolute

**[***Evidence available***].**

According to the results of the longitudinal BARI study[22], the annual incidence rate of events requiring **emergency department visits** for RSV in children ≤12 months in the 2017-2018 season was 10,584 per 100,000 children, as defined by RSV + acute bronchiolitis, while the incidence rate of **hospitalizations**^[[9]](#footnote-10)^ was 2,520 per 100,000 children <2 years.

It is estimated that four out of every 100 children up to 12 months and two out of every 100 children up to 24 months are hospitalized in our country for bronchiolitis. The hospitalization rate is 15 times higher in the first year of life than in the second year of life. [8]. It should also be noted that 88% of hospitalizations for RSV in Spain are observed in the months between November and March, and that 95.1% of hospitalizations in children <2 years occur in healthy children born at term and only 4.9% in children with risk factors.[24].

Moreover, RSV is responsible for 62% of viral pneumonias requiring hospitalization in Spain in children under 12 months of age, being the main cause, with a great difference over the rest of viruses[25]. In addition, RSV causes 16 times more hospitalizations than influenza in infants <1 year old.[26,p.1993–2008].

## Time of duration of acute symptoms

*[Criterion definition***]**: Time of duration of acute symptoms in RSV-infected persons.

*[Type of criterion***]**: Absolute

**[***Evidence available***].**

Symptoms take two to eight days to appear from the time a person is exposed to RSV. Symptoms usually last three to seven days. Most children and adults recover completely within one to two weeks[27]. The average length of stay for children requiring hospitalization for RSV is between 5.4 and 5.7 for children <2 years of age.[23,p.2015–2018].

# Prevention or treatment alternatives

## Prevention Alternatives

*[Criterion definition***]**: Existence, availability, effectiveness, and safety of RSV prevention alternatives.

*[Type of criterion***]**: Absolute

**[***Evidence available***].**

Currently, there is no long-acting pharmacological preventive measure for RSV in in neonates and infants during their first RSV season. The only available option is the monoclonal antibody palivizumab, indicated for the prevention of severe lower respiratory tract disease requiring hospitalization caused by RSV in children at high risk of disease^[[10]](#footnote-11)^ . The efficacy and safety of palivizumab has been evaluated in 3 pivotal randomized clinical trials versus placebo, as well as in several observational studies in actual clinical practice. The results of the pivotal trials demonstrated efficacy of palivizumab versus placebo only in high-risk groups. In addition, studies in real clinical practice demonstrated that monthly injections^[[11]](#footnote-12)^ may lead to poor adherence and consequently decrease its effectiveness.[30,31]. Palivizumab has not been shown to reduce hospital stay, oxygen or mechanical ventilation requirements, or mortality and has a cost of more than €4,000/child, requiring 4-5 doses to cover the season based on the reported price.[28,32]. On the other hand, the Spanish Association of Pediatrics and the Spanish Society of Pediatric Pneumology consider its administration controversial, indicating that there is no unanimity as to its indications for use[33].

## Availability of treatment

*[Criterion definition***]**: Existence, availability, effectiveness, and safety of treatment alternatives for RSV-infected people.

*[Type of criterion***]**: Absolute

**[***Evidence available***].**

Currently, there is no effective antiviral treatment for RSV infection. Despite the existence of an antiviral treatment (ribavirin), it can only be administered in severe cases, and its routine use is not recommended due to a lack of solid evidence on its efficacy and safety profile [36,37]. In Spain, it is only used for hospital treatment of severe RSV infection in high-risk patients^[[12]](#footnote-13)^ and its administration is complex. Therefore, the treatment of RSV infection is limited to maintaining adequate hydration and nutrition[20] and, where necessary, administering additional oxygen or mechanical ventilation.[20,33,34]. In severe cases, blood transfusion, tube feeding, dialysis or cardiac catheterization may also be required.[35].

# Size of population

## Population in which the prevention strategy would be indicated

*[Criterion definition***]**: Proportion of the population in which the prevention strategy would be indicated.

**[***Type of criterion***]**: Relative

**[***Evidence available***].**

Nirsevimab is the first investigational long-acting antibody designed to provide protection against RSV for in neonates and infants during their first RSV season [2].

# Efficacy

## Efficacy of the preventive measure

*[Criterion definition***]**: Level of efficacy of the RSV preventive measure.

**[***Type of criterion***]**: Relative

*[Evidence available for nirsevimab vs placebo***.]**

For this section, we present data from the MELODY trial (phase 3), as well as a combined, pre-specified per protocol analysis of the MELODY and phase 2b trial data.

**MELODY (phase 3)**

The MELODY trial (NCT03979313[36,p.03979313]) evaluated the efficacy and safety of nirsevimab versus placebo in infants born with at least 35 weeks gestational age and who were in their first RSV season at the time of the trial.[1].

Children were excluded if they met criteria for receiving palivizumab, had fever or acute illness 7 days prior to randomization, had active RSV infection, or had received any prior preventive measures (palivizumab, RSV vaccine or monoclonal antibody, maternal vaccine)[1].

This was a randomized (2:1 ratio nirsevimab 50mg for <5kg or 100mg for ≥5kg/placebo), phase 3, double-blind (investigators/parents or guardians) study. A total of 1,490 infants (994 nirsevimab/496 placebo) were included, of whom 58% were up to 3 months old, 32% between 3 and 6 months and the remaining (10%) were older than 6 months (median age: 2.6 months). Eighty-seven percent were born at ≥37 weeks gestation, and 60% were over 5kg. [1].

The primary efficacy endpoint was medically attended RSV-associated lower respiratory tract infection^[[13]](#footnote-14)^ up to 150 days after injection. Secondary endpoints were hospitalization for RSV-associated lower respiratory tract infection up to 150 days after injection: incidence of RSV hospitalization, safety, and pharmacokinetics/anti-drug antibodies. As an exploratory variable, the investigators included the number of treatments required to prevent one case of RSV-associated lower respiratory tract infection[1].

The main conclusion of the study was that a single injection of nirsevimab given before the RSV season protected both preterm and term infants from medically attended RSV-associated lower respiratory tract infection. Details of the results are shown below[1]:

- **Medically attended** RSV-associated lower respiratory tract infection occurred in 12 infants (1.2%) in the nirsevimab group and in 25 infants (5.0%) in the placebo group; these results correspond to an efficacy of 74.5% (95% confidence interval (CI), 49.6 to 87.1; P<0.001) for nirsevimab.
- Infants who received nirsevimab had a lower risk of **medically attended** RSV-associated lower respiratory tract infection than those who received placebo (risk ratio, 0.23; 95% CI, 0.12 to 0.47).
- **Hospitalization** for RSV-associated lower respiratory tract infection occurred in 6 infants (0.6%) in the nirsevimab group and 8 infants (1.6%) in the placebo group (efficacy, 62.1%; 95% CI, -8.6 to 86.8; P = .07).
- Per 1,000 infants treated:
  - 93.6 cases (95% CI 63.0 to 124.0) of **medically treated** RSV-associated lower respiratory tract infection were averted. In other words, for every 11 (9-16) children treated, one case is averted.
  - 17.7 cases (95% CI, 2.0-33.0) of **hospitalization** for RSV-associated lower respiratory tract infection were avoided. In other words, for every 57 (31-500) children treated, one hospitalization is avoided.

**Pooled analysis (phase 2b and MELODY)**

In the pooled analysis[37], a total of 2,350 children were included (860 healthy, preterm, and under 5 kg children in the phase IIb trial and all (n=1,490) in the MELODY trial)[38]. Their results reinforce the efficacy data from the previous trials, increasing the certainty about its effects, especially in the aspect related to hospitalization. The main results are detailed below[37]:

- Efficacy of 79.5% (95% CI 65.9%-87.7%) *vs.* placebo in preventing medically attended RSV-associated lower respiratory tract infection.
- Efficacy of 86.0% (95% CI 62.5%-94.8%) *vs.* placebo in preventing very severe cases of medically attended RSV-associated lower respiratory tract infection.

Efficacy of 77.3% (95% CI 50.3%-89.7%) *versus* placebo in preventing hospitalizations for RSV-associated lower respiratory tract infection.

# Population protection

## Group immunity (collective protection)

*[Criterion definition***]**: Implementation of the preventive measure in the majority of the population also indirectly protects those who do not receive the preventive measure (as the risk of infection decreases).

*[Type of criterion***]**: Absolute

**[***Evidence available***].**

Collective protection is related to two elements:

- Sterilizing immunity that prevents replication of the virus in the host and blocks the release of viral load to infect others.
- Prolonged and sufficient duration of sterilizing immune protection (>10 years or lifetime).

There is no specific evidence regarding this criterion. Therefore, when analyzing this aspect, the possible partial collective protection that could be generated through the use of nirsevimab (considering the target population) should be considered.

## Transmissibility

*[Criterion definition***]**: Number of cases of infection occurring from a primary RSV-infected case, expressed through the basic reproductive number.

*[Type of criterion***]**: Absolute

**[***Evidence available***].**

The basic reproductive number for this disease (R_0_ ) is 4.5 (range: 1.7 - 8.2), according to a systematic review using data from several countries[39]. On the other hand, children <5 years of age are an important factor in the spread of infection, due to the effect of virus transmission at the population level. A study conducted in the United States that developed a model of RSV transmission showed that the risk of infecting other individuals decreases with age. Thus, while a child under 5 years of age can transmit RSV to 1.53-1.57 individuals, patients aged 5-24 years, 25-49 years and ≥50 years can infect 0.98-1.21, 0.65-0.77 and 0.45-0.55 others, respectively.[40]. Based on the predictions of this model and although the goal of a medical intervention may not be the benefit of third parties, a beneficial consequence of prophylaxis of children aged 1-5 years could be effective in decreasing infection in adults ≥50 years than prophylaxis of these individuals themselves[40].

# Safety

## Serious adverse events

*[Criterion definition***]**: Serious adverse events occurring in people receiving the preventive measure.

**[***Type of criterion***]**: Relative

**[***Available evidence nirsevimab vs placebo***].**

Nirsevimab showed good tolerability and no safety concerns in its Phase 1A, 1b/2A, 2b, and MELODY/MEDLEY studies. Conclusions from the MELODY trial indicates that no clinically meaningful differences in safety outcomes were observed between the nirsevimab and placebo groups. Reported **serious adverse events** were very similar in both groups (6.8% nirsevimab *vs.* 7.3% placebo)[1]. Reported deaths were not related to nirsevimab or placebo[1].

## Mild adverse events

*[Criterion definition***]**: Mild adverse events occurring in people receiving the preventive measure.

**[***Type of criterion***]**: Relative

**[***Available evidence nirsevimab vs placebo***].**

Except for local injection site reactions, no indications, or risks about the safety of nirsevimab have been shown. 87.4% (n=863) of nirsevimab patients and 86.8% (n=426) of placebo patients had any type of adverse event. Of these, only 1% (nirsevimab) and 1.4% (placebo) were considered to be related to the preventive measure.[1]. The most frequent adverse events (>10% of children) were all grade 1 and 2 (mild or moderate), very similar between both groups and consisted of: upper respiratory tract infections (420 [42.5%] nirsevimab *vs.* 210 [43.7%] placebo), pyrexia (140 [14.1%] *vs.* 63 [12.8%], nasal congestion (120 [12.1%] *vs.* 68 [13.1%]) and dentition-related events (111 [11.2%] *vs.* 53 [10.9%])[1].

# Quality of evidence

## Certainty about the efficacy of the preventive measure

*[Criterion definition***]**: Level of certainty about the efficacy of the preventive measure.

*[Type of criterion***]**: Absolute

For this aspect, the quality of the trial (randomized trials are the gold standard), the p-value (the lower the p-value, the more significant the results, and in general, a p<0.05 is considered significant) and the confidence interval (which should not include the value "0" to be significant) should be considered.

**[***Evidence available***].**

The MEDI8897 phase 2b clinical trial [38] (NCT02878330[41]) and the MELODY trial are two studies evaluating the efficacy of **nirsevimab** compared to placebo. Both are randomized, double-blind, conducted on a total of 1,453 and 1,490 children, respectively. In 164 and 160 centers located in 23 and 21 countries, respectively. Both trials were conducted in accordance with the Good Clinical Practices (GCP) guidelines of the International Conference on Harmonization of Technical Requirements for Pharmaceuticals for Human Use (ICH) and the Declaration of Helsinki[1] [38].

In the combined test, these were the results obtained.[37]:

- Medically attended RSV-associated lower respiratory tract infection (primary): efficacy 79.5% (95% CI, 65.9%-87.7%, p<0.0001) *vs.* placebo.
- Medically attended RSV-associated lower respiratory tract infection (very severe cases) (primary): 86.0% efficacy (95% CI, 62.5%-94.8%, p<0.0001).
- Hospitalization for RSV-associated lower respiratory tract infection (secondary): efficacy 77.3% (95% CI, 50.3%-89.7%, p=0.0002) *vs.* placebo.

# Impact on quality of life

## Impact on the population of children

*[Criterion definition***]**: Level of impairment (stress, quality of life) produced in RSV-infected children.

*[Type of criterion***]**: Absolute

**[***Evidence available***].**

RSV affects the quality of life of children who contract RSV infection. In Spain, the loss of quality of life since disease diagnosis in children ≤12 months affected by RSV is 39%, 29%, and 8% at 0, 7, and 14 days, respectively[42]. The quality of life of children with RSV at diagnosis and in the 7 days thereafter (060-0.71 out of 1) is below that of the general population >65 years (0.77) or patients with chronic diseases, such as diabetes mellitus (0.69) or chronic obstructive pulmonary disease (0.61)[42–44].

The stress level of children with RSV infection^[[14]](#footnote-15)^ is high and persists for at least one month. The child's stress level is 4.8 (out of 7) after hospital discharge and 2.1 one month later.[45]. In addition, hospitalizations for RSV during the first year of life have a significant impact on the long-term deterioration of the quality of life of hospitalized children. In Spain, a study has evaluated the long-term impact of RSV hospitalizations on the quality of life of children ≤12 months during the 6 years after hospitalization, showing a significant reduction in these patients compared to healthy controls from the second year to the sixth year after RSV hospitalization (between -3% and -5%; p=0.001).[14].

## Impact on the population over 65 years of age

*[Criterion definition***]**: Level of impairment (stress, quality of life) produced in RSV-infected people over 65 years of age.

*[Type of criterion***]**: Absolute

**[***Evidence available***].**

The RSV MCDA framework evaluating nirsevimab versus placebo has a focus on neonates and infants during their first RSV season (nirsevimab target population). However, the experts' choice of framework also considered indirect RSV prevention effects^[[15]](#footnote-16)^ in other non-pediatric populations in the evaluation. As seen in the section on transmissibility, prophylaxis in children aged between 1 and 5 years could be more effective in preventing infection in adults ≥50 years than prophylaxis in these individuals, provided that viral replication in mucous membranes is avoided with treatment. In other words, a preventive measure that reduces the incidence of RSV infections in children ≤12 months could also indirectly benefit those over 65 years of age and improve their quality of life. In the following, we describe the evidence on the impact on the population over 65 years of age.

On the one hand, RSV infections in the elderly are comparable in their clinical profile and cause of severe illness or death to influenza infections, although with a lower incidence than influenza.[46–48].

On the other hand, a North American study conducted on people over 60 years of age showed that RSV infection reduces the quality of life of these people by 11% (0.896 [0.854-0.953] vs. 0.801 [0.712-0.937]) from pre-season to one week after symptom onset[49]. Most of these patients develop signs of upper respiratory tract infection, such as nasal congestion and rhinorrhea (22-78%) or sore throat (16-64%) three to five days after infection. Other non-specific symptoms such as asthenia, anorexia, and fever (48-56%) may also occur with varying severity. As the virus progresses and affects the lower respiratory tract, symptoms such as cough (85-95%), wheezing (33-90%) and dyspnea (51-93%) may appear.[50].

## Impact on caregivers

*[Criterion definition***]**: Number of hours spent by caregivers with RSV-infected people, and level of impairment (stress, quality of life) produced in those caregivers.

*[Type of criterion***]**: Absolute

**[***Evidence available***].**

A US study of children ≤12 months hospitalized for RSV showed that caregivers of preterm infants had to spend, on average, 282 hours for hospital visits vs. 140 hours for caregivers of children born at term[51]. Caregivers of children hospitalized with RSV had high levels of stress, anxiety and worsening health status, as well as disruption of their family routines.[52,53]. The high level of caregivers' stress persists for at least one month, as well as the deterioration of their family activities.[45,52,53]. According to the study by Pokrzywinky et al. (2019), the stress level of parents of children ≤12 months hospitalized for RSV was 83% at hospital discharge (5.8 on a scale of 7) and 34% one month later (2.4 on a scale of 7) [45]. In addition, impairment of daily activities^[[16]](#footnote-17)^ affected 79% and 74% of mothers and fathers of children hospitalized for RSV at discharge and 24% and 18% one month after discharge, respectively[45].

# Acquisition cost

## Monetary cost of the preventive measure

*[Definition of the criterion***]**: Acquisition cost of the preventive measure, in monetary terms.

**[***Type of criterion***]**: Relative

**[***Evidence available***].**

Nirsevimab is not yet authorized in Europe and the United States, therefore, it is not marketed in Spain (or in any other country), so its acquisition price is still unknown. Nirsevimab is intended for the protection of neonates and infants during their first RSV season. For this MCDA, we will assume a purchase price in line with the cost of immunization of other innovative vaccines. In addition, we assume a cost for its administration of 6,53€ (1 dose[54,55]). The target population of the strategy is 341,315 children (children born in Spain in 2020[56]).

The cost of placebo is 0€. Its cost of administration is €6.53 (1 dose[54,55]).

# Impact on other costs (direct and indirect)

## Cost of the disease on the health system (excludes acquisition cost)

*[Criterion definition***]**: Costs avoided by the use of the preventive measure, in relation to the use of health resources (primary care, specialized care, emergencies and hospitalizations) of people infected with RSV, excluding the cost of acquiring the measure.

**[***Type of criterion***]**: Relative

**[***Evidence available***].**

In a study conducted in Spain, which estimates the impact of the use of nirsevimab *versus* usual clinical practice (**placebo or palivizumab depending on the population**) on the costs for the NHS, a static analytical decision model is performed, which considers the entire cohort of children in Spain, during their first season in RSV. Considering an efficacy of 79.5%, savings of 29.8 million euros for the NHS in one season are estimated. In this study, the acquisition costs of the drugs are not considered.[57].

In another study comparing the impact of using **nirsevimab *versus* placebo** on costs to the health care system in the United States, a static Markov model is performed, finding that, with 76% coverage of the population of children (and 70% efficacy), using nirsevimab would save 50% of total health care costs ($1.6 billion *versus* $800 million), through reductions in outpatient and inpatient incidences caused by RSV[58].

The annual direct healthcare cost of all children ≤12 months requiring medical care for RSV in Spain is between 31.8 and 49.1 million € (M€) depending on the definition used to determine RSV cases^[[17]](#footnote-18)^ . This cost represents 78% and 83% of the total direct health care cost for children <5 years, respectively, and is mainly due to the higher cost of hospitalization (22.5 M€ *vs.* 33.9 M€).[59,60].

## Productivity cost: absenteeism

*[Definition of the criterion***]**: Indirect costs avoided to the system due to caregiver absenteeism as a result of using the preventive measure.

**[***Type of criterion***]**: Relative

**[***Evidence available]*

No specific evidence is available for this criterion. Therefore, it must be assumed to what extent the relative improvement in health produced by nirsevimab versus placebo could be reflected in a lower level of work absenteeism.

What is documented is that, according to a US study, the overall work productivity loss, absenteeism and presenteeism of mothers of infants ≤12 months preterm^[[18]](#footnote-19)^ hospitalized for RSV infection was 91% (total productivity), 73% (absenteeism) and 64% (presenteeism) at hospital discharge and 31% (total productivity), 16% (absenteeism) and 23% (presenteeism) at one month of hospital discharge[45]. In Spain, it has been estimated that indirect costs account for approximately 15.9% of total costs. In other countries, these costs would range from 5.9% to 31.9% of total costs.[61].

## Cost of the disease on the patient (out-of-pocket expenses)

*[Criterion definition***]**: Out-of-pocket costs avoided (need for treatment or use of health resources) through implementation of the preventive measure, to RSV-infected people or their caregivers.

**[***Type of criterion***]**: Relative

**[***Evidence available***].**

No specific evidence is available for this criterion. Therefore, it must be assumed to what extent the relative improvement in health produced by nirsevimab would translate into lower out-of-pocket costs compared with placebo.

A North American study estimated average out-of-pocket expenses of $643 for hospitalized preterm infants and $214.42 for hospitalized term infants. These expenses consist of transportation, parking, food, day care, and other expenses.[62].

# Social benefits

## Impact on health inequity

*[Criterion definition***]**: Extent to which the application of the preventive measure helps to reduce health inequities between people in terms of unnecessary, avoidable, and unfair differences in the level of access and health of the population.

**[***Type of criterion***]**: Relative

**[***Evidence available***].**

The World Health Organization (WHO) defines equity in health as "the absence of unfair, avoidable or remediable differences between groups of people, whether those groups are defined socially, economically, demographically, geographically or by other dimensions of inequality (e.g., sex, gender, ethnicity, disability or sexual orientation). Health is a fundamental human right. Health equity is achieved when everyone is able to reach their full potential for health and well-being".[63].

Due to the fact that **nirsevimab** has not been introduced in the market, we do not have evidence about possible inequities that may be generated later. What we do know is that it is intended to be used for population-based prevention covering the entire population for whom it is indicated.

## Public health awareness (including antibiotic resistance)

*[Criterion definition***]**: Extent to which the use of the preventive measure helps to improve social awareness of public health, RSV and the efforts needed to raise public awareness, considering the problems it can generate, including antibiotic resistance.

**[***Type of criterion***]**: Relative

**[***Evidence available***].**

WHO recognizes RSV as a public health problem, and advocates for finding a comprehensive RSV prevention strategy that can be extended to all children, including healthy newborns[64].

According to a survey of 775 parents of children under 3 years of age, the level of social awareness of RSV among the general population is low[65]:

- Only 18% of parents claim to know the disease very well .[65]. For parents of medically attended preterm infants, this awareness is higher (61% report having good knowledge; 20% very good knowledge[66]).
- Only 22% of parents say they are very well prepared to prevent RSV. This number is higher among doctors, nurses, teachers, and daycare workers (45%).
- Only 38% of parents have consulted their doctors about preventive measures for RSV. 67% of parents say that doctors should be the first source of consultation.
- More mothers say they would get the RSV vaccine using a preventive measure approved by an institution such as the FDA (83%) than say they would get the flu vaccine when they are pregnant (60%).

Another important point is the overuse of antibiotics, which are used unnecessarily in about one-third of RSV ALRTI cases.[67]. Global antibiotic resistance is one of the problems highlighted by various international agencies, which have called it "the silent pandemic". A comprehensive survey of 12 countries conducted by WHO on this issue indicates that the majority of respondents correctly believe that many infections are becoming increasingly resistant to antibiotic treatment (72%).[68]. However, the majority also incorrectly believe that antibiotic resistance occurs when their body becomes resistant to antibiotics (76%), when in fact it is bacteria, not humans, that become resistant to antibiotics. Finally, the fact that 44% of respondents think that antibiotic resistance is only a problem for people who take antibiotics regularly is further evidence of the general misunderstanding about this issue.[68]. The fight against antibiotic resistance is singled out as one of the *One Health* objectives: every time an infection is avoided, a possible antibiotic treatment, whether indicated or not, is avoided.

Based on this information, the current situation, i.e., without any prevention or treatment measures that are effective and cover the majority of the population, the level of social awareness of RSV is very low, being higher in the population of parents/caregivers whose children have suffered from this disease.

## Innovation stimulus

*[Criterion definition***]**: Level of stimulus generated following funding of the preventive measure, for more laboratories to continue to produce innovations that improve population health and meet unmet needs for RSV prevention or treatment.

*[Type of criterion***]**: Absolute

**[***Evidence available***].**

In 2019, WHO advocated on finding a comprehensive and innovative RSV prevention strategy that can be extended to all children, including healthy newborns, and that provides for the following features.[64,69]:

1. Protection against RSV as soon as possible after birth.
2. Prophylaxis of all children <6 months.
3. A single intramuscular or subcutaneous dose can be administered at birth or at any of the regular visits in the first 6 months of life.
4. Efficacy ≥70% against severe RSV infections for 5 months after administration (average season length).
5. Safety comparable to other childhood vaccines.
6. Characteristics that allow its co-administration without interference with other vaccines in the childhood vaccination schedule.
7. Protection against both subtypes of RSV (A and B), which requires that it acts against the RSV F protein.
8. Universal access, including in low- and middle-income countries

For the assessment of this criterion, the level of stimulus generated by possible funding for nirsevimab should be indicated, so that more laboratories continue to produce innovations that improve population health and cover unmet needs for prevention and treatment.

Some questions that can be raised are: after the innovation of nirsevimab, which seems to meet most of the criteria recommended by the WHO, what will be the industry's willingness to continue producing new drugs for RSV, and what will be the Ministry of Health's willingness to approve such drugs?

# List of abbreviations

ALRTI acute lower respiratory tract infections

CHD congenital heart disease

CHMP Committee for the Evaluation of Medicinal Products for Human Use (EMA)

CLD chronic lung disease

ED emergency department

EMA European Medicines Agency

GCP Good Clinical Practices

ICH International Conference on Harmonization of Technical Requirements for Pharmaceuticals for Human Use

MCDA multi-criteria decision analysis

OR odd ratio

R_0_ R-naught, basic reproduction number

RSV respiratory syncytial virus

WHO World Health Organization

# References

[1] Hammitt LL, Dagan R, Yuan Y, et al. Nirsevimab for Prevention of RSV in Healthy Late-Preterm and Term Infants. N Engl J Med. 2022;386:837–846.

[2] Sanofi. Nirsevimab EMA regulatory submission accepted under accelerated assessment for RSV protection in all infants [Internet]. 2022. Available from: https://www.sanofi.com/media-room/press-releases/2022/2022-02-17 14-00-00 2387084.

[3] EMA. New medicine to protect babies and infants from respiratory syncytial virus (RSV) infection [Internet]. Eur. Med. Agency. 2022 [cited 2022 Sep 22]. Available from: https://www.ema.europa.eu/en/news/new-medicine-protect-babies-infants-respiratory-syncytial-virus-rsv-infection.

[4] Openshaw PJM, Chiu C, Culley FJ, et al. Protective and Harmful Immunity to RSV Infection. Annu Rev Immunol. 2017;35:501–532.

[5] Smith DK, Seales S, Budzik C. Respiratory Syncytial Virus Bronchiolitis in Children. Am Fam Physician. 2017;95:94–99.

[6] Pérez-Yarza EG, Moreno A, Lázaro P, et al. The association between respiratory syncytial virus infection and the development of childhood asthma: a systematic review of the literature. Pediatr Infect Dis J. 2007;26:733–739.

[7] Heppe-Montero M, Walter S, Hernández-Barrera V, et al. Burden of respiratory syncytial virus-associated lower respiratory infections in children in Spain from 2012 to 2018. BMC Infect Dis. 2022;22:315.

[8] Heppe Montero M, Gil-Prieto R, Walter S, et al. Burden of severe bronchiolitis in children up to 2 years of age in Spain from 2012 to 2017. Hum Vaccines Immunother. 2021;1–7.

[9] Phillips M, Finelli L, Saiman L, et al. Respiratory Syncytial Virus-associated Acute Otitis Media in Infants and Children. J Pediatr Infect Dis Soc. 2020;9:544–550.

[10] Heikkinen T, Thint M, Chonmaitree T. Prevalence of various respiratory viruses in the middle ear during acute otitis media. N Engl J Med. 1999;340:260–264.

[11] Piedimonte G, Perez MK. Respiratory Syncytial Virus Infection and Bronchiolitis. Pediatr Rev. 2014;35:519–530.

[12] Houben ML, Bont L, Wilbrink B, et al. Clinical prediction rule for RSV bronchiolitis in healthy newborns: prognostic birth cohort study. Pediatrics. 2011;127:35–41.

[13] Heikkinen T, Ojala E, Waris M. Clinical and Socioeconomic Burden of Respiratory Syncytial Virus Infection in Children. J Infect Dis. 2017;215:17–23.

[14] Carbonell-Estrany X, Pérez-Yarza EG, García LS, et al. Long-Term Burden and Respiratory Effects of Respiratory Syncytial Virus Hospitalization in Preterm Infants—The SPRING Study. Schildgen O, editor. PLOS ONE. 2015;10:e0125422.

[15] Sanchez-Luna M, Burgos-Pol R, Oyagüez I, et al. Cost-utility analysis of Palivizumab for Respiratory Syncytial Virus infection prophylaxis in preterm infants: update based on the clinical evidence in Spain. BMC Infect Dis. 2017;17:687.

[16] Hall CB. Respiratory syncytial virus and parainfluenza virus. N Engl J Med. 2001;344:1917–1928.

[17] Fauroux B, Simões EAF, Checchia PA, et al. The Burden and Long-term Respiratory Morbidity Associated with Respiratory Syncytial Virus Infection in Early Childhood. Infect Dis Ther. 2017;6:173–197.

[18] Korppi M, Piippo-Savolainen E, Korhonen K, et al. Respiratory morbidity 20 years after RSV infection in infancy. Pediatr Pulmonol. 2004;38:155–160.

[19] Glezen WP, Taber LH, Frank AL, et al. Risk of primary infection and reinfection with respiratory syncytial virus. Am J Dis Child 1960. 1986;140:543–546.

[20] Ralston SL, Lieberthal AS, Meissner HC, et al. Clinical practice guideline: the diagnosis, management, and prevention of bronchiolitis. Pediatrics. 2014;134:e1474-1502.

[21] Japanese Ministry of Health, Labour and Welfare. Preguntas y respuestas sobre la infección por virus sincitial respiratorio [Internet]. 2014 [cited 2021 Oct 19]. Available from: https://www.mhlw.go.jp/bunya/kenkou/kekkaku-kansenshou19/rs_qa.html.

[22] Garcés Sánchez M, Martinón Torres F, Platero L, et al. Carga clínica y económica del virus respiratorio sincitial en el entorno ambulatorio. Rev Pediatr Aten Primaria Supl [Internet]. 2022; Available from: https://pap.es/articulo/13581/carga-clinica-y-economica-del-virus-respiratorio-sincitial-en-el-entorno-ambulatorio.

[23] Martinón-Torres F, Carmo M, Platero L, et al. Hospital Burden of Acute Lower Respiratory Infection due to Respiratory Syncytial Virus in Spanish Children. ESPID Athens 9-13th May [Internet]. 2022; Available from: https://espid2023.kenes.com/wp-content/uploads/sites/19/2022/06/ESPID22-Abstracts-Book.pdf?_ga=2.201092462.1368452411.1666684744-2064529972.1664876059.

[24] BARI study. Results for the burden of hospitalizations for RSV in Spain 2015-2018. 2021b.

[25] MSCBS. Subdirección General de Información Sanitaria. Registro de Actividad de Atención Especializada – RAE-CMBD. [Internet]. 2021 [cited 2021 May 7]. Available from: https://pestadistico.inteligenciadegestion.mscbs.es/publicoSNS/S/rae-cmbd.

[26] Zhou H, Thompson WW, Viboud CG, et al. Hospitalizations associated with influenza and respiratory syncytial virus in the United States, 1993-2008. Clin Infect Dis Off Publ Infect Dis Soc Am. 2012;54:1427–1436.

[27] Cleveland Clinic. Respiratory Syncytial Virus (RSV): Symptoms, Treatment, Relief [Internet]. Clevel. Clin. Available from: https://my.clevelandclinic.org/health/diseases/8282-respiratory-syncytial-virus-in-children-and-adults.

[28] AEMPS. Synagis®: Ficha técnica o resumen de las características del producto [Internet]. 2009 [cited 2021 May 19]. Available from: https://cima.aemps.es/cima/dochtml/ft/199117003/FT_199117003.html.

[29] Sánchez Luna M, Pérez Muñuzuri A, Leante Castellanos JL, et al. Recomendaciones de la Sociedad Española de Neonatología para la utilización de palivizumab como profilaxis de las infecciones graves por el virus respiratorio sincitial en lactantes de alto riesgo, actualización. An Pediatría. 2019;91:348–350.

[30] Stewart DL, Ryan KJ, Seare JG, et al. Association of RSV-related hospitalization and non-compliance with palivizumab among commercially insured infants: a retrospective claims analysis. BMC Infect Dis. 2013;13:334.

[31] Krilov LR, Masaquel AS, Weiner LB, et al. Partial palivizumab prophylaxis and increased risk of hospitalization due to respiratory syncytial virus in a Medicaid population: a retrospective cohort analysis. BMC Pediatr. 2014;14:261.

[32] Consejo General de Colegios Oficiales de Farmacéuticos. Bot Plus 2.0 Base de Datos de Medicamentos [Internet]. [cited 2021 Jan 21]. Available from: https://botplusweb.portalfarma.com/botplus.aspx.

[33] AEP y NEUMOPED. Protocolos diagnósticos y terapéuticos en Neumología pediátrica [Internet]. 2017. Available from: https://neumoped.org/wp-content/uploads/2019/02/Protocolos-Diagn%C3%B3sticos-y-terap%C3%A9uticos-en-Neumolog%C3%ADa-Pedi%C3%A1trica.pdf.

[34] AEP. Virus Respiratorio Sincicial (VRS) [Internet]. [cited 2021 Apr 19]. Available from: https://www.aeped.es/sites/default/files/documentos/vrs.pdf.

[35] Sruamsiri R, Kubo H, Mahlich J. Hospitalization costs and length of stay of Japanese children with respiratory syncytial virus: A structural equation modeling approach. Medicine (Baltimore). 2018;97:e11491.

[36] ClinicalTrials.gov. A Phase 3 Randomized, Double-blind, Placebo-controlled Study to Evaluate the Safety and Efficacy of MEDI8897, a Monoclonal Antibody With an Extended Half-life Against Respiratory Syncytial Virus, in Healthy Late Preterm and Term Infants (MELODY), NCT03979313 [Internet]. 2022. Available from: https://clinicaltrials.gov/ct2/show/NCT03979313.

[37] Simões E, Madhi S, Zar H, et al. Pooled efficacy of Nirsevimab against RSV Lower Respiratory Tract Infection in Preterm and Term Infants [oral presentation O001 / #889]. ESPID Athens 9-13th May [Internet]. 2022; Available from: https://keneswp.azureedge.net/wp-content/uploads/sites/162/2022/05/ESPID22-Abstracts-Book.pdf.

[38] Griffin MP, Yuan Y, Takas T, et al. Single-Dose Nirsevimab for Prevention of RSV in Preterm Infants. N Engl J Med. 2020;383:415–425.

[39] Reis J, Shaman J. Simulation of four respiratory viruses and inference of epidemiological parameters. Infect Dis Model. 2018;3:23–34.

[40] Yamin D, Jones FK, DeVincenzo JP, et al. Vaccination strategies against respiratory syncytial virus. Proc Natl Acad Sci U S A. 2016;113:13239–13244.

[41] ClinicalTrials.gov. A Phase 2b Randomized, Double-Blind, Placebo-controlled Study to Evaluate the Safety and Efficacy of MEDI8897, a Monoclonal Antibody With an Extended Half-life Against Respiratory Syncytial Virus, in Healthy Preterm Infants [Internet]. 2019. Available from: https://clinicaltrials.gov/ct2/show/NCT02878330.

[42] Orrico-Sánchez A, Gómez-Álvarez C, López-Lacort M, et al. Pérdida de calidad de vida por virus respiratorio sincitial (VRS) en lactantes en España. Vacunas. 2019;20:24.

[43] Crépey P, Redondo E, Díez-Domingo J, et al. From trivalent to quadrivalent influenza vaccines: Public health and economic burden for different immunization strategies in Spain. PLOS ONE. 2020;15:e0233526.

[44] Ministerio de Sanidad, Servicios Sociales e Igualdad. Servicio, de Evaluación del Servicio Canario de la Salud. Revisión sistemática de utilidades o preferencias por los estados de salud obtenidas de población española. Informes de Evaluación de Tecnologías Sanitarias SESCS [Internet]. 2011. Available from: http://funcanis.es/wp-content/uploads/2015/09/SESCS-2011_R.S.-Preferencias-E.S._sin.pdf.

[45] Pokrzywinski RM, Swett LL, Pannaraj PS, et al. Impact of Respiratory Syncytial Virus–Confirmed Hospitalizations on Caregivers of US Preterm Infants. Clin Pediatr (Phila). 2019;58:837–850.

[46] Falsey AR, Hennessey PA, Formica MA, et al. Respiratory syncytial virus infection in elderly and high-risk adults. N Engl J Med. 2005;352:1749–1759.

[47] Walsh EE, Peterson DR, Kalkanoglu AE, et al. Viral Shedding and Immune Responses to Respiratory Syncytial Virus Infection in Older Adults. J Infect Dis. 2013;207:1424–1432.

[48] Kwong JC, Schwartz KL, Campitelli MA, et al. Acute Myocardial Infarction after Laboratory-Confirmed Influenza Infection. N Engl J Med. 2018;378:345–353.

[49] Mao Z, Li X, Korsten K, et al. Economic Burden and Health-Related Quality of Life of Respiratory Syncytial Virus and Influenza Infection in European Community-Dwelling Older Adults. J Infect Dis. 2022;jiac069.

[50] Nam HH, Ison MG. Respiratory syncytial virus infection in adults. BMJ. 2019;366:l5021.

[51] Leader S, Yang H, DeVincenzo J, et al. Time and Out-of-Pocket Costs Associated with Respiratory Syncytial Virus Hospitalization of Infants. Value Health. 2003;6:100–106.

[52] Leidy NK. The Impact of Severe Respiratory Syncytial Virus on the Child, Caregiver, and Family During Hospitalization and Recovery. PEDIATRICS. 2005;115:1536–1546.

[53] Young M, Smitherman L. Socioeconomic Impact of RSV Hospitalization. Infect Dis Ther. 2021;10:35–45.

[54] Nuijten MJ, Wittenberg W. Cost effectiveness of palivizumab in Spain: an analysis using observational data. Eur J Health Econ HEPAC Health Econ Prev Care. 2010;11:105–115.

[55] INE. Actualización de rentas con el IPC general (sistema IPC base 2021) para periodos anuales completos [Internet]. 2022. Available from: https://www.ine.es/calcula/.

[56] INE. Movimiento natural de la población: Nacimientos [Internet]. 2021. Available from: https://www.ine.es/dyngs/INEbase/es/operacion.htm?c=Estadistica_C&cid=1254736177007&menu=ultiDatos&idp=1254735573002.

[57] Drago G, Roiz J, Kazmierska P, et al. Modeled impact of Nirsevimab against respiratory syncytial virus (RSV) AMONG Spanish infants experiencing their first RSV season [oral presentation O024 / #1329]. ESPID Athens 9-13th May [Internet]. 2022; Available from: https://keneswp.azureedge.net/wp-content/uploads/sites/162/2022/05/ESPID22-Abstracts-Book.pdf.

[58] Beuvelet M, Chung-Delgado K, Kieffer A. PIN29 Cost-Effectiveness of Nirsevimab Against Respiratory Syncytial VIRUS (RSV) Among US Infants Experiencing Their First RSV Season. Value Health. 2021;24:S110.

[59] BARI study. Results for the burden of RSV in Spain: Longitudinal Patient Data 2017-2018. 2021a.

[60] Instituto Nacional de Estadística. Datos del padrón continuo. 2021.

[61] Garcia-Marcos L, Valverde-Molina J, Pavlovic-Nesic S, et al. Pediatricians’ attitudes and costs of bronchiolitis in the emergency department: a prospective multicentre study. Pediatr Pulmonol. 2014;49:1011–1019.

[62] Leader S, Yang H, DeVincenzo J, et al. Time and Out-of-Pocket Costs Associated with Respiratory Syncytial Virus Hospitalization of Infants. Value Health. 2003;6:100–106.

[63] OMS. Health Equity -- Global [Internet]. [cited 2022 May 20]. Available from: https://www.who.int/health-topics/health-equity.

[64] WHO. WHO preferred product characteristics of monoclonal antibodies for passive immunization against respiratory syncytial virus (‎RSV)‎ disease [Internet]. 2021 [cited 2021 Aug 24]. Available from: https://www.who.int/publications-detail-redirect/9789240021853.

[65] Alliance for Patient Access (AfPA). National RSV awareness survey [Internet]. 2018. Available from: https://static1.squarespace.com/static/5523fcf7e4b0fef011e668e6/t/5bdb422521c67c69e6c09e4a/1541095976941/RSV+Online+Survey+Key+Findings+Deck.pdf.

[66] Bracht M, Bacchini F, Paes B. A Survey of Parental Knowledge of Respiratory Syncytial Virus and Other Respiratory Infections in Preterm Infants. Neonatal Netw NN. 2021;40:14–24.

[67] van Houten CB, Naaktgeboren C, Buiteman BJM, et al. Antibiotic Overuse in Children with Respiratory Syncytial Virus Lower Respiratory Tract Infection. Pediatr Infect Dis J. 2018;37:1077–1081.

[68] OMS. Antibiotic resistance: multi-country public awareness survey [Internet]. 2015. Available from: https://apps.who.int/iris/bitstream/handle/10665/194460/9789241509817_eng.pdf;jsessionid=0C8E46DDB07F4AAAA7B97AEE5BD5F33D?sequence=1.

[69] Vekemans J, Moorthy V, Giersing B, et al. Respiratory syncytial virus vaccine research and development: World Health Organization technological roadmap and preferred product characteristics. Vaccine. 2019;37:7394–7395.

1. in which the patient himself recovers after a few days. [↑](#footnote-ref-2)
2. alveolar inflammation; 0.05% of children ≤12 months in Spain.[7] [↑](#footnote-ref-3)
3. Occlusion of the smallest airways; 2.2% of children ≤12 months in Spain.[7] [↑](#footnote-ref-4)
4. fast breathing [↑](#footnote-ref-5)
5. bluish coloration of the skin due to insufficient oxygenation of the blood. [↑](#footnote-ref-6)
6. abnormal decrease in the partial pressure of oxygen in the arterial blood below 60 mmHg [↑](#footnote-ref-7)
7. average number of visits per infected patient: 9.0, according to the RSV + acute bronchiolitis population definition. [↑](#footnote-ref-8)
8. average number of visits per infected patient: 2.0[22] [↑](#footnote-ref-9)
9. according to the study of hospitalizations, 3 seasons: 2015-2018.[23,p.2015–2018] [↑](#footnote-ref-10)
10. infants born prematurely or with heart or lung disease[28,29] [↑](#footnote-ref-11)
11. 15 mg/kg every 30 days up to a maximum of 5 doses, administered in hospital [↑](#footnote-ref-12)
12. children and infants with respiratory pathology or underlying heart disease and premature infants [↑](#footnote-ref-13)
13. Definition of primary efficacy endpoint on medically attended RSV-associated lower respiratory tract infection: RSV case is a patient who must meet at least two of the criteria specified in the study protocol: RSV-positive CRP, physical finding of lower respiratory tract involvement, and an objective measure of clinical severity. [↑](#footnote-ref-14)
14. reported by the caregiver, on a scale of 1 to 7, where 1 means no stress and 7 means a lot of stress. [↑](#footnote-ref-15)
15. such as maternal vaccination, for example, or vaccination of populations other than children ≤12 months of age. [↑](#footnote-ref-16)
16. measured by the "Work Productivity and Activity Impairment: Hospitalization of the Child for Respiratory Illness" (WPAI:CHRI) questionnaire. [↑](#footnote-ref-17)
17. RSV population *vs.* RSV + bronchiolitis population [↑](#footnote-ref-18)
18. 29-35 weeks gestational age. [↑](#footnote-ref-19)
