# Supplementary material for: Evaluation of antibody-based preventive alternatives for respiratory syncytial virus: a novel multi-criteria decision analysis framework and assessment of nirsevimab in Spain
Source: BMC Infect Dis. 2024 Jan 18;24:99. doi: 10.1186/s12879-024-08988-9 (PMC10797756; doi:10.1186/s12879-024-08988-9)
Supplement: Supplementary file 3 — Supplementary Material 3: Criteria selection [file 12879_2024_8988_MOESM3_ESM.docx]

**Evaluation of Antibody-based Preventive Alternatives for Respiratory Syncytial Virus: A Novel Multi-Criteria Decision Analysis Framework and Assessment of Nirsevimab in Spain**

**Authors**: Jorge Mestre-Ferrándiz^1^, Agustín Rivero^2^, Alejandro Orrico-Sánchez^3,4,5^, Álvaro Hidalgo^6,7^, Fernando Abdalla^8^, Isabel Martín^9^, Javier Álvarez^10^, Manuel García-Cenoz^11^, Maria del Carmen Pacheco^12^, María Garcés-Sánchez^13^, Néboa Zozaya^8,14^, Raúl Ortiz-de-Lejarazu^15^

**Affiliations**: ^1^Department of Economics, University Carlos III, Madrid, Spain; ^2^Department of Management, Bioregión de Salud y Bienestar (BioMad), Madrid, Spain; ^3^Department of Vaccines Research, Fundación Para el Fomento de la Investigación Sanitaria y Biomédica de la Comunitat Valenciana (Fisabio), Valencia, Spain; ^4^Catholic University of Valencia, Spain; ^5^Centro de Investigación Biomédica en Red de Epidemiología y Salud Pública (CIBERESP); ^6^Weber Foundation, Madrid, Spain; ^7^Department of Economic Analysis and Finances, University of Castilla-La Mancha. Toledo, Spain; ^8^Department of Health Affairs and Policy Research, Vivactis Weber, Madrid, Spain; ^9^Department of Primary Care, Rochapea Healthcare Center, Navarra, Spain; ^10^Department of Pediatrics, Hospital Costa del Sol, Málaga, Spain; ^11^Public Health Institute of Navarra, Navarra, Spain; ^12^Department of Epidemiology, General Directorate of Public Health, Castilla y León, Spain; ^13^Department of Pediatrics, Nazaret Healthcare Center, Valencia, Spain; ^14^Department of Quantitative Methods in Economics and Management, University Las Palmas de Gran Canaria. Las Palmas, Spain; ^15^National Influenza Centre, Scientific Advisor and Emeritus Director, School of Medicine, University of Valladolid, Castilla y León, Spain.

**SUPPLEMENTARY FILE 3: CRITERIA SELECTION**

# **Context**

This MCDA in RSV was conducted through an *ad hoc* framework (set of criteria), starting from a preset value approach aimed at vaccine evaluation (Vaccinex) [1]. Vaccinex is a model developed through a survey conducted in a population sample (n = 1000) in Flanders, Belgium, in which support for the use of these broader criteria to evaluate vaccines in funding decisions was investigated through a hypothetical vaccine called Vaccinex. The original framework consists of a set of 40 possible criteria, divided into the two domains: clinical aspects of the vaccine and of the disease.

At the first meeting with the multidisciplinary committee of experts (MCE), the choice of criteria for the MCDA framework in RSV was made through a vote using an audience engagement platform (Mentimeter®, Mentimeter AB, Stockholm, Sweden), where the MCE decided whether to include (=1) or not to include (=0) each of the criteria in the proposed framework. The decision to include or not to include a criterion was based on a simple majority (>0.5) (**Table [S4].1**)

**Table (S4).1. Average of the votes of the criteria of the framework.**

| Criteria | Average | Included / not included |
| --- | --- | --- |
| Severity of symptoms | 1.000 | Included |
| Comorbidity risk | 1.000 | Included |
| Prevalence of disease | 1.000 | Included |
| Efficacy of the vaccine | 1.000 | Included |
| Vaccine cost | 1.000 | Included |
| Impact on health inequity | 1.000 | Included |
| Impact on the population of children | 1.000 | Included |
| Mortality risk | 0.875 | Included |
| Availability of treatment | 0.875 | Included |
| Certainty around the magnitude of the efficacy of the vaccine | 0.875 | Included |
| Serious adverse events | 0.875 | Included |
| Cost of disease (health care system) | 0.875 | Included |
| Impact on socioeconomically disadvantaged population | 0.875 | Included |
| Impact on the population of elderly | 0.875 | Included |
| Transmissibility | 0.750 | Included |
| Prevention alternatives | 0.750 | Included |
| Herd immunity | 0.750 | Included |
| Cost of disease (patient) | 0.750 | Included |
| Duration of symptoms | 0.625 | Included |
| Mild adverse events | 0.625 | Included |
| Productivity costs: absenteeism | 0.625 | Included |
| Public health awareness | 0.625 | Included |
| Impact on caregivers | 0.500 | Included |
| Innovation stimulus | 0.500 | included |
| Perception and fear | 0.500 | NOT included |
| Legal liability | 0.500 | NOT included |
| Eradication potential | 0.375 | NOT included |
| Mode of transmission | 0.375 | NOT included |
| Image and goodwill (intangible effects) | 0.375 | NOT included |
| Resistance offered by anti-vaccination groups | 0.375 | NOT included |
| Impact on the population of pregnant women | 0.375 | NOT included |
| Impact on school activities | 0.250 | NOT included |
| Time to development of symptoms | 0.125 | NOT included |
| Costs related to the production platform | 0.125 | NOT included |
| Productivity costs: presenteeism | 0.125 | NOT included |
| Generation of jobs in the country | 0.125 | NOT included |
| Impact on migrant population | 0.125 | NOT included |
| Impact on fertility | 0.000 | NOT included |
| Impact on the LGTBI population | 0.000 | NOT included |
| Impact on the female population | 0.000 | NOT included |

Note: 0 means not included, and 1 means included. Ranked from highest consensus to lowest consensus for inclusion. Lines highlighted in grey were the ones that had to be discussed by the committee, as an initial consensus was not achieved through voting.

# **Decisions**

Based on the votes, the MCE initially decided to include 22 criteria and exclude 14 criteria from the Vaccinex framework. The committee discussion focused on the 4 criteria where there was no consensus. The discussion held, arguments used, and the final decision regarding each of these criteria were as follows:

- **Innovation stimulus**. The experts who advocate its inclusion mention that this criterion is fundamental to stimulate pharmaceutical companies to continue producing innovations that improve the health of the population and that cover unmet needs for prevention or treatment. On the other hand, the experts who believe that this criterion should not be included commented that there are other diseases that need this stimulus, since in RSV there is already a "snowball" that has been set in motion and will continue, despite the absence of this stimulus. It was decided to include this criterion in the MCDA framework.
- **Impact on caregivers**. Some experts commented that this criterion could overlap with the indirect costs criterion (labor productivity of caregivers). To avoid this overlap, experts commented that this criterion should be limited to the impact on the quality of life and daily activities of parents of RSV-affected children. It was decided to include this criterion in the MCDA framework, focusing on the impact on quality of life and daily activities.
- **Perception and fear**. Some experts who voted for the inclusion of this criterion had interpreted it as the perception and fear of the effects of a possible incorporation of a preventive measure. They changed their position when they received the clarification that this criterion refers to the perception and fear that the population has regarding RSV. It was decided not to include this criterion in the MCDA framework.
- **Legal liability**. The experts who were in favor of the inclusion of this criterion had understood it to refer to the risk of litigation that the manufacturer of the preventive measure might suffer after the launch of the product on the market. They changed their opinion after it was explained that this criterion referred to the State level, i.e., to the risk of litigation that the public administration may suffer after the approval of the treatment. It was decided not to include this criterion in the MCDA framework.

# **Additional proposals**

Subsequent to the meeting, Vivactis Weber made a proposal for the inclusion of two additional criteria: (i) burden of disease - incidence on the outpatient setting; (ii) burden of disease - incidence on the inpatient setting. In addition, it suggested replacing the disease prevalence criterion with "population in which the prevention strategy would be indicated ". This proposal was based on the fact that these criteria, which represent important unmet needs, are not included in the Vaccinex framework. The experts agreed with Vivactis Weber's proposal.

# **Final framework**

The MCDA framework in RSV therefore had a total of 26 criteria. Vivactis Weber proposed the definitions, possible groupings into domains and the scales for scoring the test. In the second meeting, some of these aspects were corrected in order to provide greater clarity and align interpretations among the experts. The details on the final MCDA framework in RSV, which includes the names of each criterion, the domains to which they belong, the type of criterion (absolute / relative), their definitions and their scoring scales can be found in **supplementary file 5**.

# **References**

[1] Luyten J. Kessels R. Vandermeulen C. et al. Value Frameworks for Vaccines: Which Dimensions Are Most Relevant? Vaccines. 2020;8:E628.
