# Supplementary material for: Evaluation of antibody-based preventive alternatives for respiratory syncytial virus: a novel multi-criteria decision analysis framework and assessment of nirsevimab in Spain
Source: BMC Infect Dis. 2024 Jan 18;24:99. doi: 10.1186/s12879-024-08988-9 (PMC10797756; doi:10.1186/s12879-024-08988-9)
Supplement: Supplementary file 4 — Supplementary Material 4: Framework [file 12879_2024_8988_MOESM4_ESM.docx]

**Evaluation of Antibody-based Preventive Alternatives for Respiratory Syncytial Virus: A Novel Multi-Criteria Decision Analysis Framework and Assessment of Nirsevimab in Spain**

**Authors**: Jorge Mestre-Ferrándiz^1^, Agustín Rivero^2^, Alejandro Orrico-Sánchez^3,4,5^, Álvaro Hidalgo^6,7^, Fernando Abdalla^8^, Isabel Martín^9^, Javier Álvarez^10^, Manuel García-Cenoz^11^, Maria del Carmen Pacheco^12^, María Garcés-Sánchez^13^, Néboa Zozaya^8,14^, Raúl Ortiz-de-Lejarazu^15^

**Affiliations**: ^1^Department of Economics, University Carlos III, Madrid, Spain; ^2^Department of Management, Bioregión de Salud y Bienestar (BioMad), Madrid, Spain; ^3^Department of Vaccines Research, Fundación Para el Fomento de la Investigación Sanitaria y Biomédica de la Comunitat Valenciana (Fisabio), Valencia, Spain; ^4^Catholic University of Valencia, Spain; ^5^Centro de Investigación Biomédica en Red de Epidemiología y Salud Pública (CIBERESP); ^6^Weber Foundation, Madrid, Spain; ^7^Department of Economic Analysis and Finances, University of Castilla-La Mancha. Toledo, Spain; ^8^Department of Health Affairs and Policy Research, Vivactis Weber, Madrid, Spain; ^9^Department of Primary Care, Rochapea Healthcare Center, Navarra, Spain; ^10^Department of Pediatrics, Hospital Costa del Sol, Málaga, Spain; ^11^Public Health Institute of Navarra, Navarra, Spain; ^12^Department of Epidemiology, General Directorate of Public Health, Castilla y León, Spain; ^13^Department of Pediatrics, Nazaret Healthcare Center, Valencia, Spain; ^14^Department of Quantitative Methods in Economics and Management, University Las Palmas de Gran Canaria. Las Palmas, Spain; ^15^National Influenza Centre, Scientific Advisor and Emeritus Director, School of Medicine, University of Valladolid, Castilla y León, Spain.

**SUPPLEMENTARY FILE 4: FRAMEWORK**

**Table (S5).1. Details on the MCDA framework in VRS**

| **#** | **Criteria** |  | **Definition** | **Scoring scale** |
| --- | --- | --- | --- | --- |
| **Domain 1: Severity of disease** | | | | |
| **1** | **Severity of symptoms** | A | Severity of symptoms in RSV-infected people. | 5: very severe symptoms / 0: not at all severe symptoms |
| **2** | **Lethality risk** | A | Rate of deaths per number of RSV infection cases | 5: Very lethal / 0: not lethal at all |
| **3** | **Comorbidity risk** | A | Likelihood that RSV-infected people will develop other associated diseases in the short, medium or long term. | 5: very high comorbidity risk / 0: no comorbidity risk |
| **Domain 2: Burden of disease** | | | | |
| **4** | **Incidence of RSV cases** | A | Proportion of the population infected by RSV | 5: very high incidence / 0: no incidence |
| **5** | **Incidence on the outpatient setting** | A | Incidence rates for RSV infection on the outpatient setting (primary and specialty care visits). |  |
| **6** | **Incidence on the inpatient setting** | A | Incidence rates for RSV infection on the inpatient setting (emergency room visits and hospitalizations). |  |
| **7** | **Time of duration of acute symptoms** | A | Time of duration of acute symptoms in RSV-infected people. | 5: acute symptoms last for a long time / 0: acute symptoms last for a very short time |
| **Domain 3: Prevention or Treatment Alternatives** | | | | |
| **8** | **Prevention alternatives** | A | Existence, availability, effectiveness, and safety of RSV prevention alternatives. | 5: No effective prevention alternative / 0: There are many effective prevention alternatives. |
| **9** | **Availability of treatment** | A | Existence, availability, effectiveness, and safety of treatment alternatives for RSV-infected people. | 5: There are no effective treatment alternatives / 0: There are many effective treatment alternatives |
| **Domain 4: Size of population** | | | | |
| **10** | **Population in which the prevention strategy would be indicated** | R | Proportion of the population in which the prevention strategy would be indicated. | 5: Nirsevimab would be indicated to a much broader population than the comparator / 0: Nirsevimab and the comparator would be indicated to the same amount of population / -5: The comparator would be indicated to a much broader population than nirsevimab |
| **Domain 5: Efficacy** | | | | |
| **11** | **Efficacy of the preventive measure** | R | Level of efficacy of RSV preventive measure | 5: Nirsevimab is much more effective than the comparator / 0: no difference / -5: Comparator is much more effective than nirsevimab |
| **Domain 6: Population protection** | | | | |
| **12** | **Group immunity (collective protection)** | A | The application of the preventive measure in the majority of the population would also indirectly protect those who do not receive the preventive measure (since the risk of infection decreases). | 5: By applying the preventive measure to a relatively small number of people, collective protection is achieved / 0: Collective protection is only achieved if the preventive measure is applied to 100% of the population. |
| **13** | **Transmissibility** | A | Number of cases of infection occurring from a primary RSV-infected case, expressed through the basic reproductive number. | 5: Highly contagious / 0: not contagious at all |
| **Domain 7: Safety** | | | | |
| **14** | **Serious adverse events** | R | Serious adverse events occurring in people receiving the preventive measure | 5: Nirsevimab produces far fewer serious adverse events than the comparator / 0: no difference / -5: Nirsevimab produces far more serious adverse events than the comparator |
| **15** | **Mild adverse events** | R | Mild adverse events occurring in people receiving the preventive measure | 5: Nirsevimab produces far fewer mild adverse events than the comparator / 0: no difference / -5: Nirsevimab produces far more mild adverse events than the comparator |
| **Domain 8: Quality of evidence** | | | | |
| **16** | **Certainty about the efficacy of the preventive measure** | A | Level of certainty about the efficacy of the preventive measure | 5: Highly relevant and valid evidence, with high certainty / 0: Non-relevant and invalid evidence, with low certainty |
| **Domain 9: Impact on quality of life** | | | | |
| **17** | **Impact on the population of children** | A | Level of impairment (stress, quality of life) produced in RSV-infected children. | 5: Very high impact (stress and quality of life) / 0: no impact (stress and quality of life) |
| **18** | **Impact on the population over 65 years of age** | A | Level of impairment (stress, quality of life) in RSV-infected people over 65 years of age. |  |
| **19** | **Impact on caregivers** | A | Number of hours dedicated by caregivers to RSV-infected people, and level of impairment (stress, quality of life) produced on these caregivers. | 5: Very high impact (stress and quality of life) and many hours of dedication / 0: no impact (stress and quality of life) and no necessary dedication |
| **Domain 10: Acquisition cost** | | | | |
| **20** | **Monetary cost of the preventive measure** | R | Cost of acquisition of the preventive measure | 5: Nirsevimab generates substantial savings in acquisition costs vs. comparator / 0: no difference / -5: Nirsevimab generates substantial additional expenditures in acquisition costs vs. comparator |
| **Domain 11: Impact on other costs** | | | | |
| **21** | **Cost of the disease on the health system (excludes acquisition cost)** | R | Costs avoided by the use of the preventive measure, in relation to the use of health resources (primary care, specialized care, emergency and hospitalization) of people infected by RSV, excluding the cost of acquisition of the preventive measure. | 5: Nirsevimab generates substantial savings vs. comparator / 0: no difference / -5: Nirsevimab generates substantial additional costs vs. comparator |
| **22** | **Productivity cost: absenteeism** | R | Indirect costs avoided for society due to absenteeism of caregivers, as a consequence of the use of the preventive measure. |  |
| **23** | **Cost of the disease on the patient (out-of-pocket expenses)** | R | Avoided out-of-pocket expenses (need for treatment or use of healthcare resources) through the implementation of the preventive measure, to RSV-infected individuals or their caregivers. |  |
| **Domain 12: Social benefits** | | | | |
| **24** | **Impact on health inequity** | R | Extent to which the application of the preventive measure helps to reduce health inequities among individuals in terms of unnecessary, avoidable, and unfair differences on the level of access and health of the population. | 5: Nirsevimab substantially reduces health inequity vs. comparator / 0: no difference / -5: Nirsevimab substantially increases health inequity vs. comparator |
| **25** | **Public health awareness (including antibiotic resistance)** | R | Extent to which the use of the preventive measure helps to improve social awareness of public health, RSV and the efforts needed to raise public awareness, considering the problems it can generate, including antibiotic resistance. | 5: Nirsevimab substantially increases public health awareness vs. comparator / 0: no difference / -5: Nirsevimab substantially decreases public health awareness vs. comparator |
| **26** | **Innovation stimulus** | A | Level of stimulus generated after funding is granted to the preventive measure, so that more pharmaceutical companies continue to produce innovations that improve the health of the population and meet the unmet needs for RSV prevention or treatment. | 5: Funding for nirsevimab will result in many more pharmaceutical companies continuing to produce innovations that improve population health / 0: Funding for nirse will result in no pharmaceutical companies continuing to produce innovations that improve population health. |

A: absolute. R: relative. RSV: respiratory syncytial virus
