# Supplementary material for: Evaluation of antibody-based preventive alternatives for respiratory syncytial virus: a novel multi-criteria decision analysis framework and assessment of nirsevimab in Spain
Source: BMC Infect Dis. 2024 Jan 18;24:99. doi: 10.1186/s12879-024-08988-9 (PMC10797756; doi:10.1186/s12879-024-08988-9)
Supplement: Supplementary file 5 — Supplementary Material 5: Weights, scores and value estimates [file 12879_2024_8988_MOESM5_ESM.docx]

**Evaluation of Antibody-based Preventive Alternatives for Respiratory Syncytial Virus: A Novel Multi-Criteria Decision Analysis Framework and Assessment of Nirsevimab in Spain**

**Authors**: Jorge Mestre-Ferrándiz^1^, Agustín Rivero^2^, Alejandro Orrico-Sánchez^3,4,5^, Álvaro Hidalgo^6,7^, Fernando Abdalla^8^, Isabel Martín^9^, Javier Álvarez^10^, Manuel García-Cenoz^11^, Maria del Carmen Pacheco^12^, María Garcés-Sánchez^13^, Néboa Zozaya^8,14^, Raúl Ortiz-de-Lejarazu^15^

**Affiliations**: ^1^Department of Economics, University Carlos III, Madrid, Spain; ^2^Department of Management, Bioregión de Salud y Bienestar (BioMad), Madrid, Spain; ^3^Department of Vaccines Research, Fundación Para el Fomento de la Investigación Sanitaria y Biomédica de la Comunitat Valenciana (Fisabio), Valencia, Spain; ^4^Catholic University of Valencia, Spain; ^5^Centro de Investigación Biomédica en Red de Epidemiología y Salud Pública (CIBERESP); ^6^Weber Foundation, Madrid, Spain; ^7^Department of Economic Analysis and Finances, University of Castilla-La Mancha. Toledo, Spain; ^8^Department of Health Affairs and Policy Research, Vivactis Weber, Madrid, Spain; ^9^Department of Primary Care, Rochapea Healthcare Center, Navarra, Spain; ^10^Department of Pediatrics, Hospital Costa del Sol, Málaga, Spain; ^11^Public Health Institute of Navarra, Navarra, Spain; ^12^Department of Epidemiology, General Directorate of Public Health, Castilla y León, Spain; ^13^Department of Pediatrics, Nazaret Healthcare Center, Valencia, Spain; ^14^Department of Quantitative Methods in Economics and Management, University Las Palmas de Gran Canaria. Las Palmas, Spain; ^15^National Influenza Centre, Scientific Advisor and Emeritus Director, School of Medicine, University of Valladolid, Castilla y León, Spain.

**SUPPLEMENTARY FILE 5: WEIGHTS, SCORES AND VALUE ESTIMATES**

***Overall and by subgroup***

**Index**

[**Table (S6).1: Weights, scale 1-5, overall and by subgroup** 3](#_Toc135393653)

[**Table (S6).2: Scores, overall and by subgroup, nirsevimab vs. placebo** 4](#_Toc135393654)

[**Table (S6).3: Value estimates, overall and by subgroup, nirsevimab vs. placebo** 5](#_Toc135393655)

**Table (S6).1: Weights, scale 1-5, overall and by subgroup**

Mean ± standard deviation (median) [min to max]

| Criteria | Overall (n=9) | Clinicians (n=4) | Managers (n=5) |
| --- | --- | --- | --- |
| **Domain 1: Severity of disease** |  |  |  |
| Severity of symptoms | 4.4 ± 0.7 (5.0) [3 to 5] | 4.5 ± 0.6 (5.0) [4 to 5] | 4.4 ± 0.9 (5.0) [3 to 5] |
| Lethality risk | 4.2 ± 1.2 (5.0) [2 to 5] | 4.3 ± 1.5 (5.0) [2 to 5] | 4.2 ± 1.1 (5.0) [3 to 5] |
| Comorbidity risk | 3.9 ± 0.3 (4.0) [3 to 4] | 3.8 ± 0.5 (4.0) [3 to 4] | 4.0 ± 0.0 (4.0) [4 to 4] |
| **Domain 2: Burden of disease** |  |  |  |
| Incidence of RSV cases | 4.0 ± 0.9 (4.0) [3 to 5] | 3.8 ± 1.0 (4.0) [3 to 5] | 4.2 ± 0.8 (4.0) [3 to 5] |
| Incidence on the outpatient setting | 4.1 ± 0.9 (4.0) [2 to 5] | 3.8 ± 1.3 (4.0) [2 to 5] | 4.4 ± 0.5 (4.0) [4 to 5] |
| Incidence on the inpatient setting | 4.4 ± 0.9 (5.0) [3 to 5] | 4.5 ± 1.0 (5.0) [3 to 5] | 4.4 ± 0.9 (5.0) [3 to 5] |
| Time of duration of acute symptoms | 3.6 ± 0.7 (3.0) [3 to 5] | 3.5 ± 0.6 (4.0) [3 to 4] | 3.6 ± 0.9 (3.0) [3 to 5] |
| **Domain 3: Prevention or Treatment Alternatives** |  |  |  |
| Prevention alternatives | 3.7 ± 1.0 (4.0) [2 to 5] | 3.8 ± 1.0 (4.0) [3 to 5] | 3.6 ± 1.1 (4.0) [2 to 5] |
| Availability of treatment | 4.0 ± 1.5 (5.0) [1 to 5] | 4.0 ± 2.0 (5.0) [1 to 5] | 4.0 ± 1.2 (4.0) [2 to 5] |
| **Domain 4: Size of population** |  |  |  |
| Population in which the prevention strategy would be indicated | 3.7 ± 1.2 (4.0) [2 to 5] | 2.8 ± 1.0 (3.0) [2 to 4] | 4.4 ± 0.9 (5.0) [3 to 5] |
| **Domain 5: Efficacy** |  |  |  |
| Efficacy of the preventive measure | 4.6 ± 0.7 (5.0) [3 to 5] | 4.8 ± 0.5 (5.0) [4 to 5] | 4.4 ± 0.9 (5.0) [3 to 5] |
| **Domain 6: Population protection** |  |  |  |
| Group immunity (collective protection) | 3.3 ± 1.1 (3.0) [1 to 5] | 2.5 ± 1.0 (3.0) [1 to 3] | 4.0 ± 0.7 (4.0) [3 to 5] |
| Transmissibility | 4.0 ± 0.9 (4.0) [2 to 5] | 3.5 ± 1.0 (4.0) [2 to 4] | 4.4 ± 0.5 (4.0) [4 to 5] |
| **Domain 7: safety** |  |  |  |
| Serious adverse events | 4.2 ± 1.3 (5.0) [2 to 5] | 3.5 ± 1.7 (4.0) [2 to 5] | 4.8 ± 0.4 (5.0) [4 to 5] |
| Mild adverse events | 3.1 ± 1.1 (3.0) [2 to 5] | 2.8 ± 1.5 (2.0) [2 to 5] | 3.4 ± 0.5 (3.0) [3 to 4] |
| **Domain 8: Quality of evidence** |  |  |  |
| Certainty about the efficacy of the preventive measure | 4.0 ± 0.9 (4.0) [3 to 5] | 4.3 ± 1.0 (5.0) [3 to 5] | 3.8 ± 0.8 (4.0) [3 to 5] |
| **Domain 9: Impact on quality of life** |  |  |  |
| Impact on the population of children | 4.1 ± 0.9 (4.0) [2 to 5] | 3.8 ± 1.3 (4.0) [2 to 5] | 4.4 ± 0.5 (4.0) [4 to 5] |
| Impact on the population over 65 years of age | 3.6 ± 0.9 (4.0) [2 to 5] | 3.3 ± 1.0 (4.0) [2 to 4] | 3.8 ± 0.8 (4.0) [3 to 5] |
| Impact on caregivers | 2.6 ± 1.0 (2.0) [1 to 4] | 2.5 ± 1.3 (3.0) [1 to 4] | 2.6 ± 0.9 (2.0) [2 to 4] |
| **Domain 10: Acquisition cost** |  |  |  |
| Monetary cost of the preventive measure | 4.2 ± 0.8 (4.0) [3 to 5] | 4.0 ± 1.2 (4.0) [3 to 5] | 4.4 ± 0.5 (4.0) [4 to 5] |
| **Domain 11: Impact on other costs** |  |  |  |
| Cost of the disease on the health system (excludes acquisition cost) | 3.9 ± 1.1 (4.0) [2 to 5] | 3.5 ± 1.3 (4.0) [2 to 5] | 4.2 ± 0.8 (4.0) [3 to 5] |
| Productivity cost: absenteeism | 2.6 ± 0.9 (3.0) [1 to 4] | 2.5 ± 1.3 (3.0) [1 to 4] | 2.6 ± 0.5 (3.0) [2 to 3] |
| Cost of the disease on the patient (out-of-pocket expenses) | 2.7 ± 0.5 (3.0) [2 to 3] | 2.8 ± 0.5 (3.0) [2 to 3] | 2.6 ± 0.5 (3.0) [2 to 3] |
| **Domain 12: Social benefits** |  |  |  |
| Impact on health inequity | 3.3 ± 1.2 (4.0) [1 to 5] | 3.3 ± 1.7 (4.0) [1 to 5] | 3.4 ± 0.9 (4.0) [2 to 4] |
| Public health awareness (including antibiotic resistance) | 2.7 ± 1.1 (3.0) [1 to 4] | 2.8 ± 1.3 (3.0) [1 to 4] | 2.6 ± 1.1 (3.0) [1 to 4] |
| Innovation stimulus | 2.3 ± 1.1 (3.0) [1 to 4] | 2.3 ± 1.0 (3.0) [1 to 3] | 2.4 ± 1.3 (3.0) [1 to 4] |

*Clinicians*: two pediatrists, one doctor specialist in preventive medicine and public health, one epidemiologist, one clinical virologist. *Managers*: one former director of the Common Portfolio of Services from the NHS and Pharmacy, one manager of a health research institute, two directors of health departments, one health economist.

**Table (S6).2: Scores, overall and by subgroup, nirsevimab vs. placebo**

Mean ± standard deviation (median) [min to max]

| Criteria | Overall (n=9) | Clinicians (n=4) | Managers (n=5) |
| --- | --- | --- | --- |
| **Domain 1: Severity of disease** |  |  |  |
| Severity of symptoms | 3.0 ± 1.1 (3.0) [1 to 5] | 2.5 ± 1.0 (3.0) [1 to 3] | 3.4 ± 1.1 (3.0) [2 to 5] |
| Lethality risk | 2.1 ± 1.2 (2.0) [1 to 4] | 2.3 ± 1.5 (2.0) [1 to 4] | 2.0 ± 1.0 (2.0) [1 to 3] |
| Comorbidity risk | 3.6 ± 0.9 (4.0) [2 to 5] | 3.3 ± 1.0 (3.5) [2 to 4] | 3.8 ± 0.8 (4.0) [3 to 5] |
| **Domain 2: Burden of disease** |  |  |  |
| Incidence of RSV cases | 4.0 ± 0.7 (4.0) [3 to 5] | 3.5 ± 0.6 (3.5) [3 to 4] | 4.4 ± 0.5 (4.0) [4 to 5] |
| Incidence on the outpatient setting | 4.1 ± 0.8 (4.0) [3 to 5] | 3.8 ± 1.0 (3.5) [3 to 5] | 4.4 ± 0.5 (4.0) [4 to 5] |
| Incidence on the inpatient setting | 3.3 ± 1.3 (4.0) [1 to 5] | 3.5 ± 1.7 (4.0) [1 to 5] | 3.2 ± 1.1 (4.0) [2 to 4] |
| Time of duration of acute symptoms | 2.7 ± 1.0 (2.0) [2 to 5] | 3.0 ± 1.4 (2.5) [2 to 5] | 2.4 ± 0.5 (2.0) [2 to 3] |
| **Domain 3: Prevention or Treatment Alternatives** |  |  |  |
| Prevention alternatives | 4.2 ± 0.7 (4.0) [3 to 5] | 4.0 ± 0.8 (4.0) [3 to 5] | 4.4 ± 0.5 (4.0) [4 to 5] |
| Availability of treatment | 4.4 ± 0.7 (5.0) [3 to 5] | 4.3 ± 1.0 (4.5) [3 to 5] | 4.6 ± 0.5 (5.0) [4 to 5] |
| **Domain 4: Size of population** |  |  |  |
| Population in which the prevention strategy would be indicated | 4.2 ± 1.6 (5.0) [0 to 5] | 3.8 ± 2.5 (5.0) [0 to 5] | 4.6 ± 0.5 (5.0) [4 to 5] |
| **Domain 5: Efficacy** |  |  |  |
| Efficacy of the preventive measure | 4.3 ± 0.5 (4.0) [4 to 5] | 4.3 ± 0.5 (4.0) [4 to 5] | 4.4 ± 0.5 (4.0) [4 to 5] |
| **Domain 6: Population protection** |  |  |  |
| Group immunity (collective protection) | 1.1 ± 1.2 (1.0) [0 to 3] | 1.0 ± 1.2 (1.0) [0 to 2] | 1.2 ± 1.3 (1.0) [0 to 3] |
| Transmissibility | 3.7 ± 1.0 (4.0) [2 to 5] | 3.5 ± 1.3 (3.5) [2 to 5] | 3.8 ± 0.8 (4.0) [3 to 5] |
| **Domain 7: safety** |  |  |  |
| Serious adverse events | -0.1 ± 0.3 (0.0) [-1 to 0] | 0.0 ± 0.0 (0.0) [0 to 0] | -0.2 ± 0.4 (0.0) [-1 to 0] |
| Mild adverse events | -0.7 ± 1.1 (0.0) [-3 to 0] | -1.0 ± 1.4 (-0.5) [-3 to 0] | -0.4 ± 0.9 (0.0) [-2 to 0] |
| **Domain 8: Quality of evidence** |  |  |  |
| Certainty about the efficacy of the preventive measure | 3.9 ± 0.3 (4.0) [3 to 4] | 3.8 ± 0.5 (4.0) [3 to 4] | 4.0 ± 0.0 (4.0) [4 to 4] |
| **Domain 9: Impact on quality of life** |  |  |  |
| Impact on the population of children | 4.2 ± 1.0 (5.0) [3 to 5] | 4.0 ± 1.2 (4.0) [3 to 5] | 4.4 ± 0.9 (5.0) [3 to 5] |
| Impact on the population over 65 years of age | 2.6 ± 1.2 (3.0) [1 to 5] | 2.3 ± 1.0 (2.5) [1 to 3] | 2.8 ± 1.5 (3.0) [1 to 5] |
| Impact on caregivers | 3.6 ± 1.1 (4.0) [2 to 5] | 3.5 ± 1.3 (3.5) [2 to 5] | 3.6 ± 1.1 (4.0) [2 to 5] |
| **Domain 10: Acquisition cost** |  |  |  |
| Monetary cost of the preventive measure | -3.7 ± 1.8 (-5.0) [-5 to 0] | -3.3 ± 2.4 (-4.0) [-5 to 0] | -4.0 ± 1.4 (-5.0) [-5 to 2] |
| **Domain 11: Impact on other costs** |  |  |  |
| Cost of the disease on the health system (excludes acquisition cost) | 3.3 ± 1.5 (3.0) [0 to 5] | 3.0 ± 2.2 (3.5) [0 to 5] | 3.6 ± 0.9 (3.0) [3 to 5] |
| Productivity cost: absenteeism | 2.6 ± 1.4 (2.0) [0 to 5] | 1.5 ± 1.0 (2.0) [0 to 2] | 3.4 ± 1.1 (3.0) [2 to 5] |
| Cost of the disease on the patient (out-of-pocket expenses) | 1.8 ± 1.8 (2.0) [-1 to 5] | 0.8 ± 1.7 (0.5) [-1 to 3] | 2.6 ± 1.5 (2.0) [1 to 5] |
| **Domain 12: Social benefits** |  |  |  |
| Impact on health inequity | 2.8 ± 1.6 (3.0) [0 to 5] | 2.8 ± 1.9 (3.5) [0 to 4] | 2.8 ± 1.6 (2.0) [1 to 5] |
| Public health awareness (including antibiotic resistance) | 2.3 ± 1.6 (3.0) [0 to 5] | 2.0 ± 1.4 (2.5) [0 to 3] | 2.6 ± 1.8 (3.0) [0 to 5] |
| Innovation stimulus | 3.6 ± 1.4 (4.0) [1 to 5] | 3.8 ± 1.9 (4.5) [1 to 5] | 3.4 ± 1.1 (3.0) [2 to 5] |

*Clinicians*: two pediatrists, one doctor specialist in preventive medicine and public health, one epidemiologist, one clinical virologist. *Managers*: one former director of the Common Portfolio of Services from the NHS and Pharmacy, one manager of a health research institute, two directors of health departments, one health economist.

**Table (S6).3: Value estimates, overall and by subgroup, nirsevimab vs. placebo**

Mean ± standard deviation [min to max]

| Criteria | Overall (n=9) | Clinicians (n=4) | Managers (n=5) |
| --- | --- | --- | --- |
| **Domain 1: Severity of disease** |  |  |  |
| Severity of symptoms | 0.03 ± 0.01 [0.01 to 0.04] | 0.02 ± 0.01 [0.01 to 0.03] | 0.03 ± 0.01 [0.01 to 0.04] |
| Lethality risk | 0.02 ± 0.01 [0.00 to 0.04] | 0.02 ± 0.02 [0.00 to 0.04] | 0.02 ± 0.01 [0.01 to 0.03] |
| Comorbidity risk | 0.03 ± 0.01 [0.02 to 0.04] | 0.03 ± 0.01 [0.02 to 0.03] | 0.03 ± 0.01 [0.02 to 0.04] |
| **Domain 2: Burden of disease** |  |  |  |
| Incidence of RSV cases | 0.03 ± 0.01 [0.02 to 0.05] | 0.03 ± 0.01 [0.02 to 0.04] | 0.04 ± 0.01 [0.03 to 0.05] |
| Incidence on the outpatient setting | 0.04 ± 0.01 [0.01 to 0.05] | 0.03 ± 0.02 [0.01 to 0.05] | 0.04 ± 0.01 [0.03 to 0.05] |
| Incidence on the inpatient setting | 0.03 ± 0.01 [0.01 to 0.06] | 0.03 ± 0.02 [0.01 to 0.06] | 0.03 ± 0.01 [0.01 to 0.04] |
| Time of duration of acute symptoms | 0.02 ± 0.01 [0.01 to 0.04] | 0.02 ± 0.01 [0.01 to 0.04] | 0.02 ± 0.01 [0.01 to 0.02] |
| **Domain 3: Prevention or Treatment Alternatives** |  |  |  |
| Prevention alternatives | 0.03 ± 0.01 [0.02 to 0.05] | 0.03 ± 0.01 [0.03 to 0.04] | 0.03 ± 0.01 [0.02 to 0.05] |
| Availability of treatment | 0.04 ± 0.02 [0.01 to 0.06] | 0.04 ± 0.02 [0.01 to 0.06] | 0.04 ± 0.01 [0.02 to 0.05] |
| **Domain 4: Size of population** |  |  |  |
| Population in which the prevention strategy would be indicated | 0.03 ± 0.02 [0.00 to 0.05] | 0.02 ± 0.02 [0.00 to 0.04] | 0.04 ± 0.01 [0.03 to 0.05] |
| **Domain 5: Efficacy** |  |  |  |
| Efficacy of the preventive measure | 0.04 ± 0.01 [0.03 to 0.05] | 0.04 ± 0.01 [0.04 to 0.05] | 0.04 ± 0.01 [0.03 to 0.05] |
| **Domain 6: Population protection** |  |  |  |
| Group immunity (collective protection) | 0.01 ± 0.01 [0.00 to 0.03] | 0.01 ± 0.01 [0.00 to 0.01] | 0.01 ± 0.01 [0.00 to 0.03] |
| Transmissibility | 0.03 ± 0.01 [0.01 to 0.05] | 0.03 ± 0.01 [0.01 to 0.04] | 0.03 ± 0.01 [0.02 to 0.05] |
| **Domain 7: safety** |  |  |  |
| Serious adverse events | 0.00 ± 0.00 [-0.01 to 0.00] | 0.00 ± 0.00 [0.00 to 0.00] | 0.00 ± 0.00 [-0.01 to 0.00] |
| Mild adverse events | 0.00 ± 0.01 [-0.02 to 0.00] | 0.00 ± 0.01 [-0.01 to 0.00] | 0.00 ± 0.01 [-0.02 to 0.00] |
| **Domain 8: Quality of evidence** |  |  |  |
| Certainty about the efficacy of the preventive measure | 0.03 ± 0.01 [0.02 to 0.05] | 0.04 ± 0.01 [0.02 to 0.05] | 0.03 ± 0.01 [0.03 to 0.04] |
| **Domain 9: Impact on quality of life** |  |  |  |
| Impact on the population of children | 0.04 ± 0.01 [0.02 to 0.05] | 0.03 ± 0.01 [0.02 to 0.04] | 0.04 ± 0.01 [0.02 to 0.05] |
| Impact on the population over 65 years of age | 0.02 ± 0.01 [0.01 to 0.04] | 0.02 ± 0.01 [0.01 to 0.02] | 0.02 ± 0.01 [0.01 to 0.04] |
| Impact on caregivers | 0.02 ± 0.01 [0.01 to 0.04] | 0.02 ± 0.01 [0.01 to 0.04] | 0.02 ± 0.01 [0.01 to 0.03] |
| **Domain 10: Acquisition cost** |  |  |  |
| Monetary cost of the preventive measure | -0.03 ± 0.02 [-0.05 to 0.00] | -0.03 ± 0.02 [-0.05 to 0.00] | -0.04 ± 0.02 [-0.05 to -0.02] |
| **Domain 11: Impact on other costs** |  |  |  |
| Cost of the disease on the health system (excludes acquisition cost) | 0.03 ± 0.02 [0.00 to 0.05] | 0.03 ± 0.02 [0.00 to 0.05] | 0.03 ± 0.01 [0.02 to 0.05] |
| Productivity cost: absenteeism | 0.01 ± 0.01 [0.00 to 0.02] | 0.01 ± 0.01 [0.00 to 0.02] | 0.02 ± 0.01 [0.01 to 0.02] |
| Cost of the disease on the patient (out-of-pocket expenses) | 0.01 ± 0.01 [0.00 to 0.02] | 0.01 ± 0.01 [0.00 to 0.02] | 0.01 ± 0.01 [0.01 to 0.02] |
| **Domain 12: Social benefits** |  |  |  |
| Impact on health inequity | 0.02 ± 0.01 [0.00 to 0.04] | 0.02 ± 0.02 [0.00 to 0.04] | 0.02 ± 0.01 [0.00 to 0.03] |
| Public health awareness (including antibiotic resistance) | 0.01 ± 0.01 [0.00 to 0.02] | 0.01 ± 0.01 [0.00 to 0.02] | 0.01 ± 0.01 [0.00 to 0.02] |
| Innovation stimulus | 0.02 ± 0.01 [0.00 to 0.03] | 0.02 ± 0.01 [0.00 to 0.03] | 0.02 ± 0.01 [0.00 to 0.03] |
| **Total** | **0.56 ± 0.11 [0.32 to 0.67]** | **0.54 ± 0.16 [0.32 to 0.67]** | **0.57 ± 0.05 [0.51 to 0.63]** |

*Clinicians*: two pediatrists, one doctor specialist in preventive medicine and public health, one epidemiologist, one clinical virologist. *Managers*: one former director of the Common Portfolio of Services from the NHS and Pharmacy, one manager of a health research institute, two directors of health departments, one health economist.
