# Supplementary material for: Evaluation of antibody-based preventive alternatives for respiratory syncytial virus: a novel multi-criteria decision analysis framework and assessment of nirsevimab in Spain
Source: BMC Infect Dis. 2024 Jan 18;24:99. doi: 10.1186/s12879-024-08988-9 (PMC10797756; doi:10.1186/s12879-024-08988-9)
Supplement: Supplementary file 6 — Supplementary Material 6: Detailed scores per criterion [file 12879_2024_8988_MOESM6_ESM.docx]

**Evaluation of Antibody-based Preventive Alternatives for Respiratory Syncytial Virus: A Novel Multi-Criteria Decision Analysis Framework and Assessment of Nirsevimab in Spain**

**Authors**: Jorge Mestre-Ferrándiz^1^, Agustín Rivero^2^, Alejandro Orrico-Sánchez^3,4,5^, Álvaro Hidalgo^6,7^, Fernando Abdalla^8^, Isabel Martín^9^, Javier Álvarez^10^, Manuel García-Cenoz^11^, Maria del Carmen Pacheco^12^, María Garcés-Sánchez^13^, Néboa Zozaya^8,14^, Raúl Ortiz-de-Lejarazu^15^

**Affiliations**: ^1^Department of Economics, University Carlos III, Madrid, Spain; ^2^Department of Management, Bioregión de Salud y Bienestar (BioMad), Madrid, Spain; ^3^Department of Vaccines Research, Fundación Para el Fomento de la Investigación Sanitaria y Biomédica de la Comunitat Valenciana (Fisabio), Valencia, Spain; ^4^Catholic University of Valencia, Spain; ^5^Centro de Investigación Biomédica en Red de Epidemiología y Salud Pública (CIBERESP); ^6^Weber Foundation, Madrid, Spain; ^7^Department of Economic Analysis and Finances, University of Castilla-La Mancha. Toledo, Spain; ^8^Department of Health Affairs and Policy Research, Vivactis Weber, Madrid, Spain; ^9^Department of Primary Care, Rochapea Healthcare Center, Navarra, Spain; ^10^Department of Pediatrics, Hospital Costa del Sol, Málaga, Spain; ^11^Public Health Institute of Navarra, Navarra, Spain; ^12^Department of Epidemiology, General Directorate of Public Health, Castilla y León, Spain; ^13^Department of Pediatrics, Nazaret Healthcare Center, Valencia, Spain; ^14^Department of Quantitative Methods in Economics and Management, University Las Palmas de Gran Canaria. Las Palmas, Spain; ^15^National Influenza Centre, Scientific Advisor and Emeritus Director, School of Medicine, University of Valladolid, Castilla y León, Spain.

**SUPPLEMENTARY FILE 6: DETAILED SCORES PER CRITERION**

***Box and whisker plot and comments from the multidisciplinary committee of experts***

# Index of Figures

[**Figure (S7).1. Global score, Severity of symptoms** 4](#_Toc116394992)

[**Figure (S7).2. Overall score, Lethality risk** 5](#_Toc116394993)

[**Figure (S7).3. Global score, Comorbidity risk** 6](#_Toc116394994)

[**Figure (S7).4. Overall score, Incidence of RSV cases** 7](#_Toc116394995)

[**Figure (S7).5. Global score, Incidence on the outpatient setting** 8](#_Toc116394996)

[**Figure (S7).6. Overall score, Incidence on the inpatient setting** 9](#_Toc116394997)

[**Figure (S7).7. Global score, Time of duration of acute symptoms** 10](#_Toc116394998)

[**Figure (S7).8. Overall score, Prevention alternatives** 11](#_Toc116394999)

[**Figure (S7).9. Overall score, Availability of treatment** 12](#_Toc116395000)

[**Figure (S7).10. Overall score, Population in which the prevention strategy would be indicated** 13](#_Toc116395001)

[**Figure (S7).11. Overall score, Efficacy of the preventive measure** 14](#_Toc116395002)

[**Figure (S7).12. Overall score, Group immunity (collective protection)** 15](#_Toc116395003)

[**Figure (S7).13. Overall score, Transmissibility** 16](#_Toc116395004)

[**Figure (S7).14. Overall score, Serious adverse events** 17](#_Toc116395005)

[**Figure (S7).15. Overall score, Mild adverse events** 18](#_Toc116395006)

[**Figure (S7).16. Overall score, Certainty about the efficacy of the preventive measure** 19](#_Toc116395007)

[**Figure (S7).17. Overall score, Impact on the population of children** 20](#_Toc116395008)

[**Figure (S7).18. Overall score, Impact on the population over 65 years of age** 21](#_Toc116395009)

[**Figure (S7).19. Overall score, Impact on caregivers** 22](#_Toc116395010)

[**Figure (S7).20. Overall score, Monetary cost of the preventive measure** 23](#_Toc116395011)

[**Figure (S7).21. Overall score, Cost of the disease on the health system (excludes acquisition cost)** 24](#_Toc116395012)

[**Figure (S7).22. Overall score, Productivity cost: absenteeism** 25](#_Toc116395013)

[**Figure (S7).23. Overall score, Cost of the disease on the patient (out-of-pocket expenses)** 26](#_Toc116395014)

[**Figure (S7).24. Overall score, Impact on health inequity** 27](#_Toc116395015)

[**Figure (S7).25. Overall score, Public health awareness (including antibiotic resistance)** 28](#_Toc116395016)

[**Figure (S7).26. Overall score, Innovation stimulus** 29](#_Toc116395017)

# Scores

The results of the scores given by the experts to each of the 26 criteria of this MCDA are presented below. The chosen form of visualization is the box-and-whisker plot, which visually represents various descriptive statistics. The **lower and upper limits** of the graph represent the minimum and maximum scores. The **box** represents the central quartile of the scores given, as it is bounded by the lower (25%) and upper (75%) quartile. The **"x" represents** the mean value of the scores, and the **line dividing the boxes** represents the median (in some cases, the median coincides with one of the two quartiles represented graphically). The **circles** represent the extreme or marginal cases (*outliers*), which show scores that are more than 1.5 times away from the interquartile ranges. Finally, **larger distances between the extremes of the** box and the graph represent larger dispersions in the responses.

## *1. Severity of symptoms*

| **Figure (S7).1. Global score, Severity of symptoms**  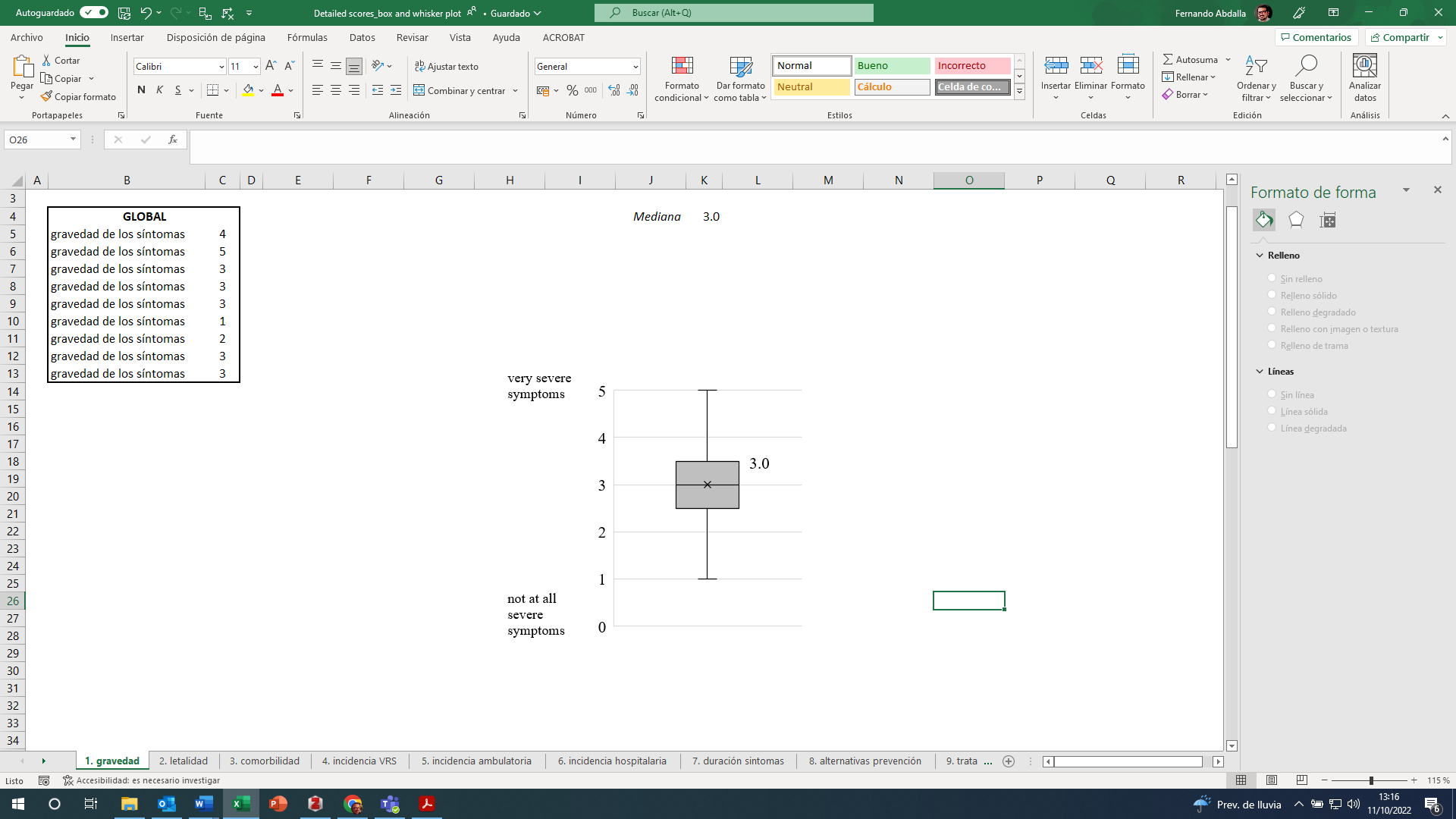 |
| --- |

The overall mean score (n=9) for the *severity of symptoms* criterion was 3.0 ± 1.1 out of 5.0 (median: 3.0), reflecting a disease with moderate symptoms. There was little variability between responses, with the vast majority of scores falling between 2 and 4.

The experts who scored the highest considered the severity of symptoms in those patients admitted to hospital, while others focused their scoring on the severity of symptoms in the outpatient setting (including the stress on children and caregivers). Moreover, some committee members considered only children who have symptoms, excluding symptom-free infected children from their mental algorithm. They commented that, in cases with symptoms, RSV is one of the most serious pathologies in pediatric care. On the other hand, those who scored lower made the observation that RSV is a less serious disease than others, such as ovarian or pancreatic cancer.

## *2. Lethality risk*

| **Figure (S7).2. Overall score, Lethality risk**  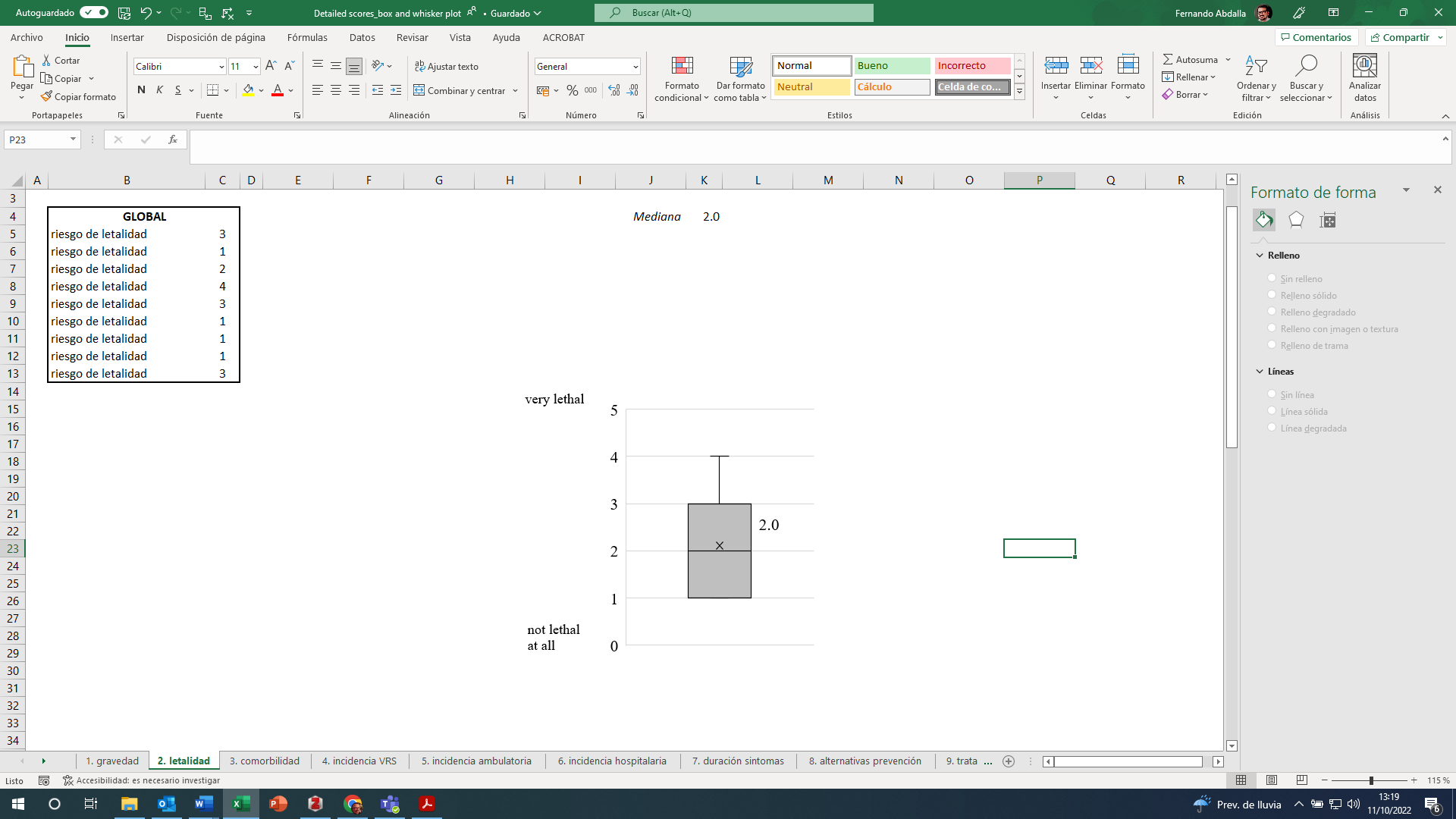 |
| --- |

The overall mean score (n=9) for the *lethality risk* criterion was 2.1 ± 1.2 (median: 2.0), reflecting that RSV is not very lethal. There was little variability among responses, with the vast majority of scores falling between 1 and 3.

For the experts, this was an expected outcome, based on the evidence presented, with a death rate for children ≤2 years of 82/100,000.

## *3. Comorbidity risk*

| **Figure (S7).3. Global score, Comorbidity risk**  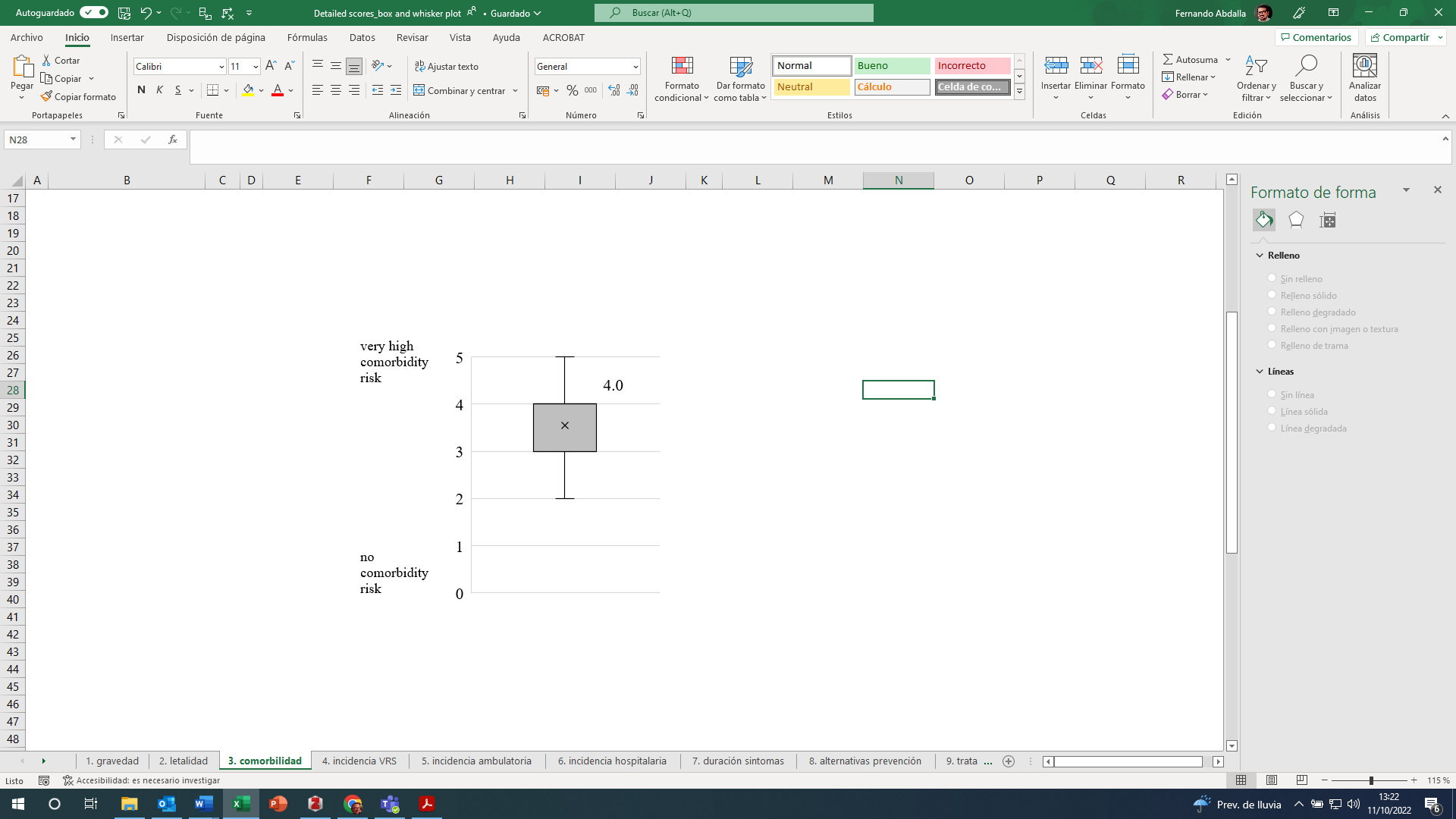 |
| --- |

The overall mean score (n=9) for the *comorbidity risk* criterion was 3.6 ± 0.9 (median: 4.0), reflecting that RSV-infected children are at high risk of developing comorbidities (short, medium, and long-term). There was little variability among responses, with the vast majority of scores falling between 3 and 4.

Some experts have scored based on long-term symptoms (asthma, etc.). Others commented that, according to several ongoing studies, there is some evidence that in the long term the risk of asthma exists in children hospitalized for RSV in the first months of life, however, there is stronger evidence that the risk is the same, or even higher, when they are hospitalized for other respiratory viruses. They indicated that, because of this, it is often thought that it is not the pathogen itself that causes these comorbidities, but the susceptibility of the subject who requires hospitalization at a very early age due to the severity of the clinical profile, which predisposes to long-term asthma.

## *4. Incidence of RSV cases*

| **Figure (S7).4. Overall score, Incidence of RSV cases**  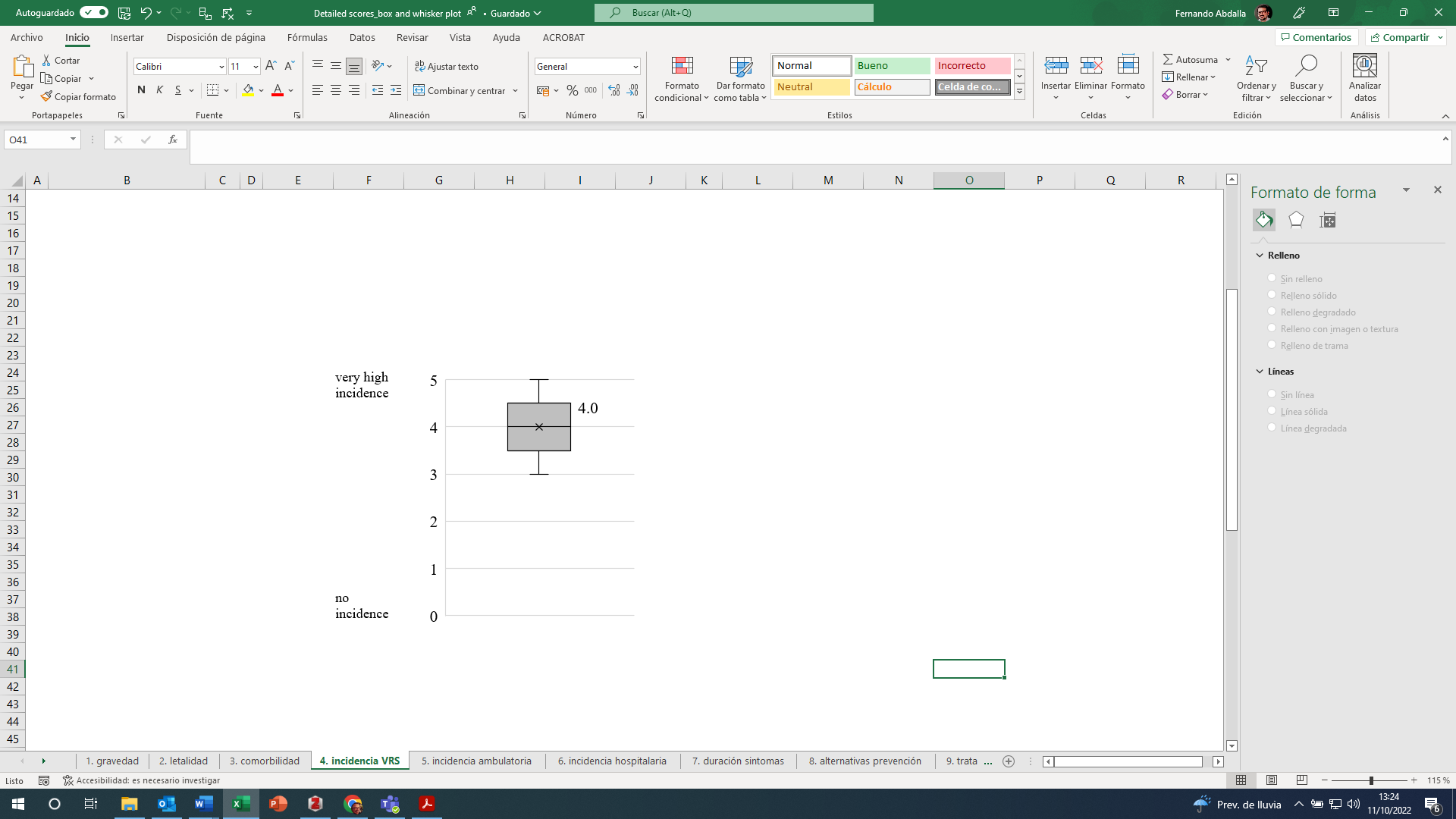 |
| --- |

The overall mean score (n=9) for the *incidence of RSV cases* criterion was 4.0 ± 0.7 (median: 4.0), reflecting a high/very high incidence of RSV cases. There was little variability between responses, with the vast majority of scores falling between 4 and 5.

The experts commented that this was an expected result, given the evidence presented, highlighting that approximately 65% of children ≤12 months and 90% of children <2 years become infected with RSV.

## *5. Incidence on the outpatient setting*

| **Figure (S7).5. Global score, Incidence on the outpatient setting**  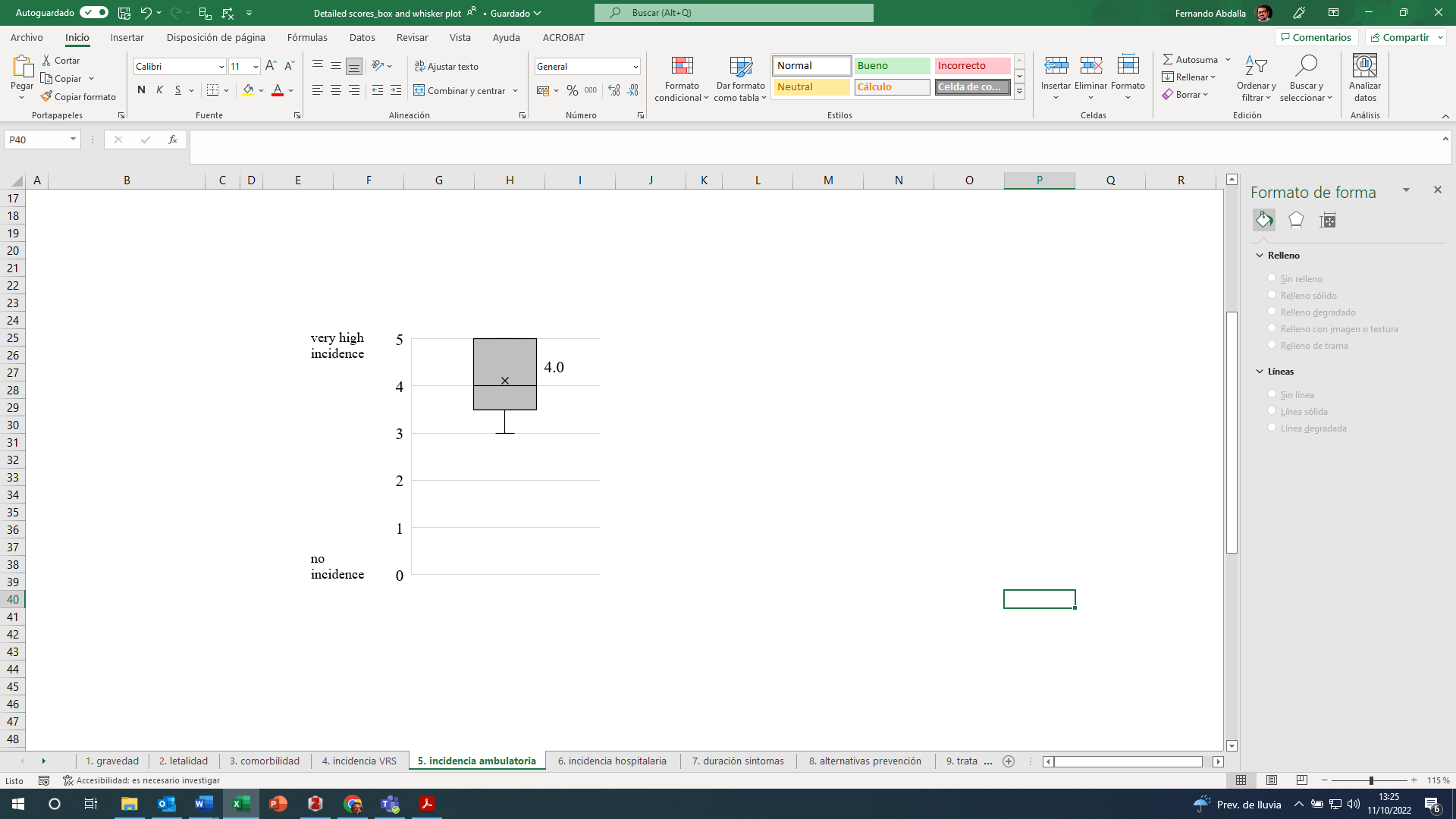 |
| --- |

The overall mean score (n=9) for the *incidence on the outpatient setting* criterion was 4.1 ± 0.8 (median: 4.0), reflecting a high/very high outpatient incidence of RSV cases. There was little variability among responses, with the vast majority of scores falling between 4 and 5.

Experts commented that this was an expected outcome, based on incidence rates of 39,690/100,000 children ≤12 months in primary care (averaging 9.0 visits per infected patient) and 882/100,000 in specialty care (averaging 2.0 visits per infected patient).

## *6. Incidence on the inpatient setting*

| **Figure (S7).6. Overall score, Incidence on the inpatient setting**  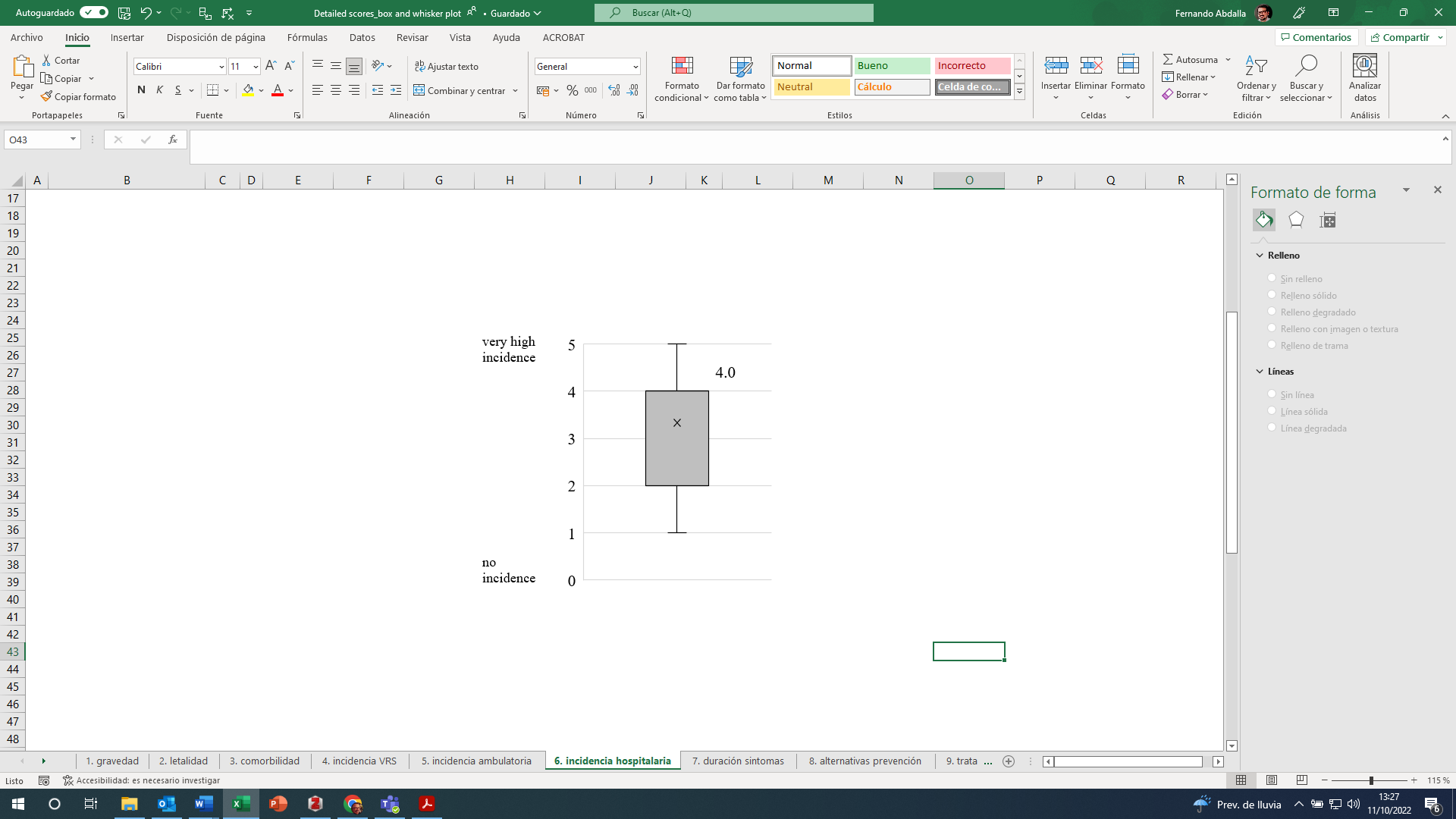 |
| --- |

The overall mean score (n=9) for the criterion *incidence on the inpatient setting* was 3.3 ± 1.3 (median: 4.0), reflecting a high incidence of RSV in the hospital setting. Five of the 9 experts scored 4, one expert scored 5, two experts scored 2, and one expert scored 1.

The experts who gave higher scores argued that children arriving on the inpatient setting are anticipating a serious admission. Other experts commented that the weight of the incidence on the inpatient setting is high, given that these are complicated cases to treat. Finally, some experts commented that this incidence is concentrated in certain months of the year (season), which makes the management of these admissions relatively more complicated than other types of illnesses. The experts who gave lower scores have used a logic based on incidence per 100,000 children, comparing it with other incidences (total and outpatient setting).

## *7. Time of duration of acute symptoms*

| **Figure (S7).7. Global score, Time of duration of acute symptoms**  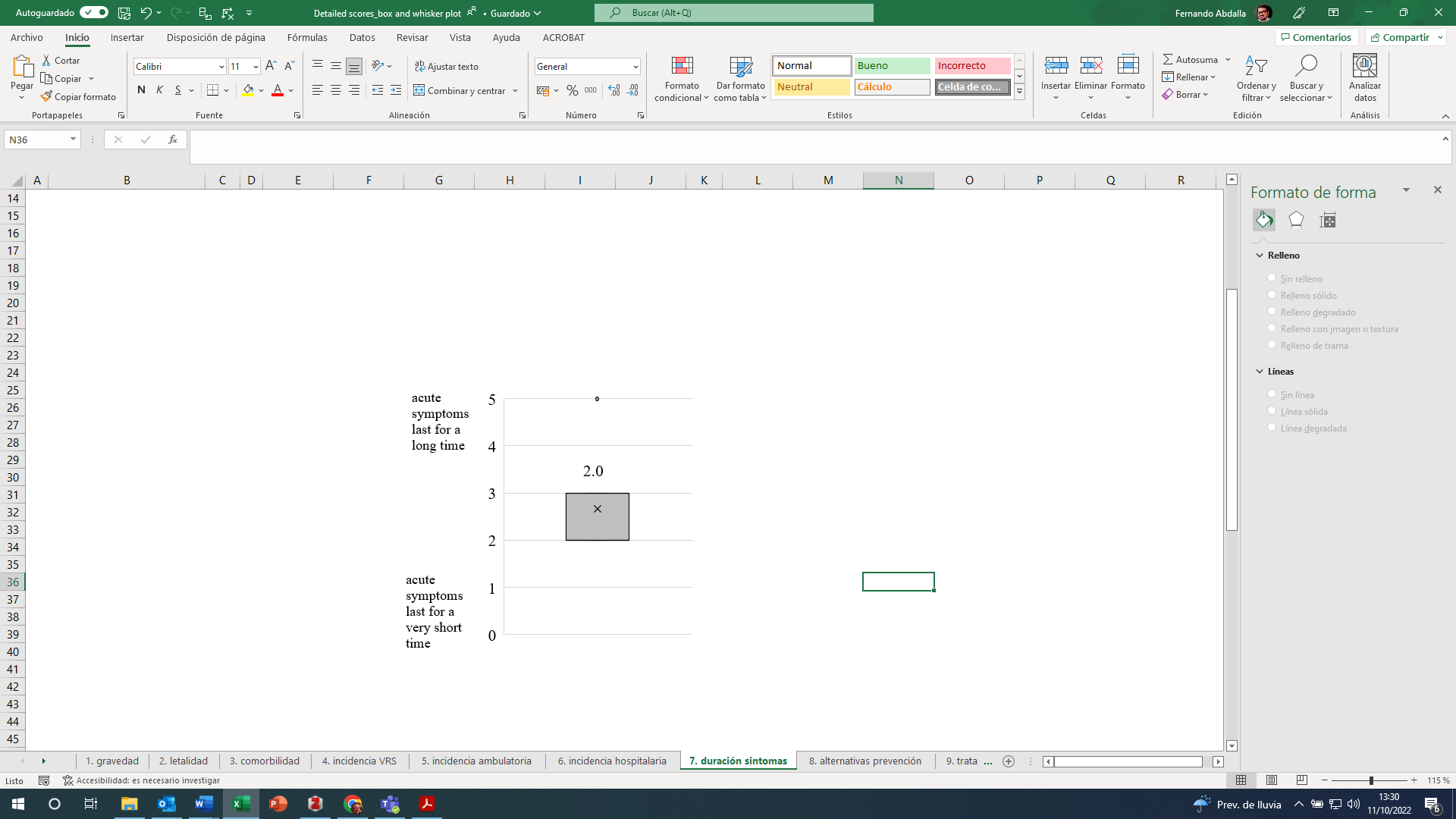 |
| --- |

The overall mean score (n=9) for the *time of duration of acute symptoms* criterion was 2.7 ± 1.0 (median: 2.0), reflecting the short duration of acute RSV symptoms. There was very little variability in responses, with the vast majority of experts scoring between 2 and 3, and one extreme case scoring 5.

The extreme case pointed out that a child with RSV bronchiolitis will continue to present clinical consequences of bronchial hyperresponsiveness for the rest of the season, and therefore the persistence of symptoms will be much greater than it seems. Some experts mention that this logic could also apply to the associated comorbidities criterion. For this reason, they scored based on the duration of acute symptoms. Other experts scored based on, among other aspects, the average hospital stay, which is less than 6 days (as a reference, they consider 8 days a relatively long stay).

## *8. Prevention alternatives*

| **Figure (S7).8. Overall score, Prevention alternatives**  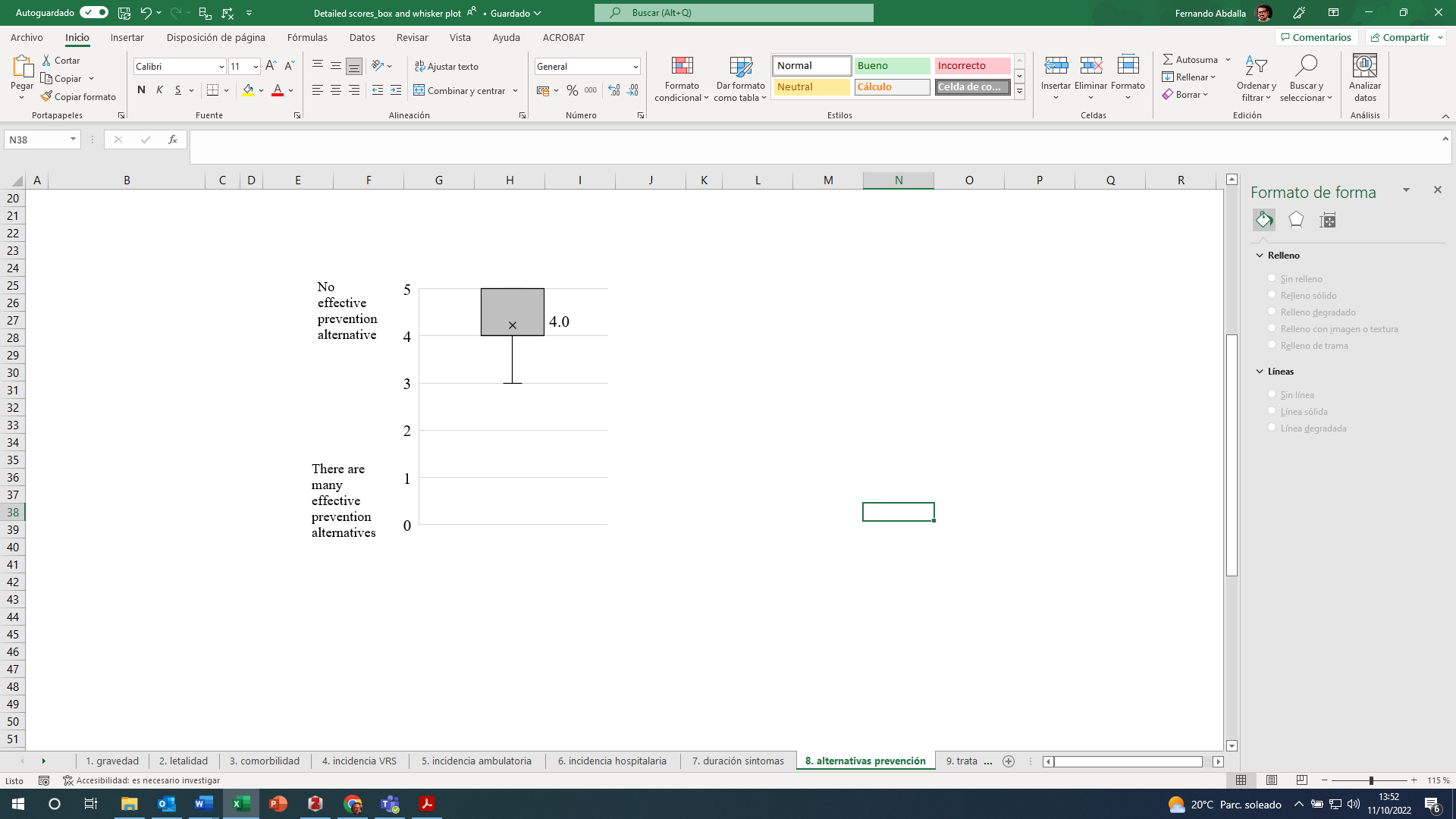 |
| --- |

The overall mean score (n=9) for the *prevention alternatives* criterion was 4.2 ± 0.7 (median: 4.0), reflecting that there are no effective prevention alternatives against RSV. There was very little variability in responses, with the vast majority of experts scoring between 4 and 5, and only one expert scoring 3.

The experts commented that this was an expected result, based on the evidence, which indicates that the only prevention alternative, palivizumab, is only indicated for a small group of children (infants born prematurely and/or with heart or lung disease) and on a inpatient setting.

## *9. Availability of treatment*

| **Figure (S7).9. Overall score, Availability of treatment**  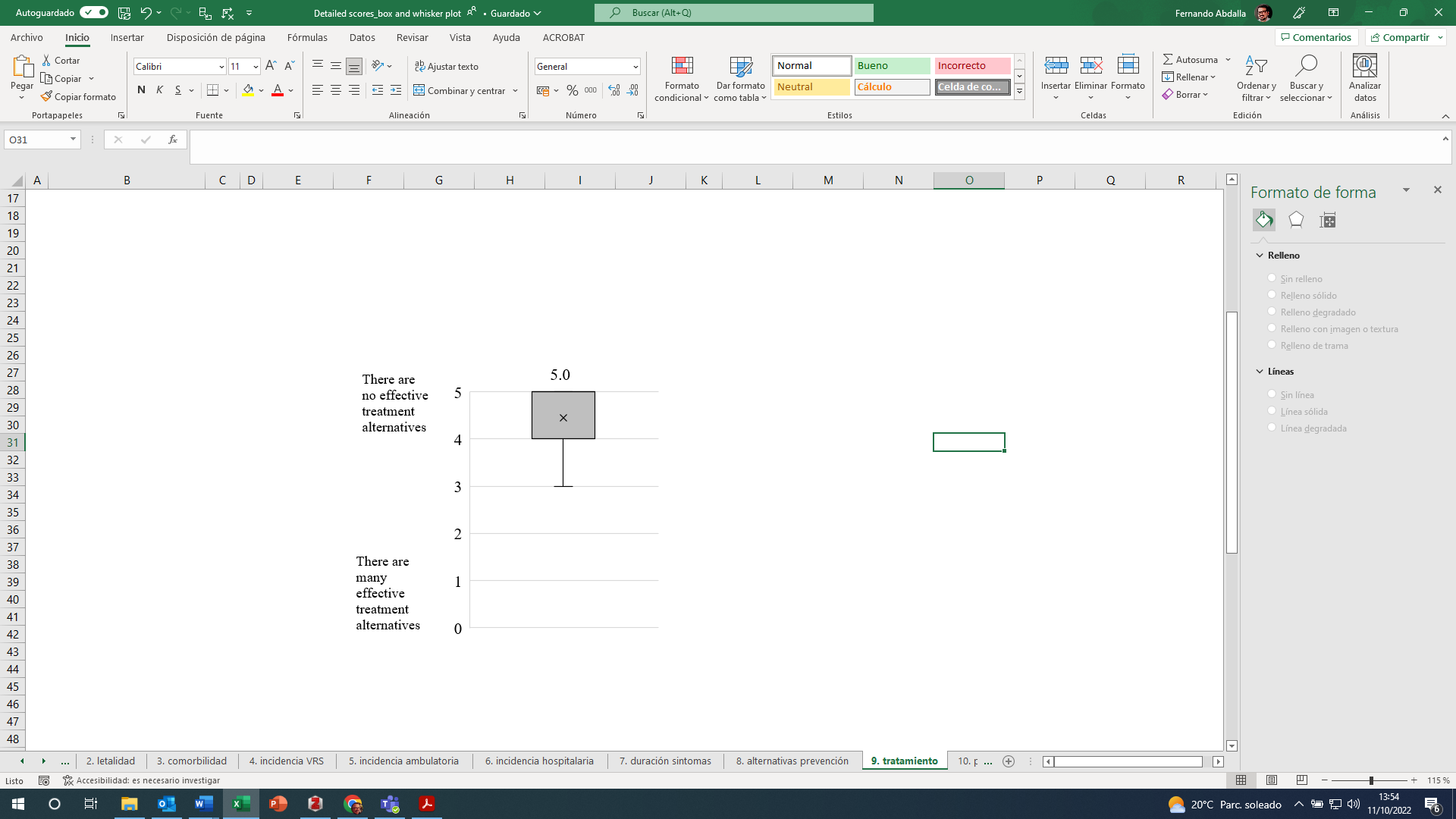 |
| --- |

The overall mean score (n=9) for the *availability of treatment* criterion was 4.4 ± 0.7 (median: 5.0), reflecting that there is no effective treatment alternative for RSV infection. There was very little variability in responses, with the vast majority of experts scoring between 4 and 5, and only one expert scoring 3.

Experts commented that this was an expected outcome, based on the evidence, which indicates that the only antiviral treatment (ribavirin) can only be given in severe cases, and its routine use is not recommended.

## *10. Population in which the prevention strategy would be indicated*

| **Figure (S7).10. Overall score, Population in which the prevention strategy would be indicated**  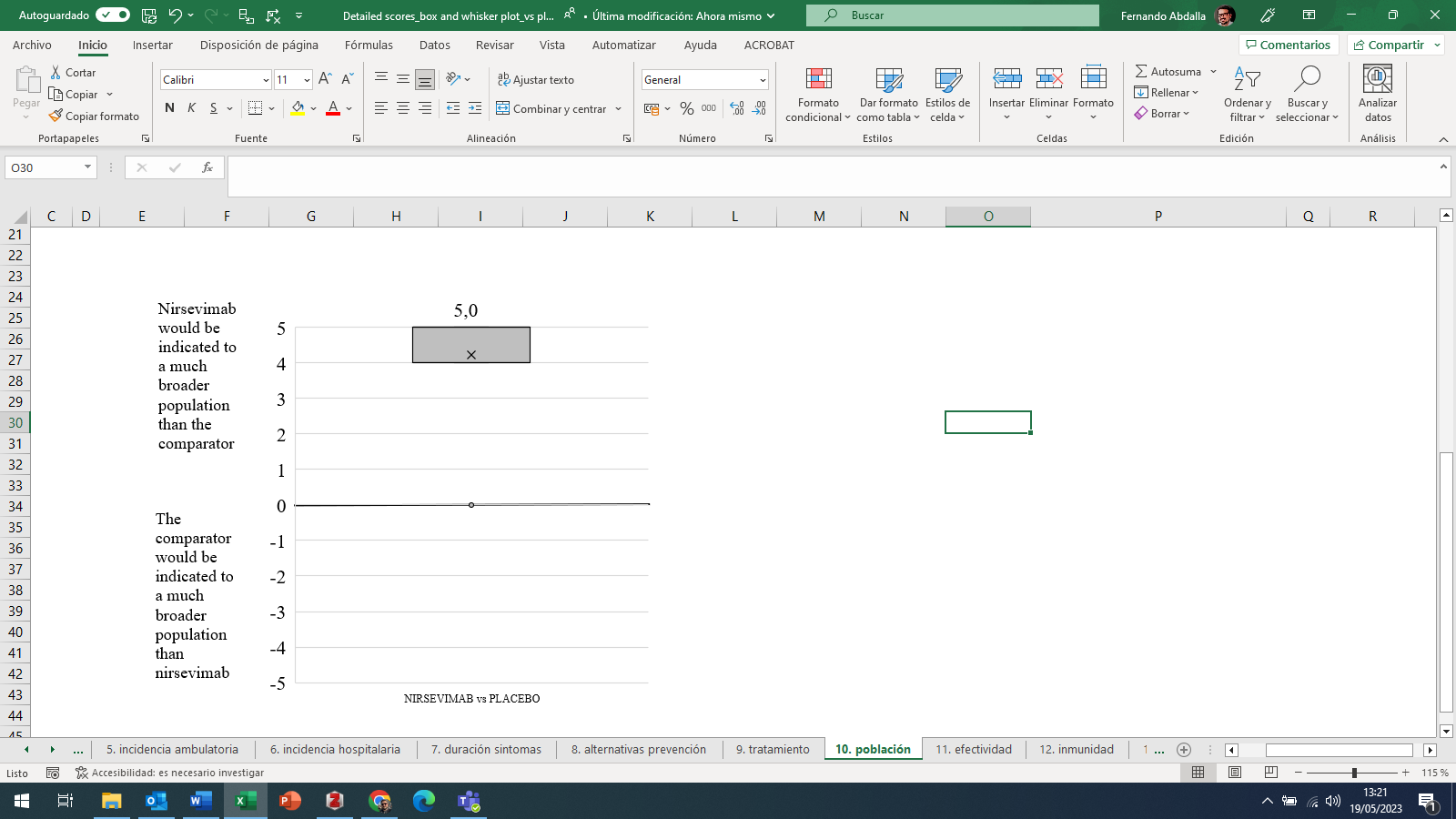 |
| --- |

The overall mean score (n=9) for the criterion *population in which the prevention strategy would be indicated* was 4.2±1.6, (median: 5.0), indicating that nirsevimab would be indicated for a much broader infant population than the comparator. In the comparison of nirsevimab versus placebo, six experts scored 5, two experts scored 4, and there was one extreme case who scored 0. The experts commented that this was an expected result, and reinforced the idea that placebo is neither an intervention nor a preventive measure.

## *11. Efficacy of the preventive measure*

| **Figure (S7).11. Overall score, Efficacy of the preventive measure**  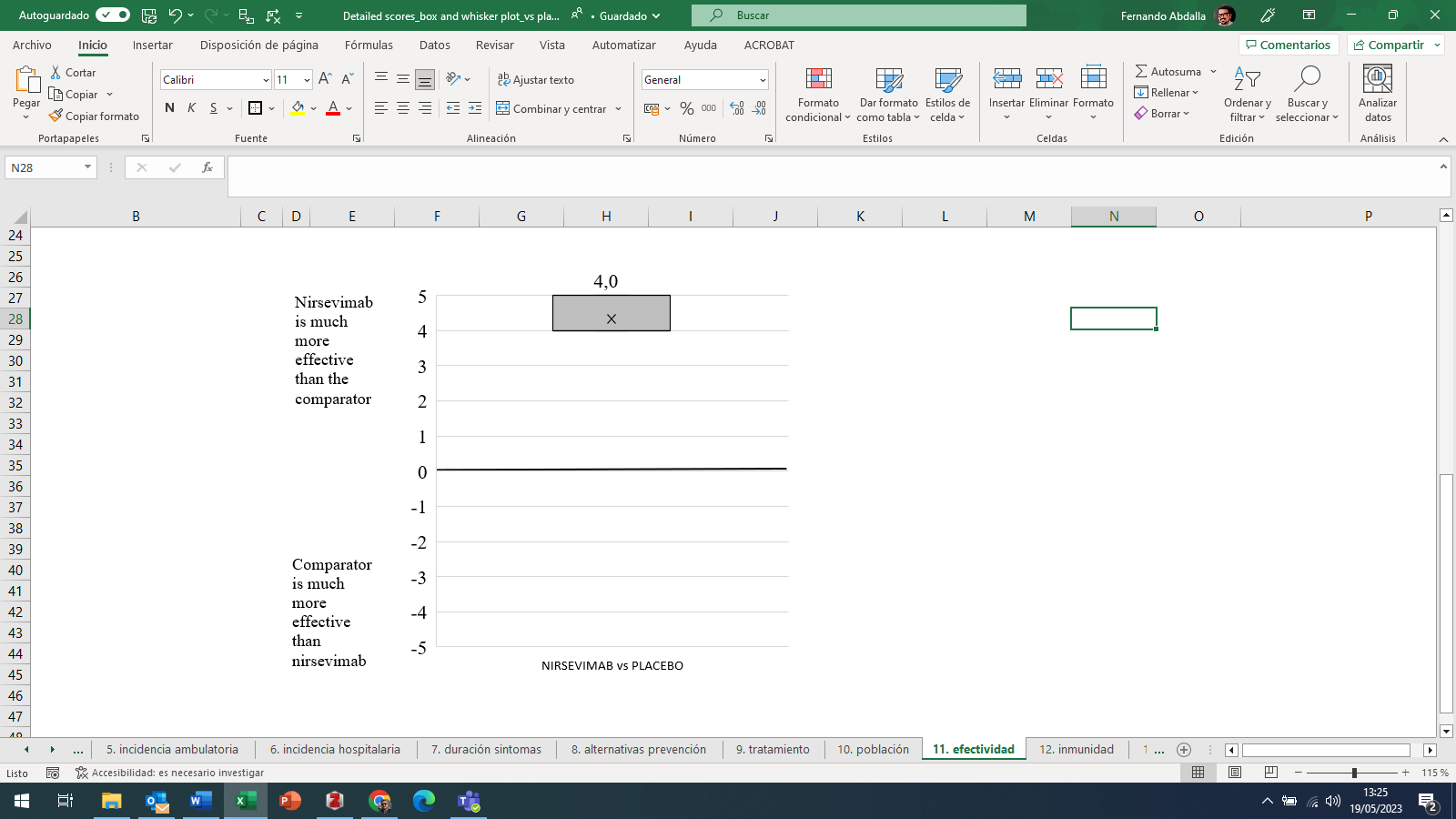 |
| --- |

The overall mean score (n=9) for the *efficacy of the preventive measure* criterion was 4.3 ± 0.5, (median: 4.0), where 5.0 means that nirsevimab is much more effective than the comparator, and -5.0 means that the comparator is much more effective than nirsevimab. There was very little variability in the comparison between nirsevimab and placebo (all experts scored between 4 and 5).

## *12. Group immunity (collective protection)*

| **Figure (S7).12. Overall score, Group immunity (collective protection)**  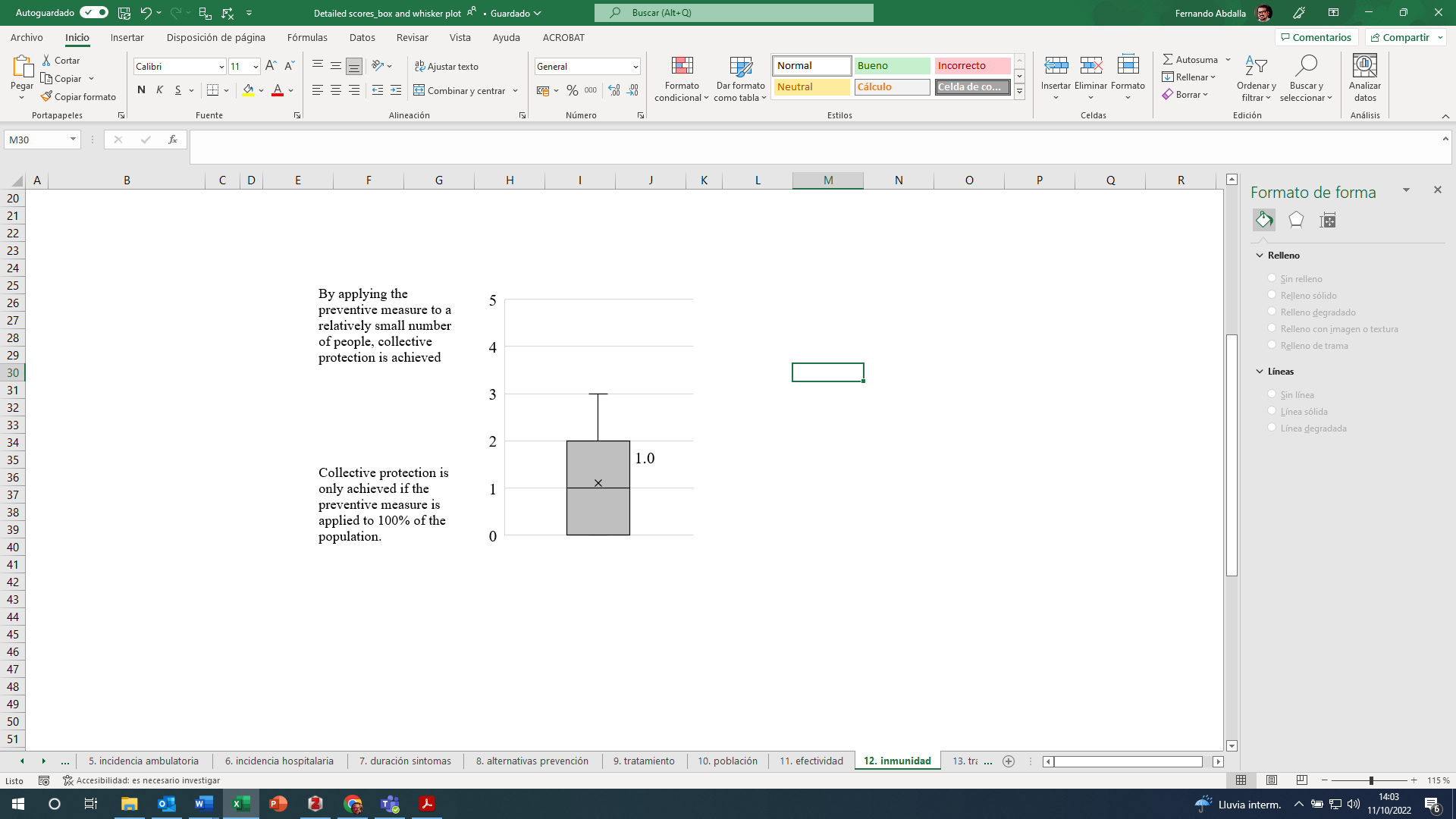 |
| --- |

The overall mean score (n=9) for the *group immunity (collective protection)* criterion was 1.1 ± 1.2 (median: 1.0), reflecting that collective protection would only be achieved if nirsevimab was applied to a very large share of the infant population. Only one expert scored 3, and all others scored between 0 and 2.

The experts commented that this was an expected result, especially based on the lack of evidence on this criterion, indicating that the collective protective effect of nirsevimab will only really be known once it is implemented in routine clinical practice and there is still additional reasonable doubt.

## *13. Transmissibility*

| **Figure (S7).13. Overall score, Transmissibility**  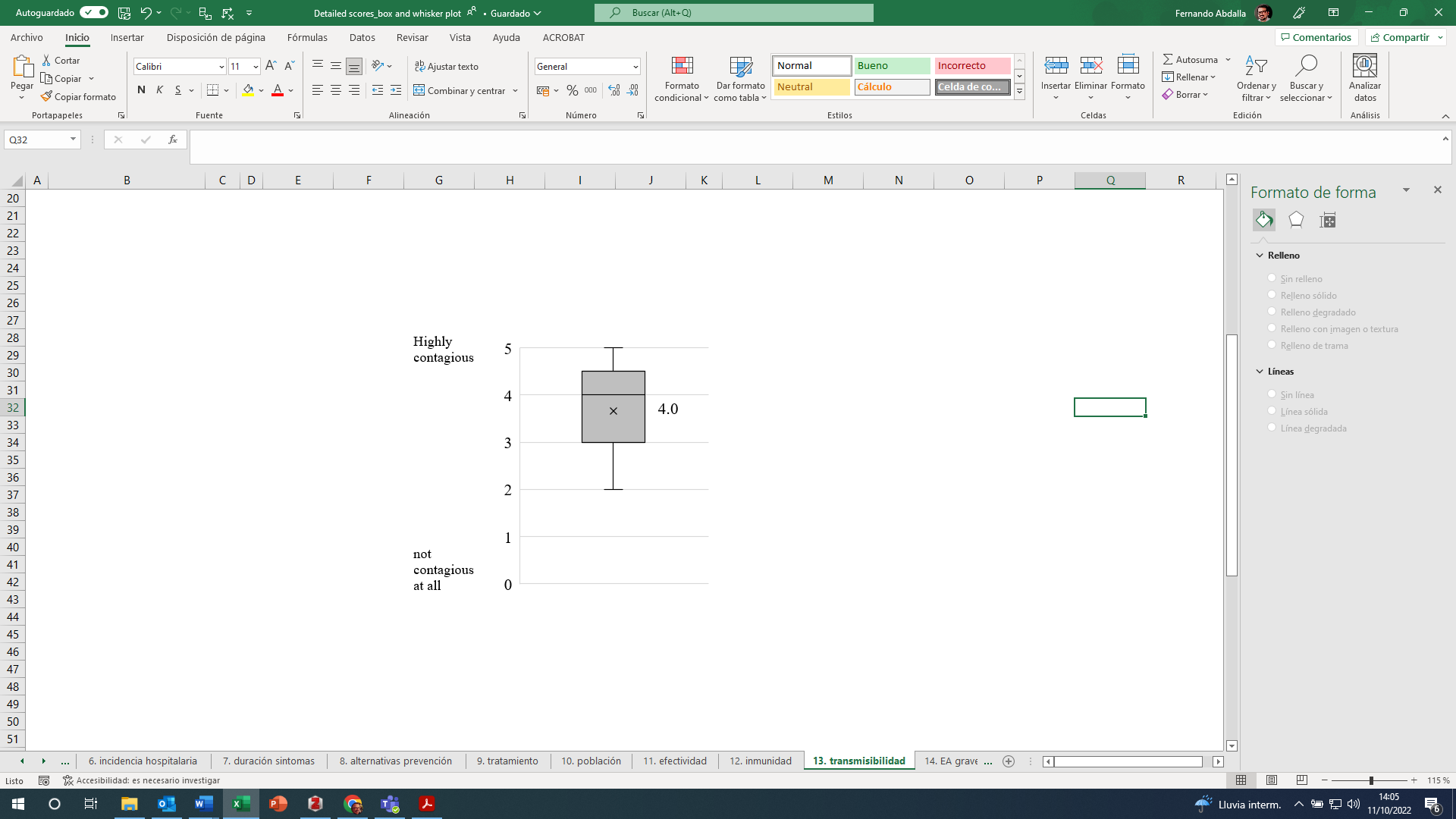 |
| --- |

The overall mean score (n=9) for the *transmissibility* criterion was 3.7 ± 1.0 (median: 4.0), reflecting that RSV infection is quite contagious. Four experts scored between 2 and 3, while five experts scored between 4 and 5.

The experts commented that this was an expected result, given the evidence presented, which indicates that infected children are much more likely to be infected than adults.

## *14. Serious adverse events*

| **Figure (S7).14. Overall score, Serious adverse events**  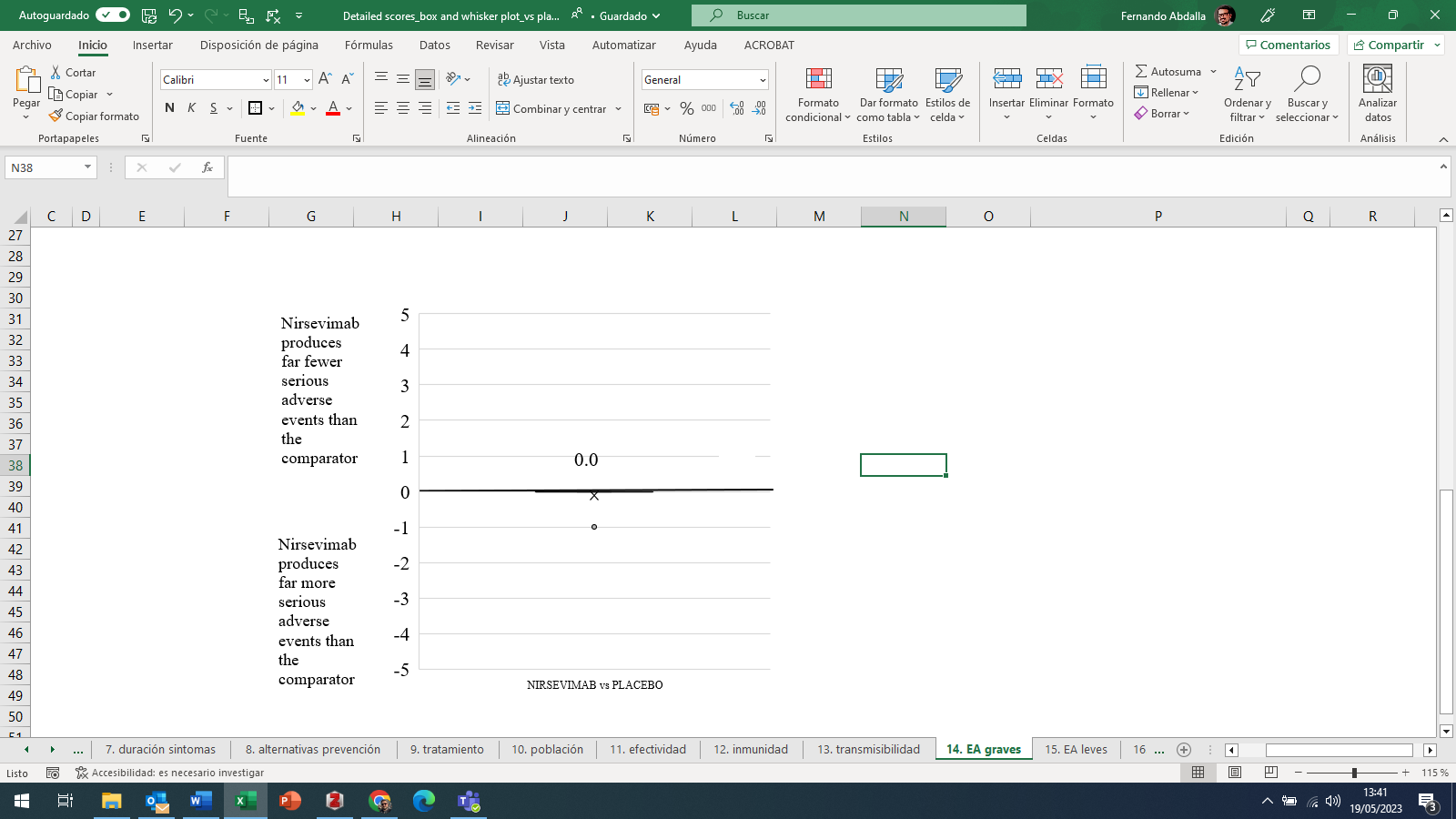 |
| --- |

The overall mean score (n=9) for the *serious adverse events* criterion was -0.1 ± 0.3, (median: 0.0), where 5.0 means that nirsevimab produces far fewer serious adverse events than the comparator, and -5.0 means that nirsevimab produces far more serious adverse events than the comparator. There was very little variability in the comparison.

The experts commented that the result was expected, given the evidence presented, which indicates that the safety profile of nirsevimab is very similar to placebo.

## *15. Mild adverse events*

| 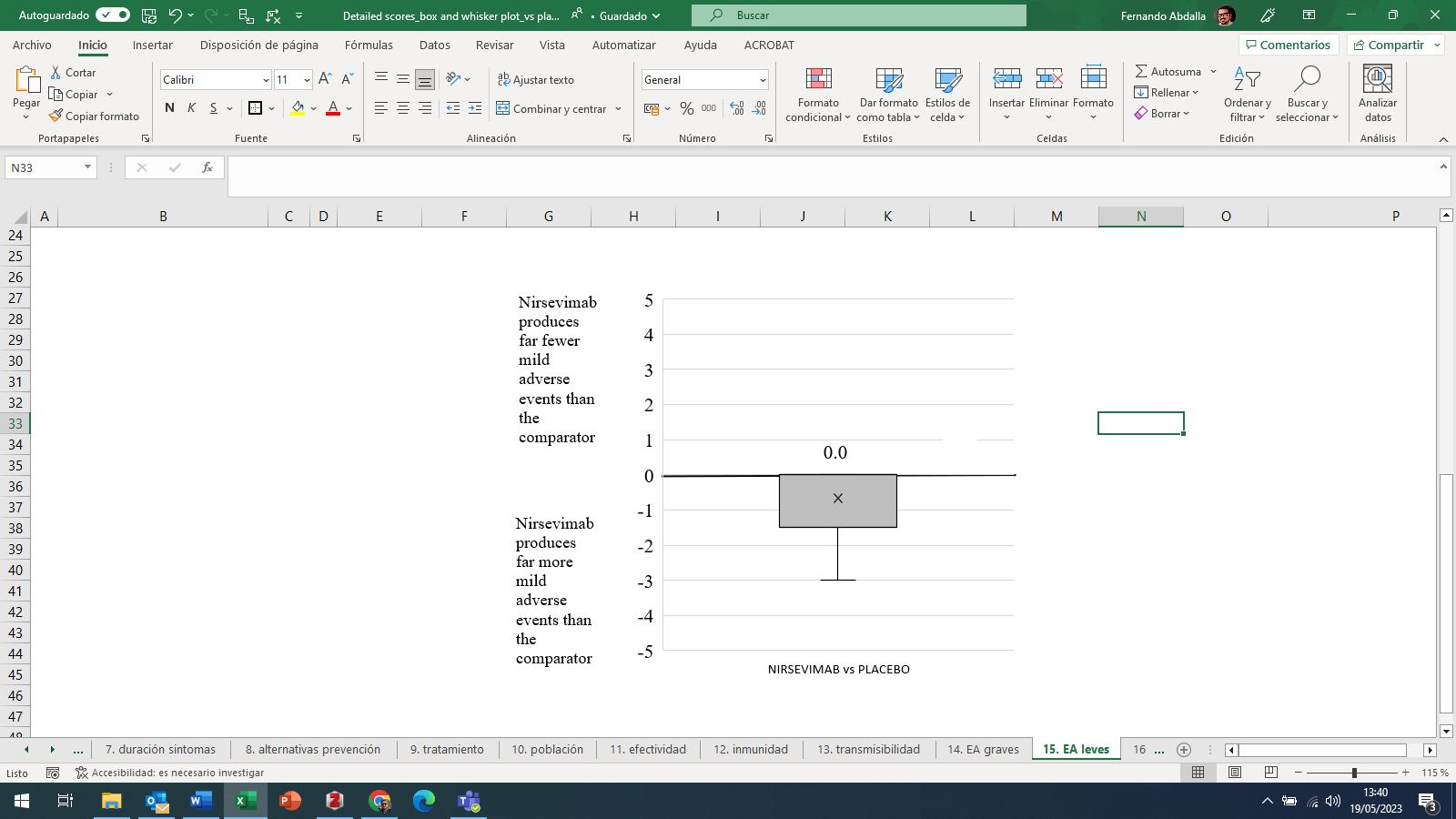**Figure (S7).15. Overall score, Mild adverse events** |
| --- |

The overall mean score (n=9) for the *mild adverse events* criterion was -0.7 ± 1.1, (median: 0.0), where 5.0 means that nirsevimab produces far fewer mild adverse events than the comparator, and -5.0 means that nirsevimab produces far more mild adverse events than the comparator. There was variability in the responses for the comparison between nirsevimab and placebo, with three experts giving negative scores, compared to the others giving null scores.

Some experts commented that biologics are often more reactogenic than placebo. Others also considered the risk of having to administer a monoclonal antibody (or any biologic) to a newborn. Another expert commented that score was based on data from the pivotal trial, where the differences are close to none. Also, some experts have commented that, given that the preventive measure is administered to such young children, the traditional "nocebo effect" would not exist in this case.

## *16. Certainty about the efficacy of the preventive measure*

| **Figure (S7).16. Overall score, Certainty about the efficacy of the preventive measure**  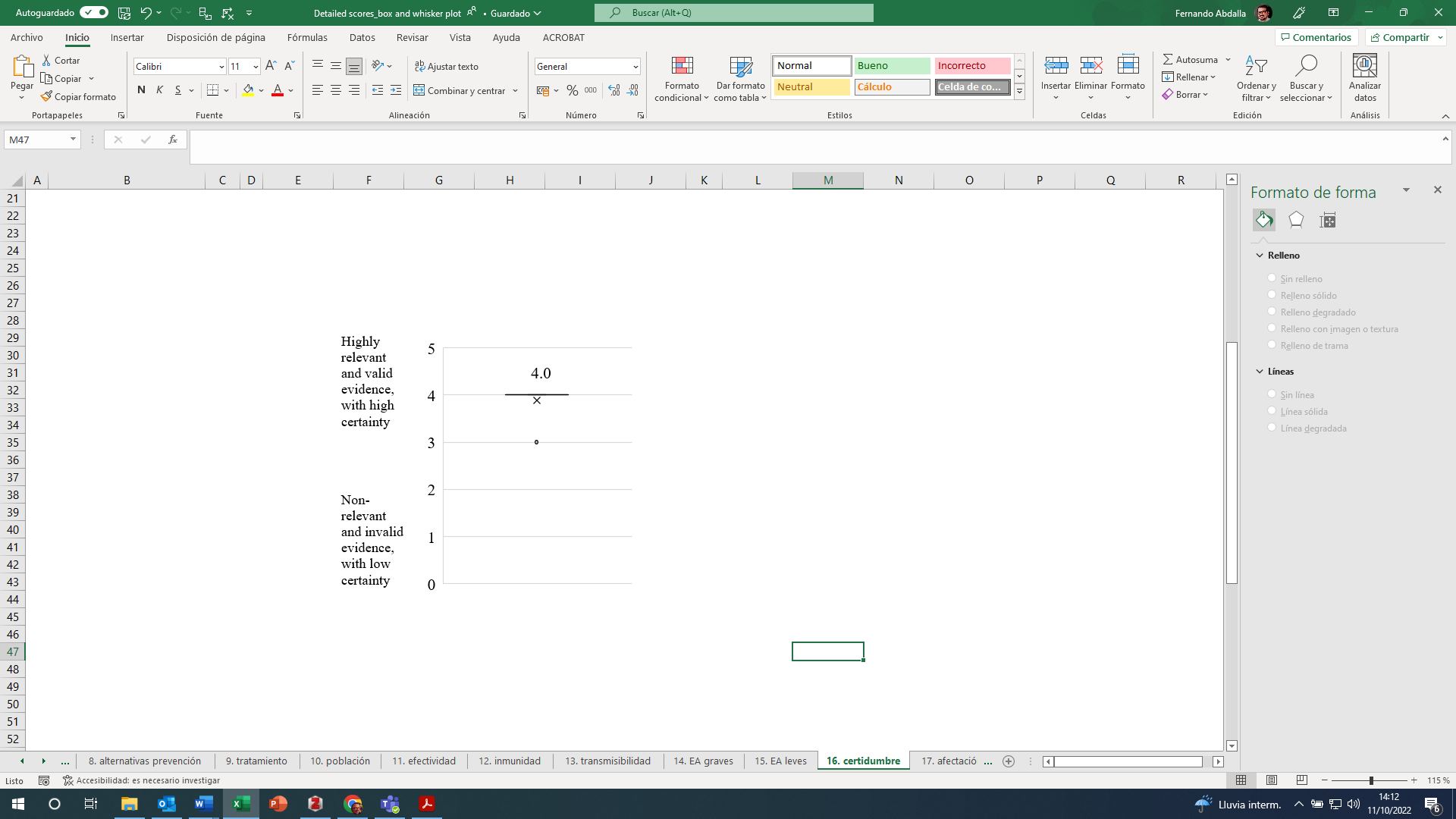 |
| --- |

The overall mean score (n=9) for the criterion *certainty about the efficacy of the preventive measure* was 3.9 ± 0.3 (median: 4.0), which reflects that the evidence is very relevant and that there is a lot of certainty about the efficacy of nirsevimab. All experts scored 4, except one expert who scored 3 (extreme case).

Experts commented that this was an expected outcome.

## *17. Impact on the population of children*

| **Figure (S7).17. Overall score, Impact on the population of children**  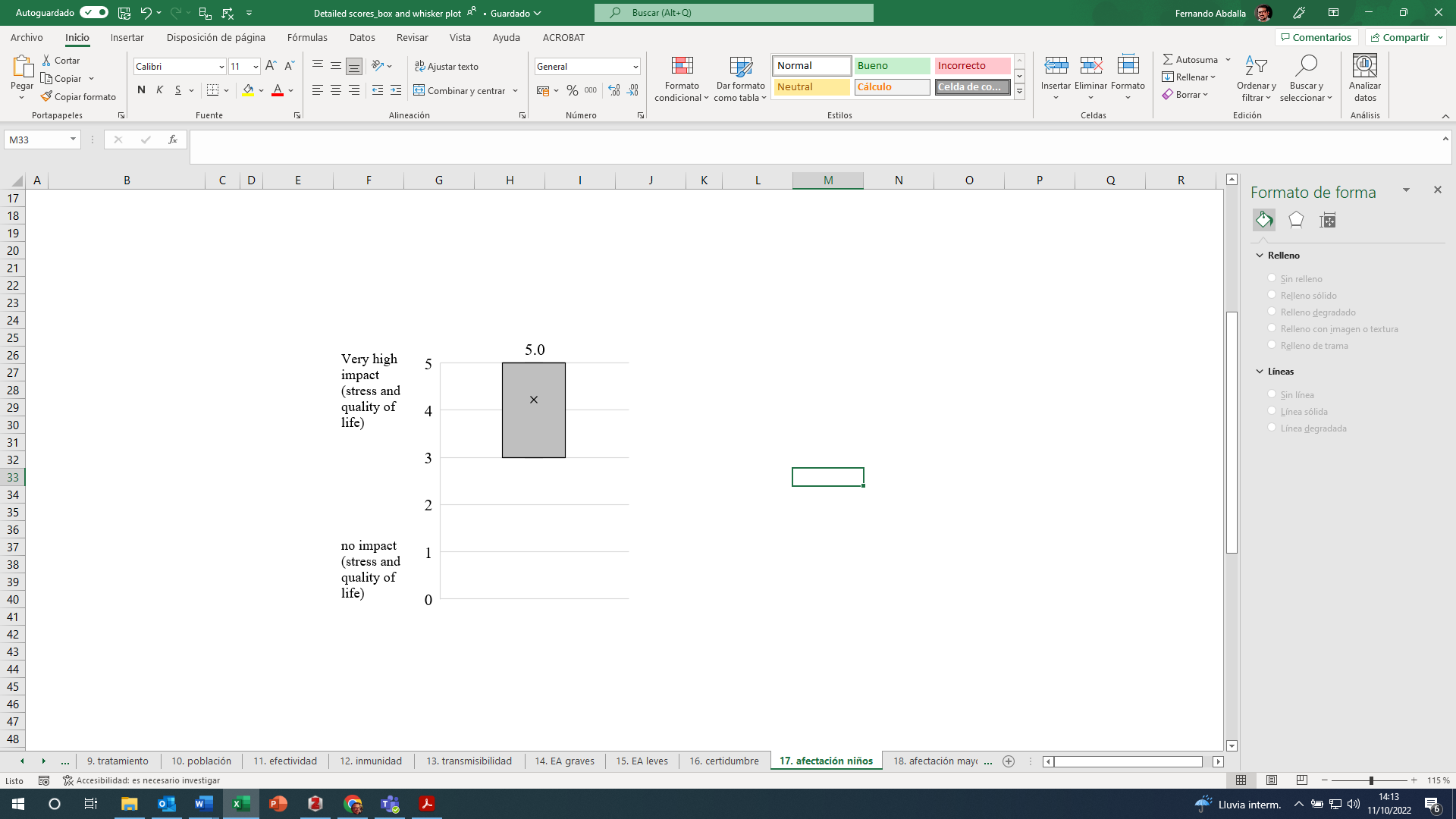 |
| --- |

The overall mean score (n=9) for the criterion *impact on the population of children* was 4.2 ± 1.0 (median: 5.0), reflecting that RSV has a very high impact on the stress levels and quality of life of infected children. Five experts scored 5, one expert scored 4, and three experts scored 3.

The experts commented that this was an expected outcome, given the evidence presented, which indicates quality of life losses of 39% in children after diagnosis, and stress levels of 79% after hospital discharge.

## *18. Impact on the population over 65 years of age*

| **Figure (S7).18. Overall score, Impact on the population over 65 years of age**  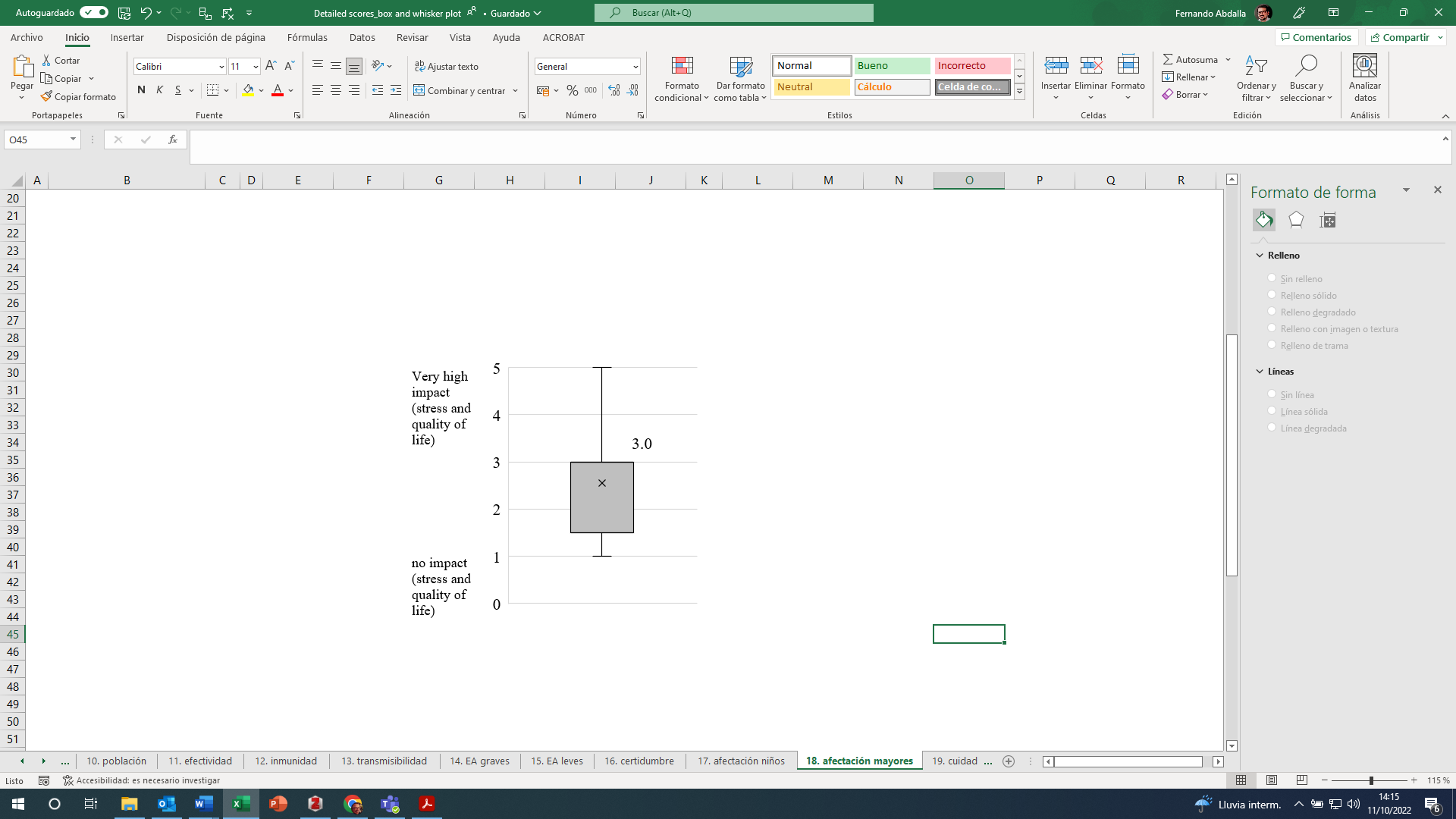 |
| --- |

The overall average score (n=9) for the criterion *impact on the population over 65 years of age* was 2.6 ± 1.2 (median: 3.0), reflecting that RSV has a low impact on stress levels and quality of life on the population over 65 years of age. The vast majority of experts scored between 1 and 3, and only one expert scored 5.

The experts commented that this was an expected outcome, given the evidence presented, which indicates quality of life losses of 11% on the population over 65 years age infected by RSV within a week of symptom onset.

## *19. Impact on caregivers*

| **Figure (S7).19. Overall score, Impact on caregivers**  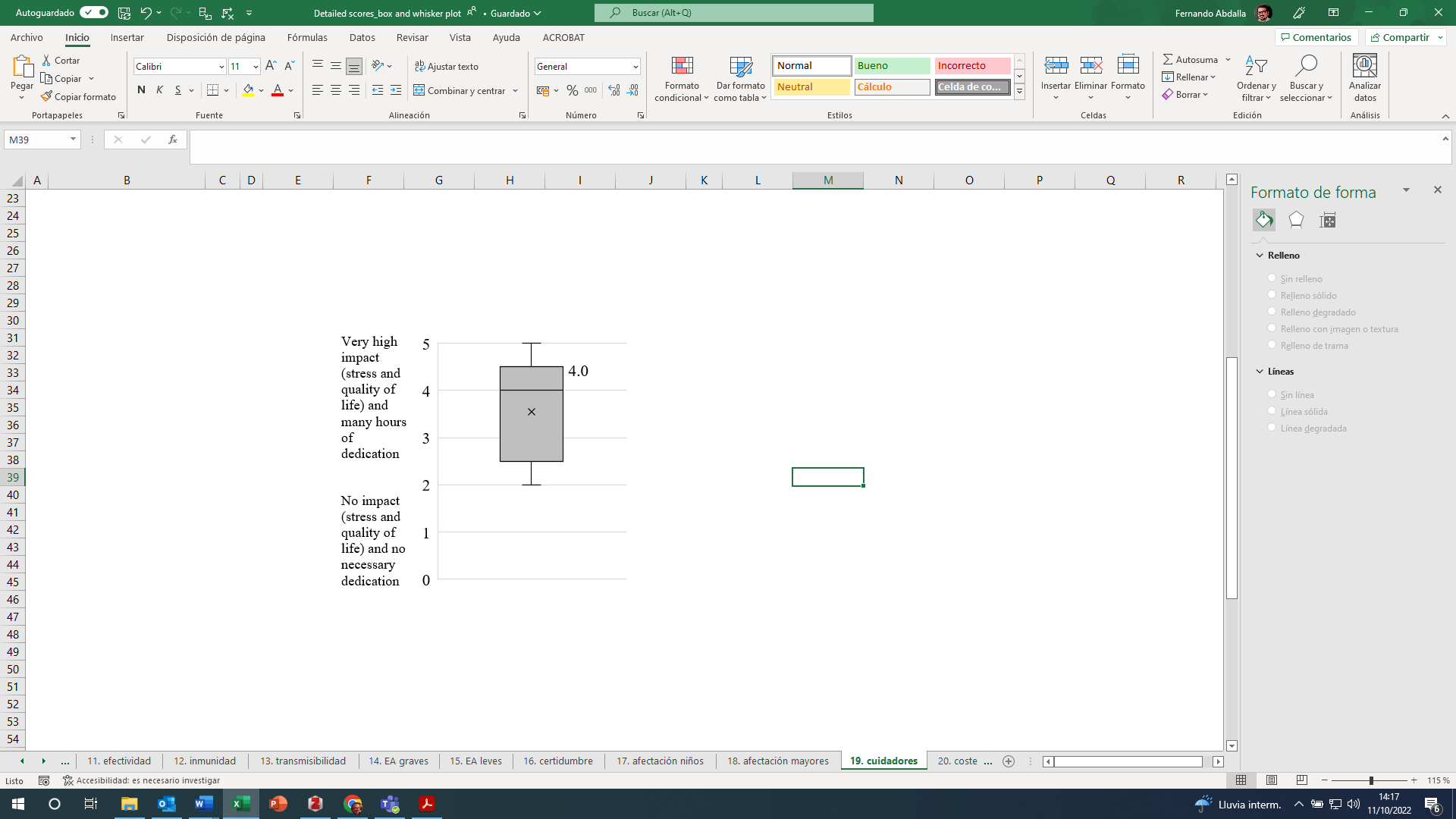 |
| --- |

The overall mean score (n=9) for the *impact on caregivers* criterion was 3.6 ± 1.1 (median: 4.0), reflecting that RSV has a high impact on the stress levels and quality of life of caregivers, who need to spend considerable hours caring for infected children. Two experts scored 2 and the remaining expert scored between 3 and 5.

The experts commented that this was an expected outcome, given the evidence presented.

## *20. Monetary cost of the preventive measure*

| **Figure (S7).20. Overall score, Monetary cost of the preventive measure**  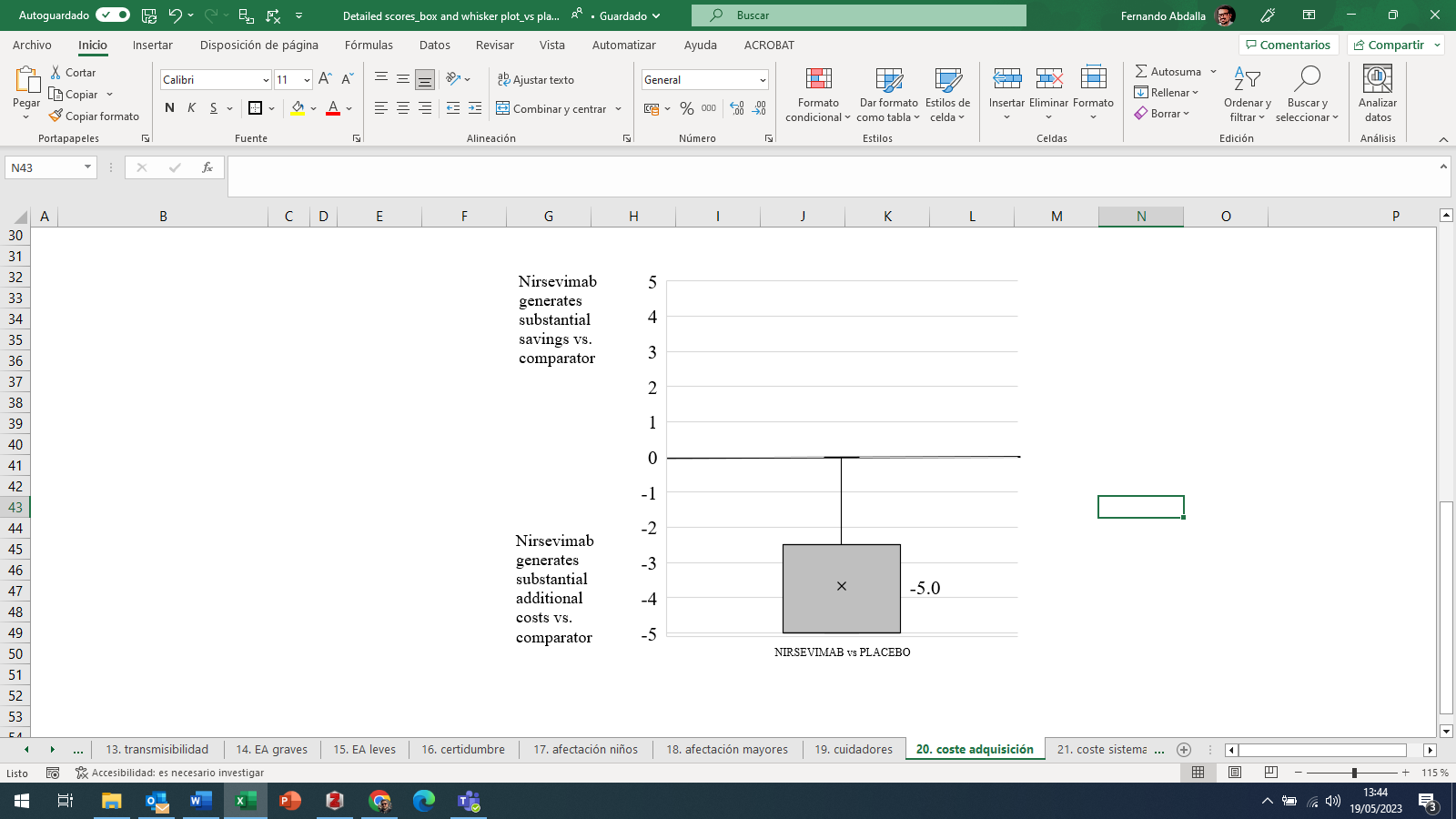 |
| --- |

The overall mean score (n=9) for the *monetary cost of the preventive measure* criterion was -3.7 ± 1.8; (median: -5.0), where 5.0 means that nirsevimab generates substantial acquisition cost savings versus the comparator, and -5.0, that nirsevimab generates substantial additional expenditure on acquisition costs versus the comparator. This was one of the criteria with the greatest variability in responses, with a standard deviation of 1.8.

Some experts mentioned the difficulty of scoring this criterion, due to the absence of an explicit cost for nirsevimab^[[1]](#footnote-2)^ . In this regard, one expert on the committee commented that he/she used an acquisition cost of €150 for nirsevimab (because of similarity to other recently introduced innovative vaccines) for this exercise.

## *21. Cost of the disease on the health system (excludes acquisition cost)*

| **Figure (S7).21. Overall score, Cost of the disease on the health system (excludes acquisition cost)**  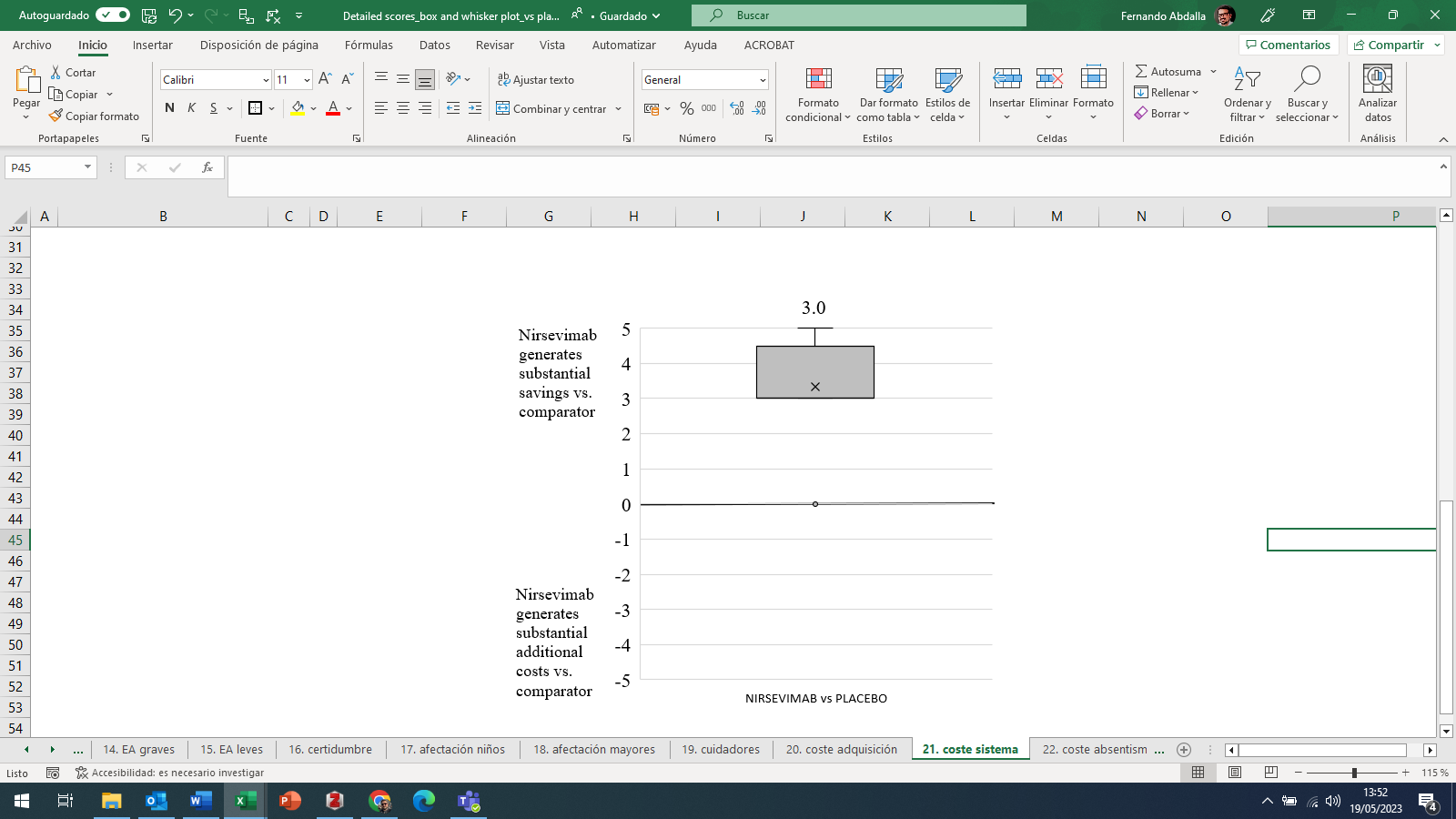 |
| --- |

The overall average score (n=9) for the *cost of the disease on the health system (excludes acquisition cost)* criterion was 3.3 ± 1.5; (median: 3.0), where 5.0 means that nirsevimab generates substantial savings vs. the comparator, and -5.0, that nirsevimab generates substantial additional costs vs. the comparator. As with the previous criterion, there was a lot of variability in the responses (standard deviation of 1.5). There were no negative scores.

The scores were awarded, in general, considering that the application of nirsevimab would generate a delay in infections, which would lead to a lesser severity of the disease and a lower use of resources. This would be due to the mechanism of action of nirsevimab, which allows children to come into contact with the virus but does not allow the attachment of the virus to the epithelium. The experts added that, due to Covid-19, many RSV infections have been delayed, indicating that a preventive measure would produce benefits in this regard (less severity and less use of resources).

## *22. Productivity cost: absenteeism*

| **Figure (S7).22. Overall score, Productivity cost: absenteeism**  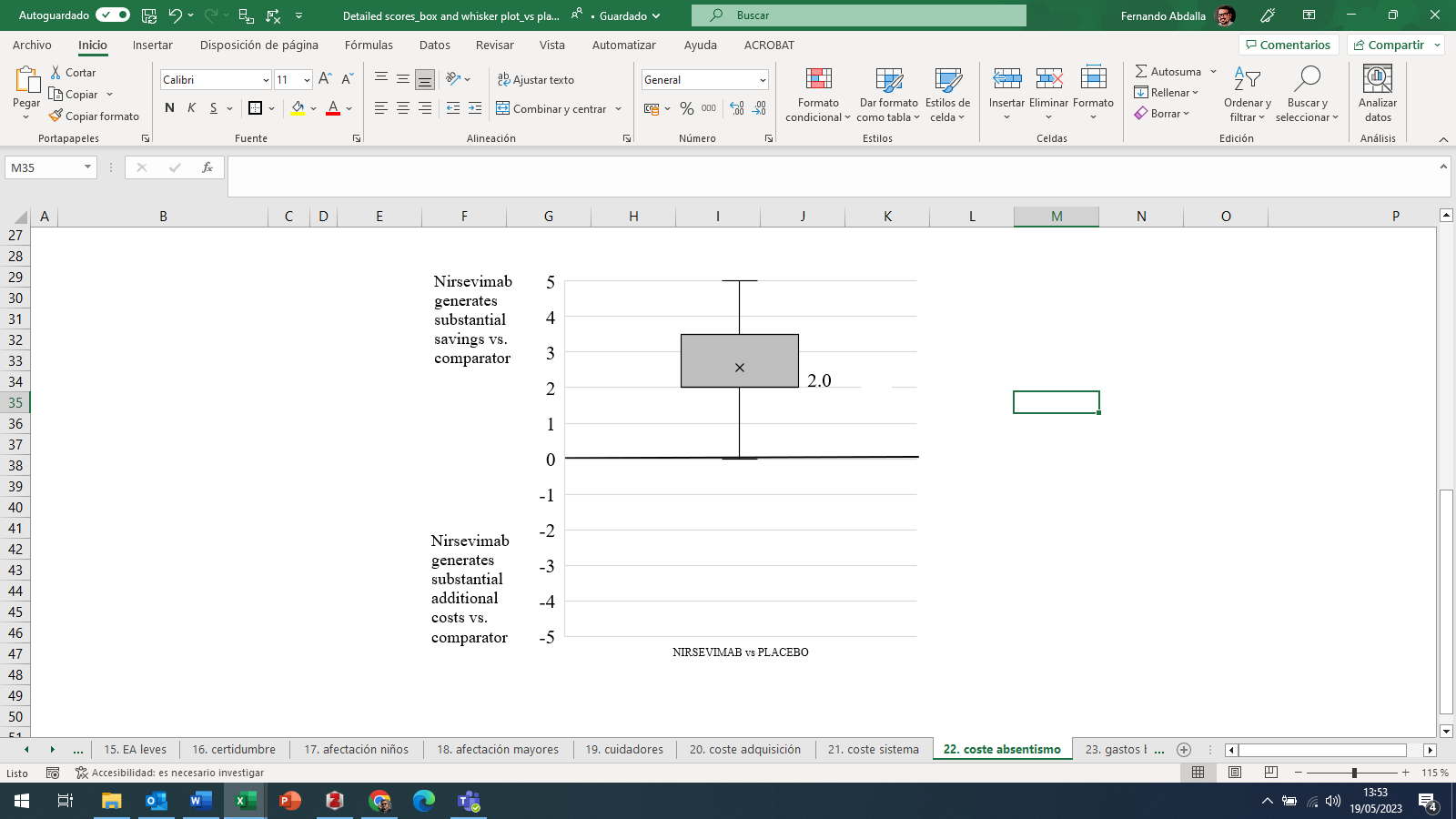 |
| --- |

The overall mean score (n=9) for the *productivity cost: absenteeism* criterion was 2.6 ± 1.4; (median: 2.0), where 5.0 means that nirsevimab generates substantial savings vs comparator, and -5.0 means that nirsevimab generates substantial additional costs vs comparator. The standard deviation was 1.4, and there were no negative scores.

The experts interpreted the result as being within the expected range and made no further comment.

## *23. Cost of the disease on the patient (out-of-pocket expenses)*

| **Figure (S7).23. Overall score, Cost of the disease on the patient (out-of-pocket expenses)**  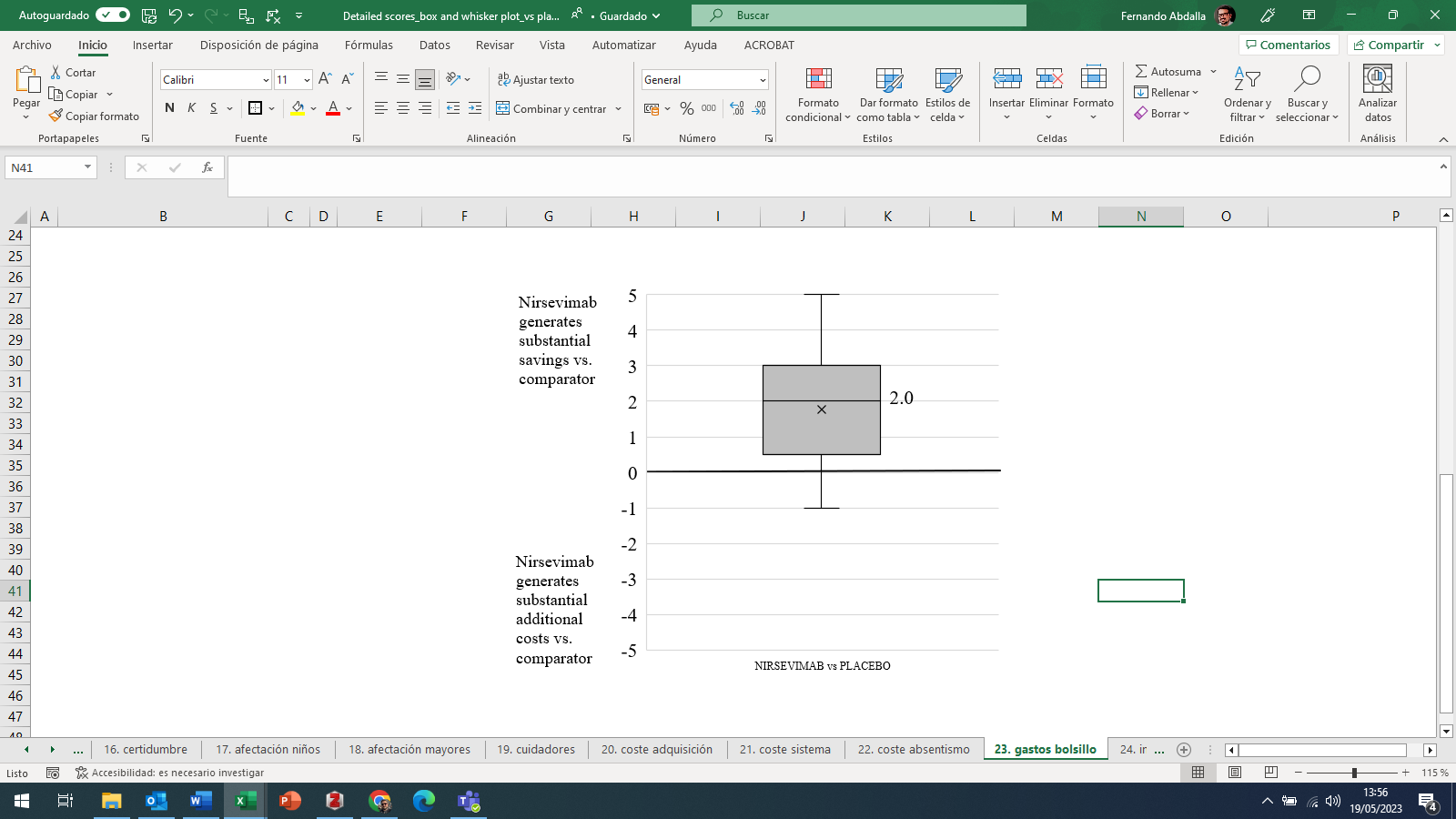 |
| --- |

The overall mean score (n=9) for the *cost of the disease on the patient (out-of-pocket expenses) criterion* was 1.8 ± 1.8; (median: 2.0), where 5.0 means that nirsevimab generates substantial savings versus the comparator, and -5.0 means that nirsevimab generates substantial additional costs versus the comparator. The standard deviation was 1.8. Only one negative score was given.

As in the previous criterion, the experts interpreted the result as being within the expected range and made no additional comments.

## *24. Impact on health inequity*

| **Figure (S7).24. Overall score, Impact on health inequity**  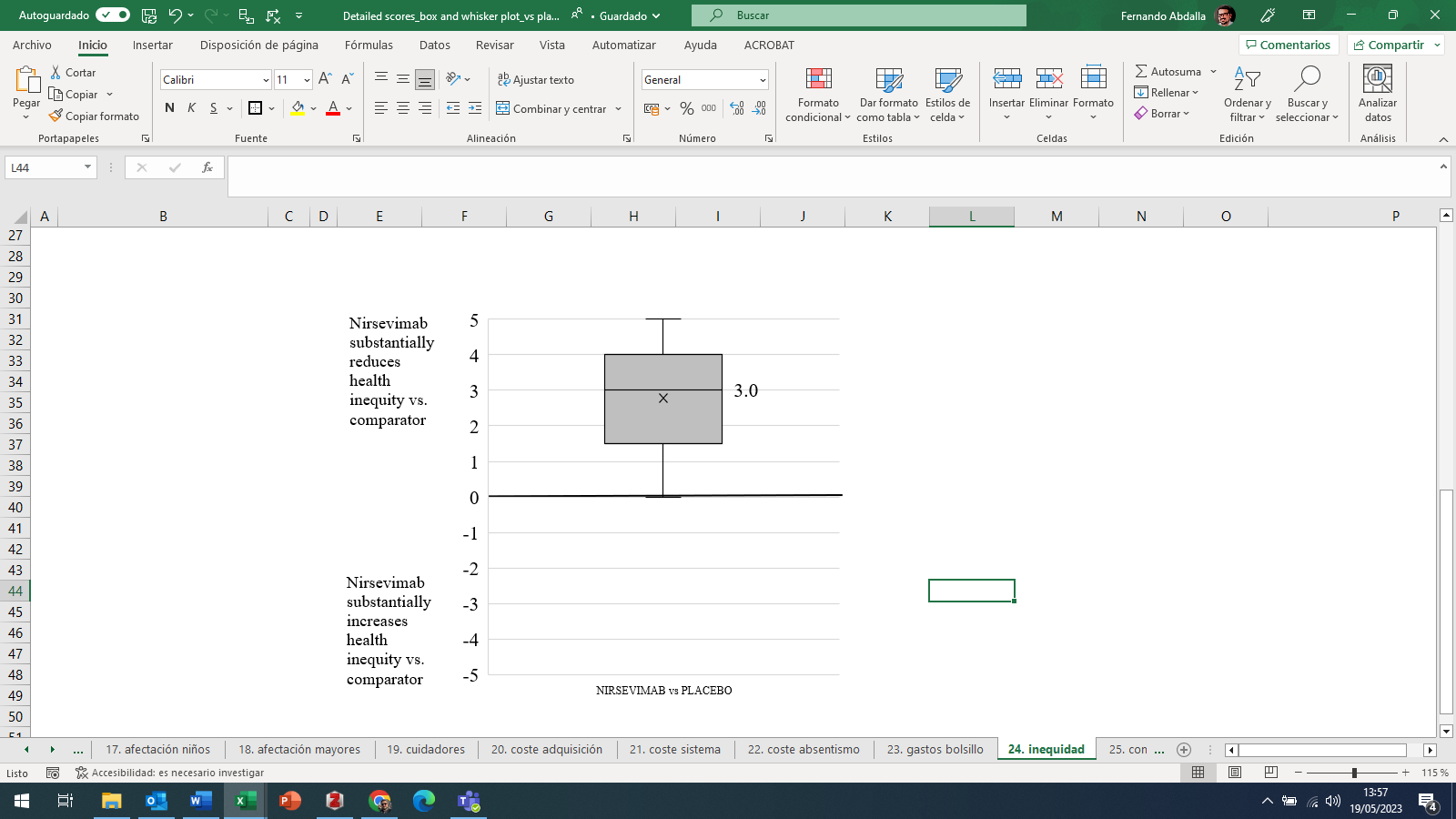 |
| --- |

The overall mean score (n=9) for the *impact on health inequity* criterion was 2.8 ± 1.6; (median: 3.0), where 5.0 means that nirsevimab substantially reduces health inequity versus comparator, and -5.0, that nirsevimab substantially increases health inequity versus comparator. The standard deviation was 1.6 and there were no negative scores.

The impression of experts who have worked in areas where there are more disadvantaged populations is that RSV has a greater burden in terms of comorbidity (and even mortality) in this population. In this sense, a preventive measure such as this could reduce inequity. They added, paraphrasing Horwitz's circles, that "by avoiding disease, poverty is avoided, and by avoiding poverty, disease is avoided", arguing that in this sense the preventive measure would reduce health inequities.

Other experts, who scored closer to zero, thought of avoidable inequities, arguing that treatment will be applied to all patients wherever they are, and without discrimination of any kind.

## *25. Public health awareness (including antibiotic resistance)*

| **Figure (S7).25. Overall score, Public health awareness (including antibiotic resistance)**  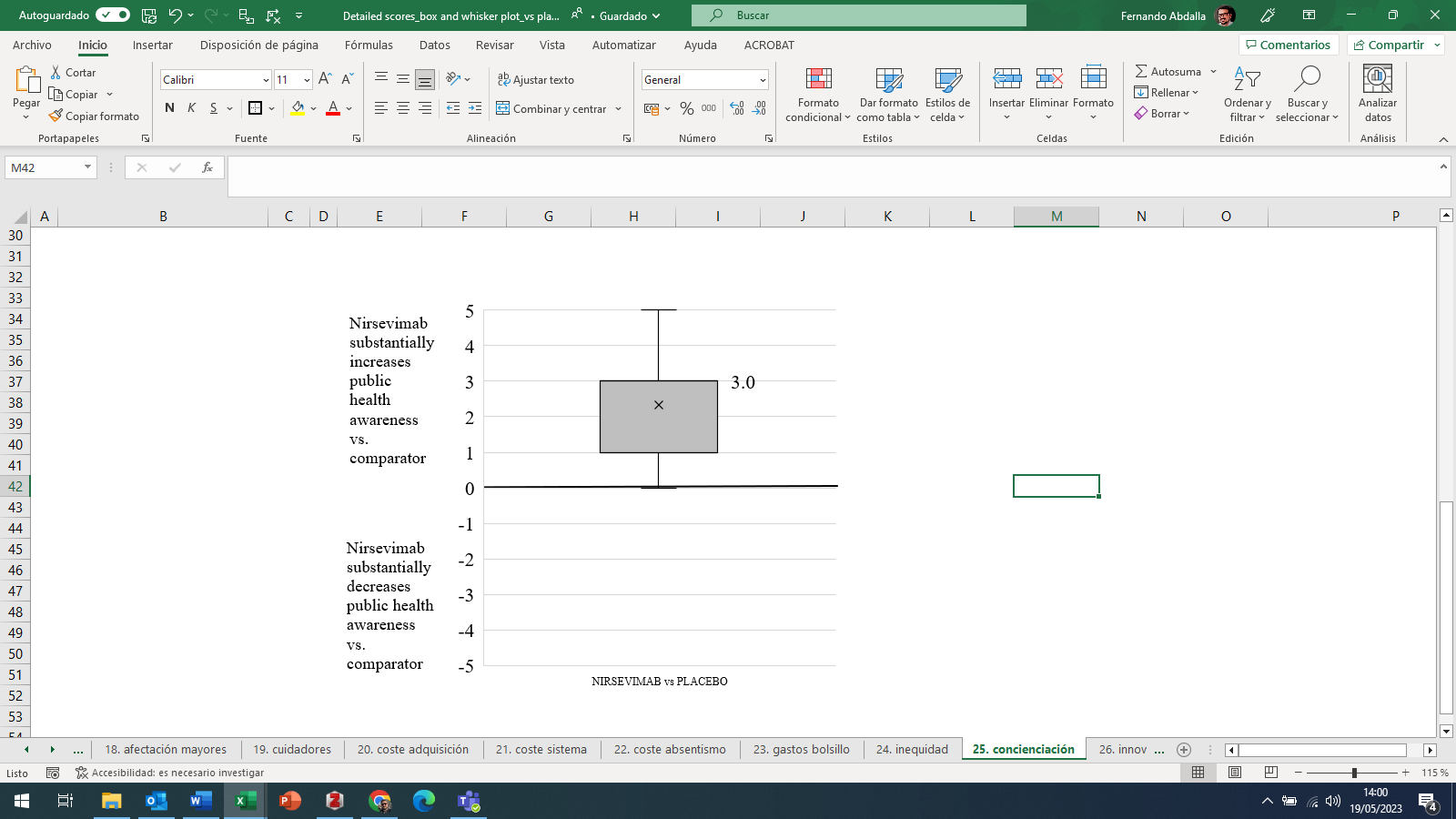 |
| --- |

The overall mean score (n=9) for the *public health awareness (includes antibiotic resistance)* criterion was 2.3 ± 1.6; (median: 3.0), where 5.0 means that nirsevimab substantially increases public health awareness versus comparator, and -5.0, that nirsevimab substantially decreases public health awareness versus comparator. The standard deviation was 1.6 and there were no negative scores.

Some experts commented that preventive measures contribute to an awareness of the importance of public health (this has become evident with Covid-19). Furthermore, they added that when a preventive measure is implemented in a vaccination schedule, for example, the awareness of mothers and fathers of children about this disease increases, and additionally, the discussion and flow of information among them increases. They also interpreted that the implementation of a new preventive measure in the schedule always generates a public debate about the disease, its severity, and the benefits of the new preventive measure.

Furthermore, one of the experts mentioned that, in one of the RSV trials (which had to be stopped due to safety issues), they recruited pregnant women who had had previous experience of children with bronchiolitis (and therefore had to use vaccines and/or preventive measures). These women offered no resistance to vaccination, which made the trial much easier. This demonstrates that an approved preventive measure in use increases society's awareness of the disease. Finally, many experts agree that a preventive measure on a prevalent childhood infection will reduce the inappropriate use of antibiotics.

## *26. Innovation stimulus*

| **Figure (S7).26. Overall score, Innovation stimulus**  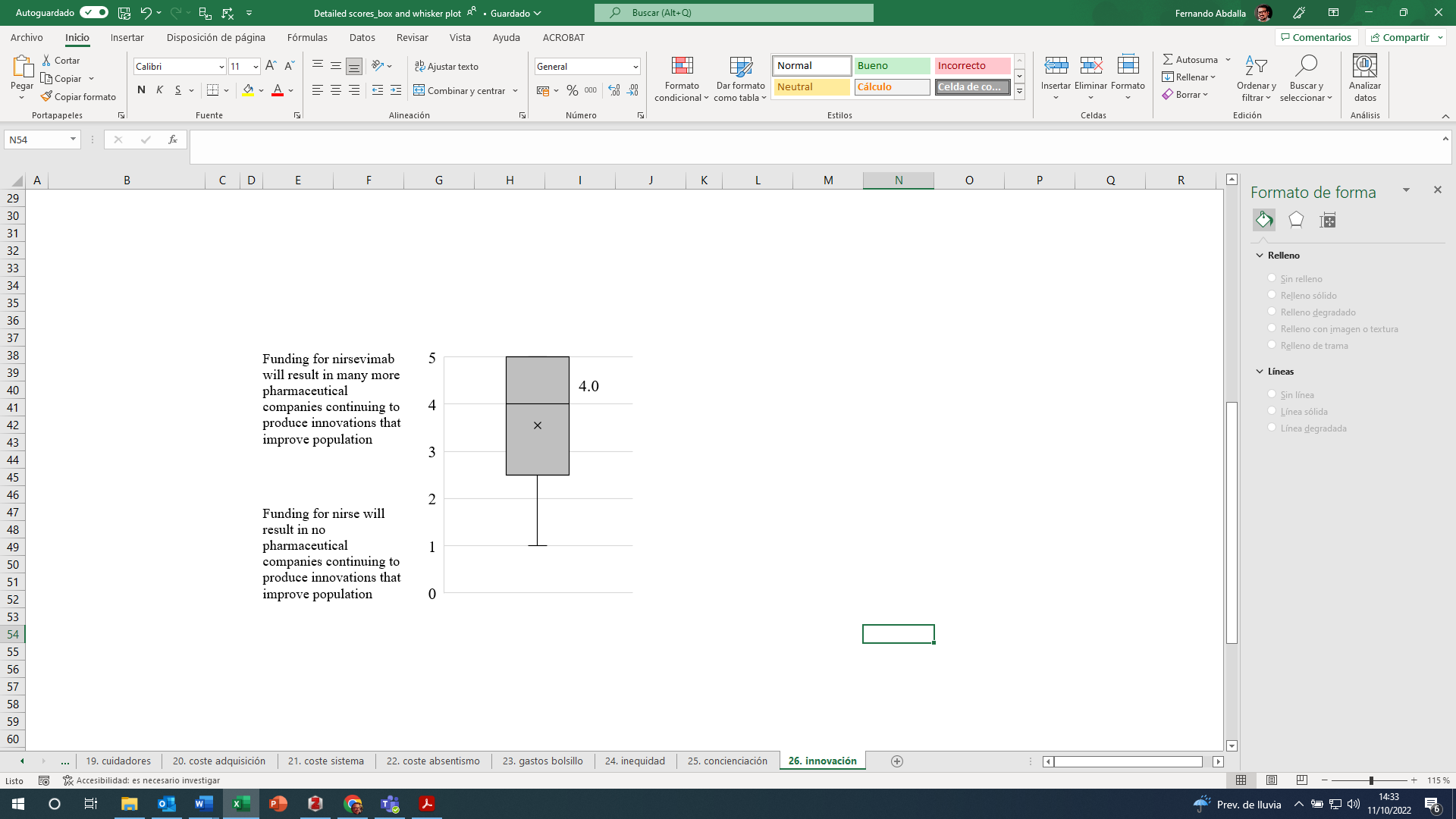 |
| --- |

The overall average score (n=9) for the *innovation stimulus* criterion was 3.6 ± 1.4 (median: 4.0), reflecting that the funding of nirsevimab will lead more laboratories to continue producing innovations that improve population health. Two experts scored 1 and 2 and the remaining experts scored between 3 and 5.

The experts interpreted this as an expected outcome and made no further comment.

1. in this MCDA, the reference of the innovative vaccines was used [↑](#footnote-ref-2)
