# Supplementary material for: Evaluation of antibody-based preventive alternatives for respiratory syncytial virus: a novel multi-criteria decision analysis framework and assessment of nirsevimab in Spain
Source: BMC Infect Dis. 2024 Jan 18;24:99. doi: 10.1186/s12879-024-08988-9 (PMC10797756; doi:10.1186/s12879-024-08988-9)
Supplement: Supplementary file 7 — Supplementary Material 7: Retest and sensitivity analysis [file 12879_2024_8988_MOESM7_ESM.docx]

**Evaluation of Antibody-based Preventive Alternatives for Respiratory Syncytial Virus: A Novel Multi-Criteria Decision Analysis Framework and Assessment of Nirsevimab in Spain**

**Authors**: Jorge Mestre-Ferrándiz^1^, Agustín Rivero^2^, Alejandro Orrico-Sánchez^3,4,5^, Álvaro Hidalgo^6,7^, Fernando Abdalla^8^, Isabel Martín^9^, Javier Álvarez^10^, Manuel García-Cenoz^11^, Maria del Carmen Pacheco^12^, María Garcés-Sánchez^13^, Néboa Zozaya^8,14^, Raúl Ortiz-de-Lejarazu^15^

**Affiliations**: ^1^Department of Economics, University Carlos III, Madrid, Spain; ^2^Department of Management, Bioregión de Salud y Bienestar (BioMad), Madrid, Spain; ^3^Department of Vaccines Research, Fundación Para el Fomento de la Investigación Sanitaria y Biomédica de la Comunitat Valenciana (Fisabio), Valencia, Spain; ^4^Catholic University of Valencia, Spain; ^5^Centro de Investigación Biomédica en Red de Epidemiología y Salud Pública (CIBERESP); ^6^Weber Foundation, Madrid, Spain; ^7^Department of Economic Analysis and Finances, University of Castilla-La Mancha. Toledo, Spain; ^8^Department of Health Affairs and Policy Research, Vivactis Weber, Madrid, Spain; ^9^Department of Primary Care, Rochapea Healthcare Center, Navarra, Spain; ^10^Department of Pediatrics, Hospital Costa del Sol, Málaga, Spain; ^11^Public Health Institute of Navarra, Navarra, Spain; ^12^Department of Epidemiology, General Directorate of Public Health, Castilla y León, Spain; ^13^Department of Pediatrics, Nazaret Healthcare Center, Valencia, Spain; ^14^Department of Quantitative Methods in Economics and Management, University Las Palmas de Gran Canaria. Las Palmas, Spain; ^15^National Influenza Centre, Scientific Advisor and Emeritus Director, School of Medicine, University of Valladolid, Castilla y León, Spain.

**SUPPLEMENTARY FILE 7: RETEST AND SENSITIVITY ANALYSIS**

# **1. Retest**

The consistency of the weights between test and retest was high, with an average intra-rater correlation coefficient (ICC) of 0.8366. Likewise, the retest scores and value estimate were very similar to the test, with average ICCs of 0.9189 and 0.9196, respectively. The retest value estimate was 4.2% higher than the test´s.

**Table (S8).1. Retest of weights, scores, and value estimates, intra-rater correlation coefficient (ICC), individual, average and probability**

| Criteria | Test vs. re-test  (mean, ∆%) | ICC  (3,1)  individual | ICC  (3,1)  average | Prob > F |
| --- | --- | --- | --- | --- |
| Weights, 1-5 scale | n.a. | 0.7190 | 0.8366 | 0.0000 |
| Scores, nirsevimab vs. placebo | n.a. | 0.8499 | 0.9189 | 0.0000 |
| Value estimates, nirsevimab vs. placebo | 0.5603 vs. 0.5841 (+4.2%) | 0.8512 | 0.9196 | 0.0010 |

# **2. Substitution of weights**

Second, to assess the extent to which a change in the experts' weights would affect the final estimated values of this MCDA, the experts' weights were replaced by the original weights of the Vaccinex framework. In the Vaccinex framework, two types of weightings were performed: under the first method (favorable scenario), a part of the study population had to assign the importance of that criterion (scale from 1 to 10, where 10 is the most important), when the vaccine was presented as positive. Under the second method (unfavorable scenario), the remaining part of the study population (each study participant only assigned weights under one method) assigned the importance of that criterion, when the vaccine was presented as negative.

For example, the criterion serious adverse events was presented in two ways: (i) favorable scenario: anyone vaccinated with Vaccinex will have no serious adverse events (seizures, severe allergic reaction): (ii) unfavorable scenario: anyone vaccinated with Vaccinex is at risk of some serious adverse effects (seizures, severe allergic reaction). In this case, the importance of serious adverse events turned out to be higher in the negative scenario than in the positive scenario.

When replacing the original weights of this MCDA in RSV with the weights assigned in the Vaccinex study, the impact on outcomes was close to none, with final estimated values ranging from 0.55 to 0.56 (vs. 0.56 in the MCDA in RSV).

**Table (S8).2. Mean value estimates, base case vs. substitution of weights (n, ∆%)**

|  | Favorable analysis | Unfavorable analysis |
| --- | --- | --- |
| Nirsevimab vs. placebo | 0.5603 vs. 0.5553 (-0.9%) | 0.5603 vs. 0.5525 (-1.4%) |

# **3. Exclusion of a marginal case**

Finally, an extreme case (outlier, measured by more than 1.5 times the central quartiles of the final estimated results) was excluded from the analysis, and the results were compared with the base case. By making this exclusion, the final estimated value increased 5.4%, resulting in 0.59.

**Figure (S8).1. Mean value estimates, base case vs. excluding outlier**

| **nirsevimab vs. placebo** |
| --- |
| 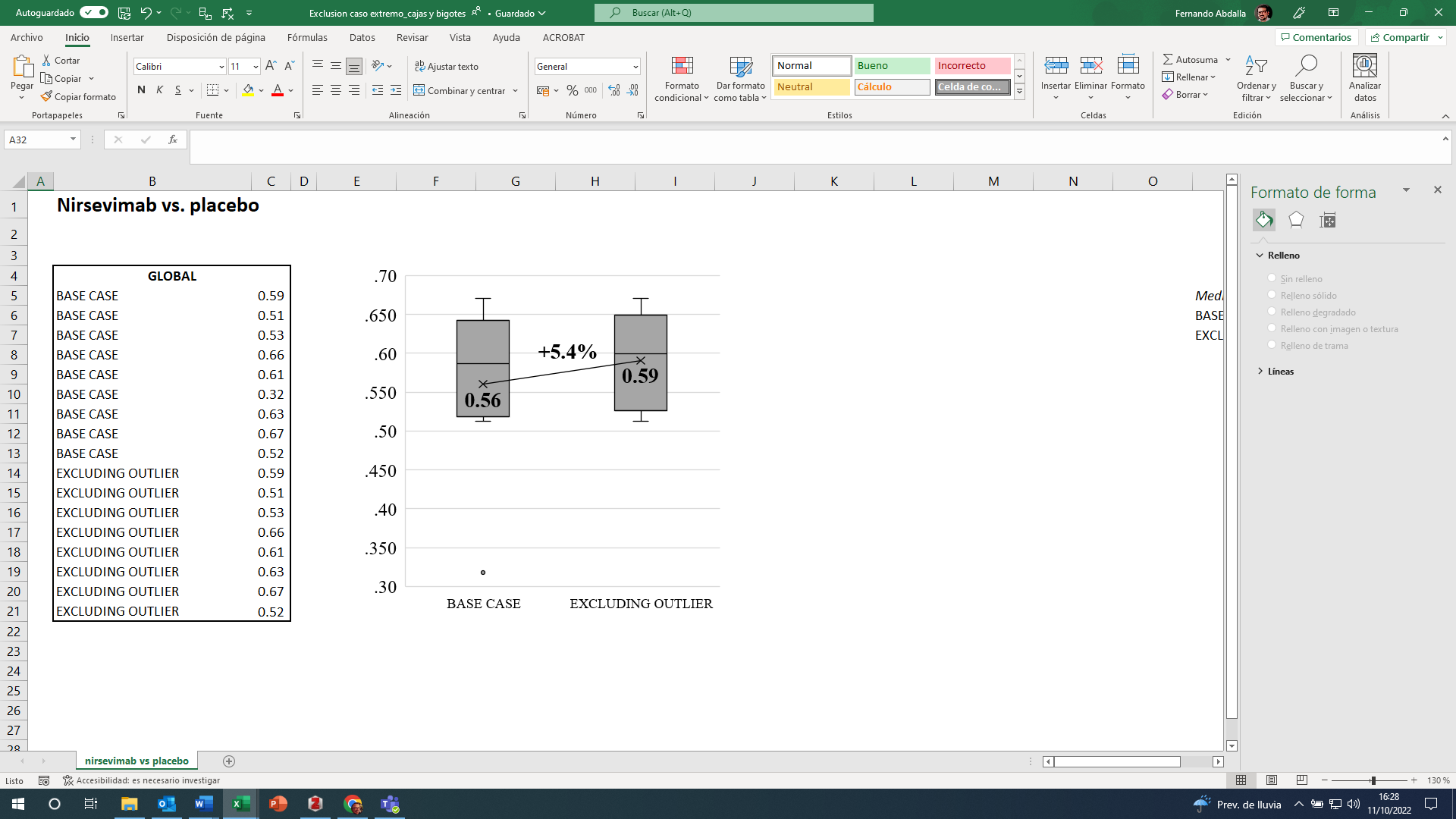 |
