# Supplementary material for: Evaluation of antibody-based preventive alternatives for respiratory syncytial virus: a novel multi-criteria decision analysis framework and assessment of nirsevimab in Spain
Source: BMC Infect Dis. 2024 Jan 18;24:99. doi: 10.1186/s12879-024-08988-9 (PMC10797756; doi:10.1186/s12879-024-08988-9)
Supplement: Supplementary file 8 — Supplementary Material 8: Nirsevimab vs. Palivizumab [file 12879_2024_8988_MOESM8_ESM.docx]

**Evaluation of Antibody-based Preventive Alternatives for Respiratory Syncytial Virus: A Novel Multi-Criteria Decision Analysis Framework and Assessment of Nirsevimab in Spain**

**Authors**: Jorge Mestre-Ferrándiz^1^, Agustín Rivero^2^, Alejandro Orrico-Sánchez^3,4,5^, Álvaro Hidalgo^6,7^, Fernando Abdalla^8^, Isabel Martín^9^, Javier Álvarez^10^, Manuel García-Cenoz^11^, Maria del Carmen Pacheco^12^, María Garcés-Sánchez^13^, Néboa Zozaya^8,14^, Raúl Ortiz-de-Lejarazu^15^

**Affiliations**: ^1^Department of Economics, University Carlos III, Madrid, Spain; ^2^Department of Management, Bioregión de Salud y Bienestar (BioMad), Madrid, Spain; ^3^Department of Vaccines Research, Fundación Para el Fomento de la Investigación Sanitaria y Biomédica de la Comunitat Valenciana (Fisabio), Valencia, Spain; ^4^Catholic University of Valencia, Spain; ^5^Centro de Investigación Biomédica en Red de Epidemiología y Salud Pública (CIBERESP); ^6^Weber Foundation, Madrid, Spain; ^7^Department of Economic Analysis and Finances, University of Castilla-La Mancha. Toledo, Spain; ^8^Department of Health Affairs and Policy Research, Vivactis Weber, Madrid, Spain; ^9^Department of Primary Care, Rochapea Healthcare Center, Navarra, Spain; ^10^Department of Pediatrics, Hospital Costa del Sol, Málaga, Spain; ^11^Public Health Institute of Navarra, Navarra, Spain; ^12^Department of Epidemiology, General Directorate of Public Health, Castilla y León, Spain; ^13^Department of Pediatrics, Nazaret Healthcare Center, Valencia, Spain; ^14^Department of Quantitative Methods in Economics and Management, University Las Palmas de Gran Canaria. Las Palmas, Spain; ^15^National Influenza Centre, Scientific Advisor and Emeritus Director, School of Medicine, University of Valladolid, Castilla y León, Spain.

**SUPPLEMENTARY FILE 8: NIRSEVIMAB VS. PALIVIZUMAB**

**Index**

[Introduction 3](#_Toc135731171)

[Evidence summary 4](#_Toc135731172)

[Description of the drug 4](#_Toc135731173)

[Criterion 10. Population in which the prevention strategy would be indicated 4](#_Toc135731174)

[Criterion 11. Efficacy of the preventive measure 5](#_Toc135731175)

[Criterion 14. Serious adverse events 7](#_Toc135731176)

[Criterion 15. Mild adverse events 7](#_Toc135731177)

[Criterion 20. Monetary cost of the preventive measure 8](#_Toc135731178)

[Criterion 21. Cost of the disease on the health system (excludes acquisition cost) 8](#_Toc135731179)

[Criterion 22. Productivity cost: absenteeism 9](#_Toc135731180)

[Criterion 23. Cost of the disease on the patient (out-of-pocket expenses) 10](#_Toc135731181)

[Criterion 24. Impact on health inequity 10](#_Toc135731182)

[Results 11](#_Toc135731183)

[A. Scores 11](#_Toc135731184)

[B. Final estimated values 12](#_Toc135731185)

[C. Sensitivity analysis 13](#_Toc135731186)

[D. Key differences between comparisons 14](#_Toc135731187)

[List of abbreviations 15](#_Toc135731188)

[References 16](#_Toc135731189)

# Introduction

The comparison between nirsevimab and palivizumab was included as part of the sensitivity analysis done in this MCDA in RSV. The main manuscript reports on the results of nirsevimab and placebo only. Usually, almost 60% of selective reporting is done because of two reasons, which are to focus on preferred findings and poor or flexible research design [1]. This is not the case of this MCDA, as the results between both comparisons were very similar, and the study design was the same. The selective reporting (inclusion of the comparison between nirsevimab and palivizumab as sensitivity analysis) of the current MCDA is justified by three key reasons, which are described below.

Firstly, and foremostly, because the comparison between nirsevimab and placebo is the one which best reflects current standard of care in Spain. Nirsevimab has been authorized by the European Medicines Agency (EMA) for the prevention of RSV lower respiratory tract disease in neonates and infants during their first RSV season, whilst palivizumab is only indicated for the prevention of serious lower respiratory tract disease requiring hospitalization caused by RSV in children at high risk for RSV disease [2,3]. The proportion of the population for whom palivizumab is indicated is 4.7%, but the actual proportion of the population to whom such a preventive measure is administered is 1.4% [4,5].

Secondly, the comparison between nirsevimab and palivizumab was more complex as it was mainly based on indirect evidence. Clinical trials of nirsevimab mostly used placebo as the comparator and focused on a much broader population than the palivizumab clinical trials [6–12]. Moreover, the direct comparison of nirsevimab vs. palivizumab was a non-inferiority trial, hence not containing efficacy results [13].

Thirdly, results were very similar between comparisons (final estimated values: nirsevimab vs. placebo: 0.56; nirsevimab vs. palivizumab: 0.58) and, notwithstanding this have a logical explanation (from both quantitative and qualitative perspectives), for reasons of clarity and simplicity, the general public healthcare professionals not familiar with the MCDA methodology will have a better understanding of the results, if only nirsevimab vs. placebo is reported as main analysis in the manuscript.

The compendium of information and results of this MCDA in RSV included information which were common to both comparisons (nirsevimab vs. placebo, and nirsevimab vs. palivizumab) as well as specific to one of the comparisons. In the current document, we only describe the specific information related to the comparison between nirsevimab vs. palivizumab.

# Evidence summary

## Description of the drug

*Palivizumab*

Palivizumab is a monoclonal antibody that binds to the fusion protein F (epitope of the A antigenic site), blocking the entry of the virus into the body's cells, especially those of the lungs[14]. It was authorized in 1999 by the EMA[15].

It is indicated for the prevention of severe lower respiratory tract illness requiring hospitalization (inpatient use) caused by RSV in children at high risk for RSV disease.[16,17]**.**

- Children born at 35 weeks gestation or less and younger than 6 months of age at the beginning of the RSV risk season.
- Children under 2 years of age who have required treatment for bronchopulmonary dysplasia within the last 6 months.
- Children under 2 years of age and with hemodynamically significant congenital heart disease.

The recommended dose of palivizumab is 15 mg/kg body weight, administered once a month during expected periods of RSV infection risk (4-5 doses required for the season).[16,17].

## Criterion 10. Population in which the prevention strategy would be indicated

**[***Criterion definition***]**: Proportion of the population in which the prevention strategy would be indicated.

**[***Type of criterion***]**: Relative

**[***Evidence available***].**

Nirsevimab is the first investigational long-acting antibody designed to provide protection against RSV for infants ≤12 months (preterm and term born) in their first RSV season, and for children with congenital heart disease or chronic lung disease in their first and second RSV season[18].

Palivizumab is indicated for premature children and/or children under 2 years of age with cardiopulmonary disease requiring hospitalization, which is estimated at 4.7% of the population. However, at present, the use of this drug in Spain is restricted and is administered to approximately 1.42% of the population, and its use may vary between Autonomous Regions.[5].

## Criterion 11. Efficacy of the preventive measure

**[***Criterion definition***]**: Level of efficacy of the RSV preventive measure.

**[***Type of criterion***]**: Relative

**[***Evidence available for nirsevimab vs palivizumab***.]**

For the scoring of nirsevimab versus palivizumab, experts should consider the immunization context of the entire population, not just the palivizumab-eligible population. In this regard, it should be noted that palivizumab is indicated only in preterm or at-risk children, and its efficacy has not been demonstrated in 100% of the population of healthy, preterm children.

The MEDLEY trial (NCT03959488[9,p.03959488]), a phase 2/3 trial, has as its primary objective the comparison of the safety of nirsevimab versus palivizumab in the palivizumab-eligible population of children^[[1]](#footnote-2)^ . For this reason, the evidence presented in this section makes an indirect comparison between the results of the phase 2b trial of nirsevimab[8] (MEDI8897, NCT02878330[7]) and the results of the pivotal trial of palivizumab (The Impact-RSV Study Group, 1998[6]). The main characteristics of each study are as follows:

**Table (S9).1. Characteristics of nirsevimab and palivizumab trials conducted in the population of palivizumab-eligible children.**

|  | Nirsevimab [8]  (phase 2b, MEDI8897, NCT02878330[7]) | Palivizumab  (The Impact-RSV Study[6]) |
| --- | --- | --- |
| Methodology | Randomized, double-blind, one administration prior to RSV season | Randomized, double-blinded, 5 administrations every 30 days |
| Population | Healthy preterm infants (29 weeks 0 days to 34 weeks 6 days gestation) | Premature infants (≤35 weeks) or infants with bronchopulmonary dysplasia |
| Comparison | Nirsevimab (50mg) *vs* placebo | Palivizumab (15 mg/kg) *vs* placebo |
| N | 1,453 (969 nirsevimab, 484 placebo) | 1,502 (1,002 palivizumab, 500 placebo) |
| Efficacy variables | Primary: medically attended RSV-associated lower respiratory tract infection up to 150 days after dose administration.  Secondary: hospitalization for RSV-associated lower respiratory tract infection up to 150 days after dose administration | Main: hospitalization with confirmed RSV infection.  Secondary: incidence of hospitalization for respiratory disease not caused by RSV and incidence of otitis media. |

Note: In this table, only the phase 2b trial for nirsevimab has been included, and the characteristics of the MELODY trial have not been included. The reason for the exclusion of the MELODY trial in the comparison between nirsevimab and palivizumab is based on the fact that the MELODY trial is not comparable to the palivizumab trial, due to the inclusion of different populations. MELODY includes infants born at a gestational age of at least 35 weeks and who were in their first RSV season at the time of the trial, whereas the palivizumab trial includes infants born preterm (≤35 weeks) or with bronchopulmonary dysplasia.

The main results are shown below:

**Nirsevimab vs placebo (healthy preterm infants).**[8]:

- The incidence of **medically attended** RSV-associated lower respiratory tract infection was 70.1% (95% CI, 52.3 to 81.2) lower with nirsevimab prophylaxis than with placebo (2.6% [25 infants] vs. 9.5% [46 infants]; p<0.001).
- The incidence of **hospitalization** for RSV-associated lower respiratory tract infection was 78.4% (95% CI, 51.9 to 90.3) lower with nirsevimab than with placebo (0.8% [8 infants] vs. 4.1% [20 infants]; p<0.001).
- Infants who received nirsevimab had a lower risk of **medically attended** RSV-associated lower respiratory tract infection than infants who received placebo (hazard ratio, 0.26; 95% CI, 0.16 to 0.43), as well as a lower risk of **hospitalization** for this condition (hazard ratio, 0.19; 95% CI, 0.08 to 0.44).
- Of the participants **hospitalized** due to RSV infection, all those who required *intensive care* (5 participants) or received *assisted ventilation* (4 participants) were in the placebo group.
- Among participants who had a **medically attended** RSV-associated lower respiratory tract infection, a smaller proportion of children in the nirsevimab group (4 [16%]) than in the placebo group (15 [32.6%]) received *supplemental oxygen*.

**Palivizumab *vs* placebo (preterm infants or infants with bronchopulmonary dysplasia).**[6]:

- Prophylaxis with palivizumab resulted in a 55% reduction in RSV **hospitalizations** (4.8% palivizumab vs. 10.6% placebo, p=0.00004).
- Infants with prematurity but without bronchopulmonary dysplasia had a 78% reduction in RSV **hospitalization** (1.8% *vs.* 8.1%, p<0.001).
- Children with bronchopulmonary dysplasia had a 39% reduction (7.9% *vs.* 12.8%, p=0.038).

In the palivizumab group, proportionally fewer total RSV hospital days (36.4 palivizumab *vs.* 62.6 placebo, per 100 children, p<0.001), fewer RSV hospital days with oxygen augmentation (30.3 vs. 50.6, p<0.001), and fewer RSV hospital days for moderate/severe lower respiratory tract disease (29.6 *vs.* 47.4, p<0.001) were observed.

## Criterion 14. Serious adverse events

*[Criterion definition***]**: Serious adverse events occurring in people receiving the preventive measure.

**[***Type of criterion***]**: Relative

**[***Available evidence nirsevimab vs palivizumab***].**

The MEDLEY[13] (NCT03959488[9,p.03959488]) is a randomized, double-blind trial of 925 children (615 nirsevimab, 310 palivizumab) comparing the safety (published results[13]) and efficacy (non-inferiority test) of nirsevimab *versus* palivizumab in the palivizumab-eligible population, separated into two cohorts: (i) preterm; (ii) with congenital heart disease (CHD) or chronic lung disease (CLD). Their results demonstrated that nirsevimab has a similar safety and tolerability profile when compared to palivizumab when administered to infants with CHD, CLD or prematurity facing their first RSV season. Serious adverse events were reported in 5.3% of preterm infants receiving nirsevimab and 6.9% of those receiving palivizumab. In children with CHD and CLD, these percentages were 20.4% and 19.2%, respectively.[13]. Reported deaths were not related to nirsevimab or palivizumab[13].

## Criterion 15. Mild adverse events

*[Criterion definition***]**: Mild adverse events occurring in people receiving the preventive measure.

**[***Type of criterion***]**: Relative

**[***Available evidence nirsevimab vs palivizumab***].**

65.0% *versus* 66.0% of preterm infants treated with palivizumab and nirsevimab, respectively, had any type of adverse event. In the CHD-CLD cohort, these percentages were 73.5% and 71.2%, respectively. Of these, only 1.9% (palivizumab) and 1.5% (nirsevimab) were considered related to the preventive measure in the preterm cohort (2.0% and 1.9% in the CHD-CLD cohort, respectively). The most common adverse events (<10% of infants in either arm of the trial) are detailed below:

**Table (S9).2. Mild adverse events, nirsevimab vs. palivizumab**

| Adverse Events | % of patients | | | | | |
| --- | --- | --- | --- | --- | --- | --- |
|  | **Preemies** | | **CHD** | | **CLD** | |
|  | **Nirsevimab** | **Palivizumab** | **Nirsevimab** | **Palivizumab** | **Nirsevimab** | **Palivizumab** |
| Cold | 6,3 | 11,8 | 17,1 | 9,1 | 6,8 | 8,8 |
| Diarrhea | 1,6 | 2,9 | 7,1 | 2,1 | 1,9 | 1,9 |
| Pyrexia | 12,5 | 8,8 | 18,6 | 9,1 | 8,2 | 5,9 |
| Rhinitis | 20,3 | 16,2 | 8,6 | 3,0 | 12,9 | 8,8 |
| URTI | 14,1 | 13,2 | 18,6 | 27,3 | 12,9 | 11,8 |
| Nasopharyngitis | 7,8 | 19,1 | 5,7 | 6,1 | 9,5 | 23,5 |

Notes: Data based on the MEDLEY trial. URTI: upper respiratory tract infection. CHD: congenital heart disease. CLD: chronic lung disease.

## Criterion 20. Monetary cost of the preventive measure

*[Definition of the criterion***]**: Acquisition cost of the preventive measure, in monetary terms.

**[***Type of criterion***]**: Relative

**[***Evidence available***].**

Nirsevimab is not yet authorized in Europe and the United States, therefore, it is not marketed in Spain (or in any other country), so its acquisition price is still unknown. Nirsevimab is intended for the protection of all children under 12 months of age (healthy, premature, and with some health problem) who are going to face their first RSV season. For this MCDA, we will assume a purchase price in line with the cost of immunization of other innovative vaccines. In addition, we assume a cost for its administration of 6,53€ (1 dose[19,20]). The target population of the strategy is 341,315 children (children born in Spain in 2020[21]).

The cost of palivizumab, assuming 5 doses of 100mg [14] at the reported Sales Price ([22]), is €4,248.20 plus an administration cost of €32.65 (5 administrations[19,20]). Palivizumab is indicated for premature children and/or children under 2 years of age with cardiopulmonary diseases requiring hospitalization, which is estimated at 4.7% of the population (n=16,042), however, the drug is currently administered to only 1.42% of the population (n=4,847). Considering these population ranges (n=4,847 to 16,042) and the cost of acquisition and administration of palivizumab (4,280.85 euros), the cost of the palivizumab prevention strategy ranges from 20.7 to 68.7 million euros per year.

## Criterion 21. Cost of the disease on the health system (excludes acquisition cost)

*[Criterion definition***]**: Costs avoided by the use of the preventive measure, in relation to the use of health resources (primary care, specialized care, emergencies and hospitalizations) of people infected with RSV, excluding the cost of acquiring the measure.

**[***Type of criterion***]**: Relative

**[***Evidence available***].**

For the comparison between **nirsevimab and palivizumab**, no specific evidence is available for this criterion. Therefore, it should be scored by trying to assume to what extent the relative improvement in health produced by nirsevimab would translate into a lower consumption of health care resources compared to palivizumab, in terms of visits to primary care, specialized care, emergencies, and hospitalizations.

What is known is that the annual direct healthcare cost of all children ≤12 months requiring medical care for RSV in Spain is between 31.8 and 49.1 million € (M€) depending on the definition used to determine RSV cases^[[2]](#footnote-3)^ . This cost represents 78% and 83% of the total direct health care cost for children <5 years, respectively, and is mainly due to the higher cost of hospitalization (22.5 M€ *vs.* 33.9 M€).[23,24].

## Criterion 22. Productivity cost: absenteeism

*[Definition of the criterion***]**: Indirect costs avoided to the system due to caregiver absenteeism as a result of using the preventive measure.

**[***Type of criterion***]**: Relative

**[***Evidence available]*

No specific evidence is available for this criterion. Therefore, it must be assumed to what extent the relative improvement in health produced by nirsevimab versus palivizumab could be reflected in a lower level of work absenteeism.

What is documented is that, according to a US study, the overall work productivity loss, absenteeism and presenteeism of mothers of infants ≤12 months preterm^[[3]](#footnote-4)^ hospitalized for RSV infection was 91% (total productivity), 73% (absenteeism) and 64% (presenteeism) at hospital discharge and 31% (total productivity), 16% (absenteeism) and 23% (presenteeism) at one month of hospital discharge[25]. In Spain, it has been estimated that indirect costs account for approximately 15.9% of total costs. In other countries, these costs would range from 5.9% to 31.9% of total costs.[26].

## Criterion 23. Cost of the disease on the patient (out-of-pocket expenses)

*[Criterion definition***]**: Out-of-pocket costs avoided (need for treatment or use of health resources) through implementation of the preventive measure, to RSV-infected people or their caregivers.

**[***Type of criterion***]**: Relative

No specific evidence is available for this criterion. Therefore, it must be assumed to what extent the relative improvement in health produced by nirsevimab would translate into lower out-of-pocket costs compared with palivizumab.

A North American study estimated average out-of-pocket expenses of $643 for hospitalized preterm infants and $214.42 for hospitalized term infants. These expenses consist of transportation, parking, food, day care, and other expenses.[27].

## Criterion 24. Impact on health inequity

*[Criterion definition***]**: Extent to which the application of the preventive measure helps to reduce health inequities between people in terms of unnecessary, avoidable, and unfair differences in the level of access and health of the population.

**[***Type of criterion***]**: Relative

The World Health Organization (WHO) defines equity in health as "the absence of unfair, avoidable or remediable differences between groups of people, whether those groups are defined socially, economically, demographically, geographically or by other dimensions of inequality (e.g., sex, gender, ethnicity, disability or sexual orientation). Health is a fundamental human right. Health equity is achieved when everyone is able to reach their full potential for health and well-being".[28].

At this time, the available evidence indicates that inequities exist regarding the use of **palivizumab**: the proportion of the population for whom the preventive measure is indicated is 4.7%, but the proportion of the population to whom such a preventive measure is administered is 1.4%[4]. The use of this measure is very restricted due to its budgetary impact and because it is not considered a cost-effective alternative in certain populations where the product is indicated.[29]. However, due to the fact that **nirsevimab** has not been introduced in the market, we do not have evidence about possible inequities that may be generated later. What we do know is that it is intended to be used for population-based prevention covering the entire population for whom it is indicated.

# Results

## Scores

**Table (S9).3: Scores, overall and by subgroup, nirsevimab vs. palivizumab**

Mean ± standard deviation (median) [min to max]

| Criteria | Overall (n=9) | Clinicians (n=4) | Managers (n=5) |
| --- | --- | --- | --- |
| **Domain 1: Severity of disease** |  |  |  |
| Severity of symptoms | 3.0 ± 1.1 (3.0) [1 to 5] | 2.5 ± 1.0 (3.0) [1 to 3] | 3.4 ± 1.1 (3.0) [2 to 5] |
| Lethality risk | 2.1 ± 1.2 (2.0) [1 to 4] | 2.3 ± 1.5 (2.0) [1 to 4] | 2.0 ± 1.0 (2.0) [1 to 3] |
| Comorbidity risk | 3.6 ± 0.9 (4.0) [2 to 5] | 3.3 ± 1.0 (3.5) [2 to 4] | 3.8 ± 0.8 (4.0) [3 to 5] |
| **Domain 2: Burden of disease** |  |  |  |
| Incidence of RSV cases | 4.0 ± 0.7 (4.0) [3 to 5] | 3.5 ± 0.6 (3.5) [3 to 4] | 4.4 ± 0.5 (4.0) [4 to 5] |
| Incidence on the outpatient setting | 4.1 ± 0.8 (4.0) [3 to 5] | 3.8 ± 1.0 (3.5) [3 to 5] | 4.4 ± 0.5 (4.0) [4 to 5] |
| Incidence on the inpatient setting | 3.3 ± 1.3 (4.0) [1 to 5] | 3.5 ± 1.7 (4.0) [1 to 5] | 3.2 ± 1.1 (4.0) [2 to 4] |
| Time of duration of acute symptoms | 2.7 ± 1.0 (2.0) [2 to 5] | 3.0 ± 1.4 (2.5) [2 to 5] | 2.4 ± 0.5 (2.0) [2 to 3] |
| **Domain 3: Prevention or Treatment Alternatives** |  |  |  |
| Prevention alternatives | 4.2 ± 0.7 (4.0) [3 to 5] | 4.0 ± 0.8 (4.0) [3 to 5] | 4.4 ± 0.5 (4.0) [4 to 5] |
| Availability of treatment | 4.4 ± 0.7 (5.0) [3 to 5] | 4.3 ± 1.0 (4.5) [3 to 5] | 4.6 ± 0.5 (5.0) [4 to 5] |
| **Domain 4: Size of population** |  |  |  |
| Population in which the prevention strategy would be indicated | 4.2 ± 1.1 (5.0) [2 to 5] | 3.8 ± 1.3 (4.0) [2 to 5] | 4.6 ± 0.9 (5.0) [3 to 5] |
| **Domain 5: Efficacy** |  |  |  |
| Efficacy of the preventive measure | 2.4 ± 1.4 (3.0) [0 to 4] | 1.5 ± 1.7 (1.5) [0 to 3] | 3.2 ± 0.4 (3.0) [3 to 4] |
| **Domain 6: Population protection** |  |  |  |
| Group immunity (collective protection) | 1.1 ± 1.2 (1.0) [0 to 3] | 1.0 ± 1.2 (1.0) [0 to 2] | 1.2 ± 1.3 (1.0) [0 to 3] |
| Transmissibility | 3.7 ± 1.0 (4.0) [2 to 5] | 3.5 ± 1.3 (3.5) [2 to 5] | 3.8 ± 0.8 (4.0) [3 to 5] |
| **Domain 7: safety** |  |  |  |
| Serious adverse events | 0.3 ± 0.5 (0.0) [0 to 1] | 0.3 ± 0.5 (0.0) [0 to 1] | 0.4 ± 0.5 (0.0) [0 to 1] |
| Mild adverse events | -0.2 ± 0.8 (0.0) [-2 to 1] | 0.0 ± 0.8 (0.0) [-1 to 1] | -0.4 ± 0.9 (0.0) [-2 to 0] |
| **Domain 8: Quality of evidence** |  |  |  |
| Certainty about the efficacy of the preventive measure | 3.9 ± 0.3 (4.0) [3 to 4] | 3.8 ± 0.5 (4.0) [3 to 4] | 4.0 ± 0.0 (4.0) [4 to 4] |
| **Domain 9: Impact on quality of life** |  |  |  |
| Impact on the population of children | 4.2 ± 1.0 (5.0) [3 to 5] | 4.0 ± 1.2 (4.0) [3 to 5] | 4.4 ± 0.9 (5.0) [3 to 5] |
| Impact on the population over 65 years of age | 2.6 ± 1.2 (3.0) [1 to 5] | 2.3 ± 1.0 (2.5) [1 to 3] | 2.8 ± 1.5 (3.0) [1 to 5] |
| Impact on caregivers | 3.6 ± 1.1 (4.0) [2 to 5] | 3.5 ± 1.3 (3.5) [2 to 5] | 3.6 ± 1.1 (4.0) [2 to 5] |
| **Domain 10: Acquisition cost** |  |  |  |
| Monetary cost of the preventive measure | 2.1 ± 1.5 (3.0) [-1 to 3] | 2.3 ± 1.5 (3.0) [0 to 3] | 2.0 ± 1.7 (3.0) [-1 to 3] |
| **Domain 11: Impact on other costs** |  |  |  |
| Cost of the disease on the health system (excludes acquisition cost) | 3.0 ± 1.7 (4.0) [0 to 5] | 1.8 ± 1.7 (1.5) [0 to 4] | 4.0 ± 0.7 (4.0) [3 to 5] |
| Productivity cost: absenteeism | 2.2 ± 1.3 (2.0) [0 to 4] | 1.3 ± 1.0 (1.5) [0 to 2] | 3.0 ± 1.0 (3.0) [2 to 4] |
| Cost of the disease on the patient (out-of-pocket expenses) | 2.2 ± 1.2 (2.0) [0 to 4] | 1.8 ± 1.5 (2.0) [0 to 3] | 2.6 ± 0.9 (2.0) [2 to 4] |
| **Domain 12: Social benefits** |  |  |  |
| Impact on health inequity | 2.3 ± 1.4 (2.0) [0 to 5] | 1.8 ± 1.5 (2.0) [0 to 3] | 2.8 ± 1.3 (2.0) [2 to 5] |
| Public health awareness (including antibiotic resistance) | 1.8 ± 1.5 (2.0) [0 to 5] | 1.3 ± 1.0 (1.5) [0 to 2] | 2.2 ± 1.8 (2.0) [0 to 5] |
| Innovation stimulus | 3.6 ± 1.4 (4.0) [1 to 5] | 3.8 ± 1.9 (4.5) [1 to 5] | 3.4 ± 1.1 (3.0) [2 to 5] |

## Final estimated values

**Table (S9).4: Value estimates, overall and by subgroup, nirsevimab vs. palivizumab**

Mean ± standard deviation [min to max]

| Criteria | Overall (n=9) | Clinicians (n=4) | Managers (n=5) |
| --- | --- | --- | --- |
| **Domain 1: Severity of disease** |  |  |  |
| Severity of symptoms | 0.03 ± 0.01 [0.01 to 0.04] | 0.02 ± 0.01 [0.01 to 0.03] | 0.03 ± 0.01 [0.01 to 0.04] |
| Lethality risk | 0.02 ± 0.01 [0.00 to 0.04] | 0.02 ± 0.02 [0.00 to 0.04] | 0.02 ± 0.01 [0.01 to 0.03] |
| Comorbidity risk | 0.03 ± 0.01 [0.02 to 0.04] | 0.03 ± 0.01 [0.02 to 0.03] | 0.03 ± 0.01 [0.02 to 0.04] |
| **Domain 2: Burden of disease** |  |  |  |
| Incidence of RSV cases | 0.03 ± 0.01 [0.02 to 0.05] | 0.03 ± 0.01 [0.02 to 0.04] | 0.04 ± 0.01 [0.03 to 0.05] |
| Incidence on the outpatient setting | 0.04 ± 0.01 [0.01 to 0.05] | 0.03 ± 0.02 [0.01 to 0.05] | 0.04 ± 0.01 [0.03 to 0.05] |
| Incidence on the inpatient setting | 0.03 ± 0.01 [0.01 to 0.06] | 0.03 ± 0.02 [0.01 to 0.06] | 0.03 ± 0.01 [0.01 to 0.04] |
| Time of duration of acute symptoms | 0.02 ± 0.01 [0.01 to 0.04] | 0.02 ± 0.01 [0.01 to 0.04] | 0.02 ± 0.01 [0.01 to 0.02] |
| **Domain 3: Prevention or Treatment Alternatives** |  |  |  |
| Prevention alternatives | 0.03 ± 0.01 [0.02 to 0.05] | 0.03 ± 0.01 [0.03 to 0.04] | 0.03 ± 0.01 [0.02 to 0.05] |
| Availability of treatment | 0.04 ± 0.02 [0.01 to 0.06] | 0.04 ± 0.02 [0.01 to 0.06] | 0.04 ± 0.01 [0.02 to 0.05] |
| **Domain 4: Size of population** |  |  |  |
| Population in which the prevention strategy would be indicated | 0.03 ± 0.01 [0.02 to 0.05] | 0.02 ± 0.00 [0.02 to 0.03] | 0.04 ± 0.01 [0.03 to 0.05] |
| **Domain 5: Efficacy** |  |  |  |
| Efficacy of the preventive measure | 0.02 ± 0.01 [0.00 to 0.04] | 0.01 ± 0.02 [0.00 to 0.03] | 0.03 ± 0.01 [0.02 to 0.04] |
| **Domain 6: Population protection** |  |  |  |
| Group immunity (collective protection) | 0.01 ± 0.01 [0.00 to 0.03] | 0.01 ± 0.01 [0.00 to 0.01] | 0.01 ± 0.01 [0.00 to 0.03] |
| Transmissibility | 0.03 ± 0.01 [0.01 to 0.05] | 0.03 ± 0.01 [0.01 to 0.04] | 0.03 ± 0.01 [0.02 to 0.05] |
| **Domain 7: safety** |  |  |  |
| Serious adverse events | 0.00 ± 0.00 [0.00 to 0.01] | 0.00 ± 0.00 [0.00 to 0.00] | 0.00 ± 0.01 [0.00 to 0.01] |
| Mild adverse events | 0.00 ± 0.01 [-0.02 to 0.00] | 0.00 ± 0.00 [0.00 to 0.00] | 0.00 ± 0.01 [-0.02 to 0.00] |
| **Domain 8: Quality of evidence** |  |  |  |
| Certainty about the efficacy of the preventive measure | 0.03 ± 0.01 [0.02 to 0.05] | 0.04 ± 0.01 [0.02 to 0.05] | 0.03 ± 0.01 [0.03 to 0.04] |
| **Domain 9: Impact on quality of life** |  |  |  |
| Impact on the population of children | 0.04 ± 0.01 [0.02 to 0.05] | 0.03 ± 0.01 [0.02 to 0.04] | 0.04 ± 0.01 [0.02 to 0.05] |
| Impact on the population over 65 years of age | 0.02 ± 0.01 [0.01 to 0.04] | 0.02 ± 0.01 [0.01 to 0.02] | 0.02 ± 0.01 [0.01 to 0.04] |
| Impact on caregivers | 0.02 ± 0.01 [0.01 to 0.04] | 0.02 ± 0.01 [0.01 to 0.04] | 0.02 ± 0.01 [0.01 to 0.03] |
| **Domain 10: Acquisition cost** |  |  |  |
| Monetary cost of the preventive measure | 0.02 ± 0.01 [-0.01 to 0.03] | 0.02 ± 0.01 [0.00 to 0.03] | 0.02 ± 0.01 [-0.01 to 0.03] |
| **Domain 11: Impact on other costs** |  |  |  |
| Cost of the disease on the health system (excludes acquisition cost) | 0.03 ± 0.02 [0.00 to 0.05] | 0.02 ± 0.02 [0.00 to 0.04] | 0.03 ± 0.01 [0.02 to 0.05] |
| Productivity cost: absenteeism | 0.01 ± 0.01 [0.00 to 0.00] | 0.01 ± 0.01 [0.00 to 0.02] | 0.02 ± 0.01 [0.01 to 0.03] |
| Cost of the disease on the patient (out-of-pocket expenses) | 0.01 ± 0.01 [0.00 to 0.02] | 0.01 ± 0.01 [0.00 to 0.02] | 0.01 ± 0.00 [0.01 to 0.02] |
| **Domain 12: Social benefits** |  |  |  |
| Impact on health inequity | 0.02 ± 0.01 [0.00 to 0.03] | 0.02 ± 0.01 [0.00 to 0.03] | 0.02 ± 0.01 [0.01 to 0.03] |
| Public health awareness (including antibiotic resistance) | 0.01 ± 0.01 [0.00 to 0.02] | 0.01 ± 0.01 [0.00 to 0.02] | 0.01 ± 0.01 [0.00 to 0.02] |
| Innovation stimulus | 0.02 ± 0.01 [0.00 to 0.03] | 0.02 ± 0.01 [0.00 to 0.03] | 0.02 ± 0.01 [0.00 to 0.03] |
| **Total** | **0.58 ± 0.12 [0.29 to 0.67]** | **0.54 ± 0.18 [0.29 to 0.67]** | **0.62 ± 0.04 [0.56 to 0.66]** |

## Sensitivity analysis

**Table (S9).5. Retest of scores, and value estimates, intra-rater correlation coefficient (ICC), individual, average and probability**

| Criteria | Test vs. re-test  (mean, ∆%) | ICC  (3,1)  individual | ICC  (3,1)  average | Prob > F |
| --- | --- | --- | --- | --- |
| Scores, nirsevimab vs. palivizumab | n.a. | 0.8411 | 0.9137 | 0.0000 |
| Value estimates, nirsevimab vs. palivizumab | 0.5848 vs. 0.6053 (+3.5%) | 0.8768 | 0.9344 | 0.0000 |

**Table (S9).6. Mean value estimates, base case vs. substitution of weights (n, ∆%)**

|  | Favorable analysis | Unfavorable analysis |
| --- | --- | --- |
| Nirsevimab vs. palivizumab | 0.5848 vs. 0.5786 (-1.1%) | 0.5848 vs. 0.5797 (-0.9%) |

**Figure (S9).1. Mean value estimates, base case vs. excluding outlier**

| **nirsevimab vs. palivizumab** |
| --- |
| 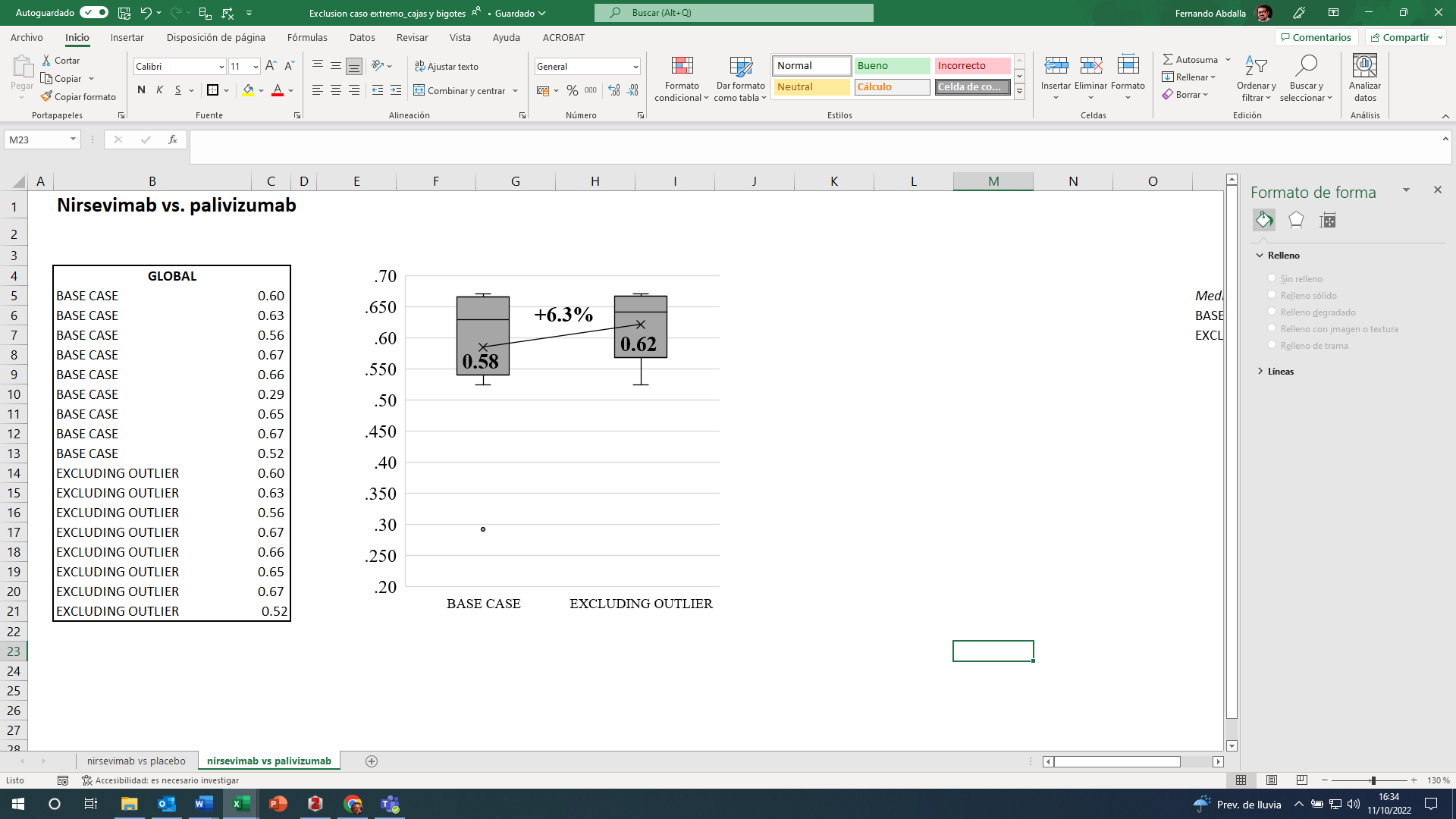 |

## Key differences between comparisons

The two criteria which mark the distinction between the comparisons between nirsevimab vs placebo and nirsevimab vs. palivizumab are: (i) in efficacy of the preventive measure, nirsevimab has an estimated value of 0.04 vs. placebo and 0.02 vs. palivizumab; (ii) in monetary cost of the preventive measure, nirsevimab obtained an estimated value of -0.03 vs. placebo, and 0.02 vs. palivizumab. (iii) on the remaining relative criteria, final estimated values were close to equal, in both comparisons.

# List of abbreviations

ALRTI acute lower respiratory tract infections

CHD congenital heart disease

CHMP Committee for the Evaluation of Medicinal Products for Human Use (EMA)

CLD chronic lung disease

ED emergency department

EMA European Medicines Agency

GCP Good Clinical Practices

ICH International Conference on Harmonization of Technical Requirements for Pharmaceuticals for Human Use

MCDA multi-criteria decision analysis

OR odd ratio

R_0_ R-naught, basic reproduction number

RSV respiratory syncytial virus

WHO World Health Organization

# References

[1] Steen JT van der, Bogert CA van den, Soest-Poortvliet MC van, et al. Determinants of selective reporting: A taxonomy based on content analysis of a random selection of the literature. PLOS ONE. 2018;13:e0188247.

[2] EMA. Beyfortus marketing authorisation [Internet]. Eur. Med. Agency. 2022. Available from: https://www.ema.europa.eu/en/medicines/human/EPAR/beyfortus.

[3] EMA. Authorisation details of palivizumab (Synagis®) [Internet]. Eur. Med. Agency. 1999 [cited 2022 Oct 25]. Available from: https://www.ema.europa.eu/en/medicines/human/EPAR/synagis.

[4] Agència d’informació, Avaluació i Qualitat en Salut. Recomanacions d’ús de palivizumab per a la prevenció de les hospitalitzacions per infecció greu per virus sincític respiratori. 2012;32.

[5] Drago G, Roiz J, Kazmierska P, et al. Modeled impact of Nirsevimab against respiratory syncytial virus (RSV) AMONG Spanish infants experiencing their first RSV season [oral presentation O024 / #1329]. ESPID Athens 9-13th May [Internet]. 2022; Available from: https://keneswp.azureedge.net/wp-content/uploads/sites/162/2022/05/ESPID22-Abstracts-Book.pdf.

[6] IMpact-RSV Study Group. Palivizumab, a humanized respiratory syncytial virus monoclonal antibody, reduces hospitalization from respiratory syncytial virus infection in high-risk infants. The IMpact-RSV Study Group. Pediatrics. 1998;102:531–537.

[7] ClinicalTrials.gov. A Phase 2b Randomized, Double-Blind, Placebo-controlled Study to Evaluate the Safety and Efficacy of MEDI8897, a Monoclonal Antibody With an Extended Half-life Against Respiratory Syncytial Virus, in Healthy Preterm Infants [Internet]. 2019. Available from: https://clinicaltrials.gov/ct2/show/NCT02878330.

[8] Griffin MP, Yuan Y, Takas T, et al. Single-Dose Nirsevimab for Prevention of RSV in Preterm Infants. N Engl J Med. 2020;383:415–425.

[9] ClinicalTrials.gov. A Phase 2/3 Randomized, Double-blind, Palivizumab-controlled Study to Evaluate the Safety of MEDI8897, a Monoclonal Antibody With an Extended Half-life Against Respiratory Syncytial Virus, in High-risk Children (MEDLEY), NCT03959488 [Internet]. 2022. Available from: https://clinicaltrials.gov/ct2/show/NCT03959488.

[10] ClinicalTrials.gov. A Phase 3 Randomized, Double-blind, Placebo-controlled Study to Evaluate the Safety and Efficacy of MEDI8897, a Monoclonal Antibody With an Extended Half-life Against Respiratory Syncytial Virus, in Healthy Late Preterm and Term Infants (MELODY), NCT03979313 [Internet]. 2022. Available from: https://clinicaltrials.gov/ct2/show/NCT03979313.

[11] Hammitt LL, Dagan R, Yuan Y, et al. Nirsevimab for Prevention of RSV in Healthy Late-Preterm and Term Infants. N Engl J Med. 2022;386:837–846.

[12] Simões EAF, Madhi SA, Muller WJ, et al. Efficacy of nirsevimab against respiratory syncytial virus lower respiratory tract infections in preterm and term infants, and pharmacokinetic extrapolation to infants with congenital heart disease and chronic lung disease: a pooled analysis of randomised controlled trials. Lancet Child Adolesc Health. 2023;7:180–189.

[13] Domachowske J, Madhi SA, Simões EAF, et al. Safety of Nirsevimab for RSV in Infants with Heart or Lung Disease or Prematurity. N Engl J Med. 2022;386:892–894.

[14] Agencia Española de Medicamentos y Productos Sanitarios (AEMPS). Ficha técnica de palivizumab (Synagis®) [Internet]. 2022. Available from: https://cima.aemps.es/cima/dochtml/ft/199117004/FT_199117004.html.

[15] EMA. Synagis [Internet]. Eur. Med. Agency. 2018 [cited 2021 Jul 26]. Available from: https://www.ema.europa.eu/en/medicines/human/EPAR/synagis.

[16] AEMPS. Synagis®: Ficha técnica o resumen de las características del producto [Internet]. 2009 [cited 2021 May 19]. Available from: https://cima.aemps.es/cima/dochtml/ft/199117003/FT_199117003.html.

[17] Sánchez Luna M, Pérez Muñuzuri A, Leante Castellanos JL, et al. Recomendaciones de la Sociedad Española de Neonatología para la utilización de palivizumab como profilaxis de las infecciones graves por el virus respiratorio sincitial en lactantes de alto riesgo, actualización. An Pediatría. 2019;91:348–350.

[18] Sanofi. Nirsevimab EMA regulatory submission accepted under accelerated assessment for RSV protection in all infants [Internet]. 2022. Available from: https://www.sanofi.com/media-room/press-releases/2022/2022-02-17 14-00-00 2387084.

[19] Nuijten MJ, Wittenberg W. Cost effectiveness of palivizumab in Spain: an analysis using observational data. Eur J Health Econ HEPAC Health Econ Prev Care. 2010;11:105–115.

[20] INE. Actualización de rentas con el IPC general (sistema IPC base 2021) para periodos anuales completos [Internet]. 2022. Available from: https://www.ine.es/calcula/.

[21] INE. Movimiento natural de la población: Nacimientos [Internet]. 2021. Available from: https://www.ine.es/dyngs/INEbase/es/operacion.htm?c=Estadistica_C&cid=1254736177007&menu=ultiDatos&idp=1254735573002.

[22] BotPlus. Base de datos de medicamentos - PVL [Internet]. 2022. Available from: https://botplusweb.portalfarma.com/.

[23] Martinón-Torres F, Carmo M, Platero L, et al. Clinical and economic burden of respiratory syncytial virus in Spanish children: the BARI study. BMC Infect Dis. 2022;22:759.

[24] Instituto Nacional de Estadística. Datos del padrón continuo. 2021.

[25] Pokrzywinski RM, Swett LL, Pannaraj PS, et al. Impact of Respiratory Syncytial Virus–Confirmed Hospitalizations on Caregivers of US Preterm Infants. Clin Pediatr (Phila). 2019;58:837–850.

[26] Garcia-Marcos L, Valverde-Molina J, Pavlovic-Nesic S, et al. Pediatricians’ attitudes and costs of bronchiolitis in the emergency department: a prospective multicentre study. Pediatr Pulmonol. 2014;49:1011–1019.

[27] Leader S, Yang H, DeVincenzo J, et al. Time and Out-of-Pocket Costs Associated with Respiratory Syncytial Virus Hospitalization of Infants. Value Health. 2003;6:100–106.

[28] OMS. Health Equity -- Global [Internet]. [cited 2022 May 20]. Available from: https://www.who.int/health-topics/health-equity.

[29] Mac S, Sumner A, Duchesne-Belanger S, et al. Cost-effectiveness of Palivizumab for Respiratory Syncytial Virus: A Systematic Review. Pediatrics. 2019;143:e20184064.

1. non-inferiority test, where efficacy is seen only as an exploratory goal [↑](#footnote-ref-2)
2. RSV population *vs.* RSV + bronchiolitis population [↑](#footnote-ref-3)
3. 29-35 weeks gestational age. [↑](#footnote-ref-4)
